# NIH funding for patents that contribute to market exclusivity of drugs approved 2010–2019 and the public interest protections of Bayh-Dole

**DOI:** 10.1371/journal.pone.0288447

**Published:** 2023-07-26

**Authors:** Fred D. Ledley, Ekaterina Galkina Cleary

**Affiliations:** 1 Center for Integration of Science and Industry, Bentley University, Waltham, Massachusetts, United States of America; 2 Department of Natural & Applied Sciences, Bentley University, Waltham, Massachusetts, United States of America; 3 Department of Management, Bentley University, Waltham, Massachusetts, United States of America; 4 Department of Mathematical Sciences, Bentley University, Waltham, Massachusetts, United States of America; Maynooth University, IRELAND

## Abstract

Previous studies have shown that National Institutes of Health (NIH) funding contributed >$187 billion for basic or applied research related to the 356 drugs approved 2010–2019. This analysis asks how much of this funding led to patents cited as providing market exclusivity, patents that would be subject to the provisions of the Bayh-Dole Act that promote and protect the public interest. The method involves identifying published research in PubMed related to the approved drugs (applied research) or their targets (basic research). NIH-funded projects (grants) funding these publications and patents arising from these projects were both identified in RePORT. Patents cited as providing market exclusivity were identified in DrugPatentWatch (which incorporates FDA Orange Book). NIH funded basic or applied research related to all 313 FDA-approved drugs 2010–2019 with at least one patent in DrugPatentWatch. This research comprised 350 thousand publications (9% applied research; 91% basic research) supported by 341 thousand fiscal years (project years) of NIH funding and $164 billion in NIH project year costs (17% applied research; 83% basic research). These NIH projects also produced 22,360 patents, 119 of which were cited in DrugPatentWatch as protecting 34/313 drugs. These patents were associated with 769 project years of NIH funding (0.23% total) and project year costs of $0.95 billion (0.59% total). Overall, only 1.5% of total NIH funding for applied research and 0.38% of total NIH funding for basic research was associated with patents in DrugPatentWatch. This analysis shows that very little of the NIH funding for research that contributes to new drug approvals leads to patents that provide market exclusivity and are subject to the provisions of the Bayh-Dole Act that promote the public interest in practical applications of the research, reasonable use and pricing, and a return on this public sector investment. This suggests that the Bayh-Dole Act is limited in its ability to protect the public interest in the pharmaceutical innovations driven by NIH-funded research.

## Introduction

Previous studies have shown that the National Institutes of Health (NIH) contributed $187 billion for basic or applied research related to the 356 drugs approved 2010–2019 [1, 2], 99.4% of which were subsequently developed and launched by biopharmaceutical companies [1, 2]. This NIH investment complements the estimated $1.03–1.4 billion/drug spent by biopharmaceutical companies to develop these products [3, 4]. Once launched, the companies that sponsor these drugs typically have a period of market exclusivity protected, in part, by patents on the drug product, its function, or its utility. These patents can arise from research or development performed by biopharmaceutical companies or from research performed in the public sector, including research funded by the NIH.

This study asked two questions. First, how many of the drugs approved 2010–2019 were associated with patents that arose from NIH-funded research and represent barriers to entry of competing products? These patents potentially protect market exclusivity, would be of substantial importance to companies launching these products, and would be subject to the provisions of the Bayh-Dole Act that governs the licensing of patents arising from NIH-funded research to industry. Second, how much of the total NIH funding for basic or applied research performed on these products prior to first drug approval was represented in these patents? The provisions of the Bayh-Dole Act were designed to promote the public’s interest in practical applications of government-funded research and to protect the public against nonuse or misuse, ensure reasonable pricing, and provide the public sector with a return on investment. These provisions represent the only substantive protections for the public’s interest in the benefits that may accrue from government-funded research. In this context, the fraction of the NIH funding for basic or applied research related to approved drugs is a measure of the scope and applicability of the Bayh-Dole Act and its ability to serve the public interest.

### Background

Targeted drug discovery arises from a foundation of basic research describing mechanisms of biology and disease, targets for drug discovery, or sometimes prototype products [1, 2, 5–10]. This research typically takes place in academic or government laboratories with public sector funding. In the U.S., this funding is provided primarily by the NIH [7, 8, 11–14].

A series of studies by Cleary et al. [1, 2] demonstrated that NIH funding contributed to published research related to 354/356 new drugs approved by the FDA 2010–2019 or their targets. This funding comprised 360 thousand fiscal years of project (grant) funding and $187 billion total costs [2]. Published research on targets, representing basic research, comprised 88% of fiscal years of project funding and 83% of costs. The remainder was associated with research on the drugs themselves and was classified as applied research. Combined, this funding resulted in 22,360 patents [2].

There are several mechanisms by which NIH-funded research informs subsequent product development in industry. Academic research is traditionally disseminated through publication, presentation, or training students for the workforce with knowledge and know-how arising from scholarly activities. Research is also communicated directly to industry through academic-industry partnerships or consulting [15–22]. In addition, patents arising from federally funded research may be licensed to industry for commercialization through the provisions of the “Patent and Trademark Law Amendments Act” of 1980, known as the Bayh-Dole Act [23–26].

The objectives of the Bayh-Dole Act are to “…*promote the utilization of inventions arising from federally supported research or development [and] …the commercialization and public availability of inventions made in the United States by United States industry and labor…*,” while also protecting “*…the public against nonuse or unreasonable use of inventions”* [27]. The provisions of the Act are triggered by disclosure of a “subject invention” arising from federally funded research [28]. This disclosure obligates the host institution to assess the patentability of the invention and its commercial potential. Bayh-Dole also authorizes academic institutions to file patents on the invention and enter exclusive or non-exclusive licenses with companies to commercialize practical applications [24–28]. Significantly, the provisions of Bayh-Dole do not apply generally to federally funded research, but only to research that results in a disclosure of a subject invention or a patent [29].

Patents that provide new products with market exclusivity are particularly valuable to the companies that develop and launch these products. The “Drug Price Competition and Patent Term Restoration Act” (Hatch-Waxman Act) requires companies to identify patents that might be infringed by the approval of a generic version of an existing product [30, 31]. Under the Hatch-Waxman Act, companies launching a new drug (but not biological) product identify the patents that protect their product, which are then listed in the FDA Orange Book. Companies seeking to develop a competing, generic equivalent of a product must certify that each of the patents listed in the FDA Orange Book is expired, invalid, or unenforceable, or that the generic would not infringe the patent [30–34]. While the role of patents is similar in the regulations governing developing biosimilar products under the Biologics Price Competition and Innovation Act of 2009 [35], these patents are not listed in the FDA Orange Book. Moreover, additional patents may be identified during litigation concerning market exclusivities or infringement. In order to capture as many patents as possible, this work utilized a commercial database maintained by DrugPatentWatch, which incorporates the FDA Orange Book as well as certain other patents identified from ongoing litigations or other sources [36].

This study asked how much of the NIH funding for basic or applied research related to recent drug approvals is associated with patents in the DrugPatentWatch database. In doing so, we describe the reach of the Bayh-Dole Act and its provisions designed to promote and protect the public interest in practical applications of this research.

## Methods

### Materials

Drugs approved by the FDA 2010–2019, including products approved by both the Center for Drug Evaluation and Research (CDER) and the Center for Biologics Evaluation and Research (CBER), and their targets were identified from FDA reports as described previously [1]. Products derived from blood or tissue, diagnostic products, and vaccines were excluded. The DrugPatentWatch dataset (https://www.drugpatentwatch.com/) includes active, expired, and deleted patents from the FDA Orange Book (https://www.accessdata.fda.gov/scripts/cder/ob/index.cfm) (including entries removed in March 2020) as well as certain additional patents identified by DrugPatentWatch [36].

Research publications were identified in PubMed (https://pubmed.ncbi.nlm.nih.gov/). Search terms related to both the drug and to the targets are shown in S1 Table. Results are indexed by PubMed Identifier (PMID) and publication year. NIH funding was identified in the Research Portfolio Online Reporting Tools (RePORT) (https://reporter.nih.gov/) [37].

### Design

NIH funding for published research on approved drugs or their targets was identified using a modification of methods described previously [1, 2]. Briefly, the method involves identifying publications related to the drug or its target by searching PubMed. PMIDs identified by searching for the target, but not the drug, are classified as “basic research.” Those identified by searching for the drug name are classified as “applied research.” NIH funding for this research is then identified by linking these PMIDs with NIH-funded projects in NIH RePORT. NIH costs are estimated as one year of each identified project corresponding to the publication year of the PMID after accounting for lags of 1–4 years after the end of project funding.

Patents resulting from each NIH-funded project are also identified in RePORT. Patents cited as providing each drug with market exclusivity were identified in DrugPatentWatch. Patents cited as providing these drugs with market exclusivity that arose from NIH-funded research were identified by joining these two patent lists.

### Analysis

Publications in PubMed from 1960 through the date of first FDA approval of each drug were identified by searching for the drug name or its target using MeSH terms, updated Automatic Term Mapping (May 2020), or Boolean search terms. NIH projects funding each PMID were identified using the NIH RePORT Link tables. Project years represent the fiscal year corresponding to the PMID’s publication date. PMIDs with publication dates before the first fiscal year of project funding were excluded. PMIDs with dates 1–4 years after the final year of the project were assigned a project year corresponding to the last year of project funding to account for observed lags between funding and publication [37]. Control studies have been performed showing that this method produces equivalent results when incorporating various lags between the date of funding and date of publication [1]. Duplicate PMIDs, project years, costs, and patents were eliminated to identify “unique” entries. NIH funding was identified from 2000–2019 and was calculated either as “total costs,” i.e., the sum of costs for every fiscal year of a project, or “project year costs,” i.e., the sum of costs for project years only.

Patents in DrugPatentWatch were identified by searching for the approved drug name. NIH-funded patents associated with each of the projects funding basic or applied research on these drugs were identified from Project Details in RePORT. NIH costs contributing to a patent are described in RePORT either as: (i) total costs of the associated project (all fiscal years), or (ii) only costs of project years corresponding to publication of at least one PMID related to the drug or its target (“project years”). The method is described in greater detail in S1 File.

Data analysis and visualization were performed in PostgreSQL and Excel. Costs are inflation-adjusted to 2018 using the U.S. Bureau of Labor Statistics’ consumer price index (CPI) (https://www.bls.gov/cpi/data.htm). This method for identifying NIH-funded projects contributing to basic or applied research related to approved drugs has been described in an extensive eMethods supplement in conjunction with Galkina Cleary et al. (https://jamanetwork.com/journals/jama-health-forum/fullarticle/2804378). Since completion of this work, this method has been replicated in Python code that is freely available at https://github.com/BentleySciIndustry/NIH-Contribution-to-phased-clinical-development-of-drugs-approved-Supplemental-Data-Sharing. This method can be accessed through a dashboard at https://www.bentley.edu/centers/center-integration-science-and-industry/nih-fundingdrug-innovation-dashboard.

## Results

### NIH funding for patents related to drugs approved 2010–2019

**Fig 1:**
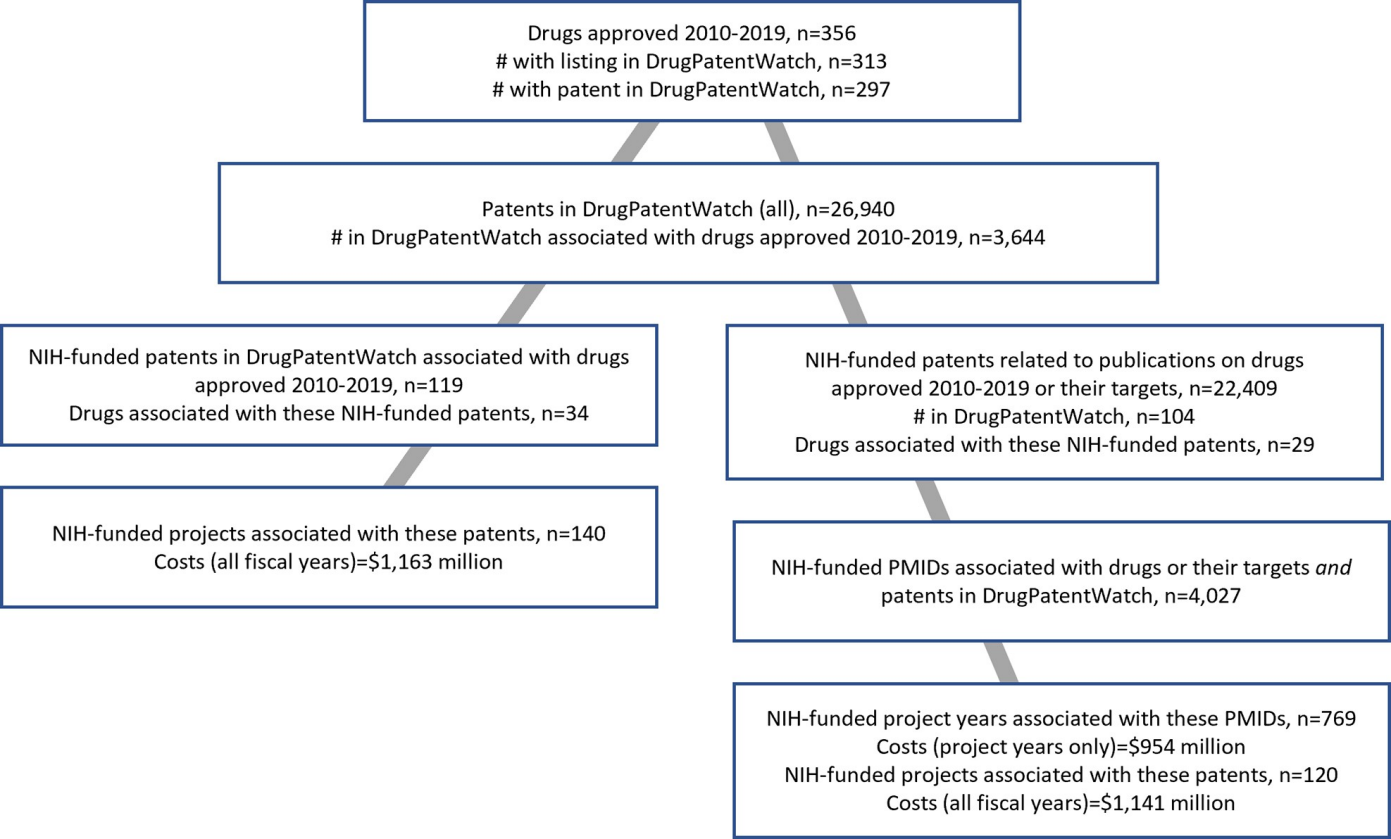
Schematic of experimental design and results.

A schematic of the analysis and results is shown in Fig 1.

From 2010–2019, 356 drugs approved by the FDA met the conditions for inclusion in this analysis [2], including 313 listed in FDA Orange Book or DrugPatentWatch and 297 that had at least one patent in DrugPatentWatch (S1 Table). DrugPatentWatch comprises 26,579 patents. Of these, 3,644 patents were associated with these drugs (Table 1).

**Table 1:** Number of new drug approvals 2010–2019 associated with NIH-funded patents.

	# drugs	
Drugs approved 2010–2019	356	
…with entry in FDA Orange Book or DrugPatentWatch^1^	313	
…with at least one patent in FDA Orange Book or DrugPatentWatch	297	
	# patents	
…associated patents in FDA Orange Book or DrugPatentWatch	3,644	
	RePORT	Cleary dataset^2^
NIH-funded patents related to drugs approved 2010–2019	n/a	22,409
…number in FDA Orange Book or DrugPatentWatch (% associated patents)^3^	119 (3.3%)	104 (2.9%)
	# drugs	
Drugs with NIH-funded FDA Orange Book or DrugPatentWatch patents (% drugs)^4^	34 (10.9%)	29 (9.3%)

^1^ DrugPatentWatch includes all active and expired patents from the FDA Orange Book and certain additional patents on biological products identified by companies or patent search.

^2^ Cleary identified NIH-funded projects associated with drugs approved 2010–2019 or their targets as well as patents arising from these projects [2].

^3^ Percentage of patents in FDA Orange Book or DrugPatentWatch associated with drugs approved 2010–2019.

^4^ Percentage of drugs approved 2010–2019 listed in FDA Orange Book or DrugPatentWatch. n/a–not applicable.

The NIH RePORT database identified NIH-funded projects (2000–2019) associated with 119/3,644 (3.3%) of these patents (Table 1). These patents were cited as potential barriers to entry for 34 of the 313 (10.9%) products with at least one patent in DrugPatentWatch (Table 1, S1 Table). The 119 patents were associated with 104 NIH-funded projects in NIH RePORT with $1,163 million total costs (all project years) from 2000–2019 (Table 2).

**Table 2:** Research focus of funding producing patents in DrugPatentWatch associated with drugs approved 2010–2019.

	Total^1^	Applied research (% total)			Basic research (% total)	
**NIH-funded research on drugs approved 2010–2019 listed in DrugPatentWatch (n = 313)**						
Searches	n/a		313		194	
PMIDs	349,797		33,048	(9%)	316,749	(91%)
Project Years^2^	341,375		45,200	(13%)	296,175	(87%)
Project Year Costs (project funding years only, millions)	$163,878		$28,553	(17%)	$135,325	(83%)
Projects	145,441		26,872	(18%)	118,569	(82%)
Total Project Costs (all project years, millions)	$339,979		$114,882	(34%)	$225,097	(66%)
**NIH-funded research related to DrugPatentWatch patents associated with drugs approved 2010–2019**						
Patents	104		61	(59%)	43	(41%)
PMIDs	4,030		546	(14%)	3,484	(86%)
Project Years^2^	769		242	(31%)	527	(69%)
Project Year Costs (project funding years only, millions)	$954.0		$436.7	(46%)	$517.3	(54%)
Projects	120		61	(51%)	59	(49%)
Total Project Costs (all project years, millions)	$1,142.3		$940.0	(82%)	$202.4	(18%)

^1^ Includes both applied and basic research.

^2^ NIH RePORT data 2000–2019.

### Patents associated with NIH funding for research related to approved drugs or their targets

The 313 drugs in this study were associated with 349,797 NIH-funded publications identified by searching for the drugs or their targets prior to first approval. These PMIDs were associated with 145,441 NIH-funded projects with $339,979 million total project costs. Of these totals, 120 projects (0.08%), with $1,142 million total project costs (0.34%), were also associated with patents related to these drugs in DrugPatentWatch (Table 2).

Considering only those fiscal years of funding (project years) associated with published research (PMIDs) related to the 313 drugs or their targets, NIH funding comprised 341,375 project years and $163,878 million project year costs. Of this amount, 769 project years of funding (0.23% of all project years) with $954 million project year costs (0.58% of all project year costs) were associated with projects that also produced patents related to these drugs in DrugPatentWatch (Tables 2 and 3). These project years were associated with 29/313 (9.3%) of the products with at least one patent in DrugPatentWatch (Table 1, S1 Table) and accounted for 1.1% of all PMIDs related to these drugs (Table 3). This funding, which was focused explicitly on the drug or its target, accounted for 104/119 (87.4%) patents associated with these products in DrugPatentWatch (Table 1, S2 Table). The other 15 patents identified in DrugPatentWatch in association with these products related to production, formulation, or product use, rather than the drug or its target (S2 Table).

**Table 3:** Percentage of NIH investments in published research on drugs approved 2010–2019 or their targets associated with patents in DrugPatentWatch.

	Total^1^		Applied research^2^		Basic research^3^	

	% ^4^	(patents,^5^ all^6^)	% ^4^	(patents,^5^ all^6^)	% ^4^	(patents,^5^ all^6^)
PMIDs (thousands)	1.2%	(4.0, 349.8)	1.7%	(0.55, 33.0)	1.1%	(3.5, 316.7)
Project Years (thousands)	0.23%	(0.77, 341.4)	0.54%	(0.24, 45.2)	0.18%	(0.53, 296.2)
Project Year Costs (billions)	0.59%	($0.95, $163.9)	1.5%	($0.44, $28.6)	0.38%	($0.52, $135.3)
Total Project Costs (billions)	0.34%	($1.14, $339.9)	0.82%	($0.94, $114.9)	0.090%	($0.20, $225.1)

^1^ All NIH-funded research associated with drugs approved 2010–2019.

^2^ Research identified searching for the drug.

^3^ Research identified by searching for the target, but not the drug.

^4^% of total associated with patents.

^5^ # associated with 104 patents in DrugPatentWatch.

^6^ # associated with published research.

### Basic and applied research contributing to patents

Using the method of Cleary et al. [1, 2], PMIDs identified by searching for drug names were classified as applied research. The project years supporting this research, along with the full project and any patents arising from this project, were similarly classified as applied research. Conversely, PMIDs identified by searching for drug targets, but not drugs, were classified as basic research. Project years or projects were classified as basic research if all of the associated PMIDs related to drug targets, as were any patents associated with such projects.

These data confirm the observations of Cleary et al. [1, 2] that a large majority of NIH funding focuses on basic research, rather than applied research. For the 313 drugs with at least one patent in DrugPatentWatch, only 9% of all PMIDs, 13% of project years, and 17% of project year costs were classified as applied research (Table 2). Considering the full term and total cost of these projects, 18% of the NIH-funded projects and 34% of costs were classified as applied research.

**Fig 2:**
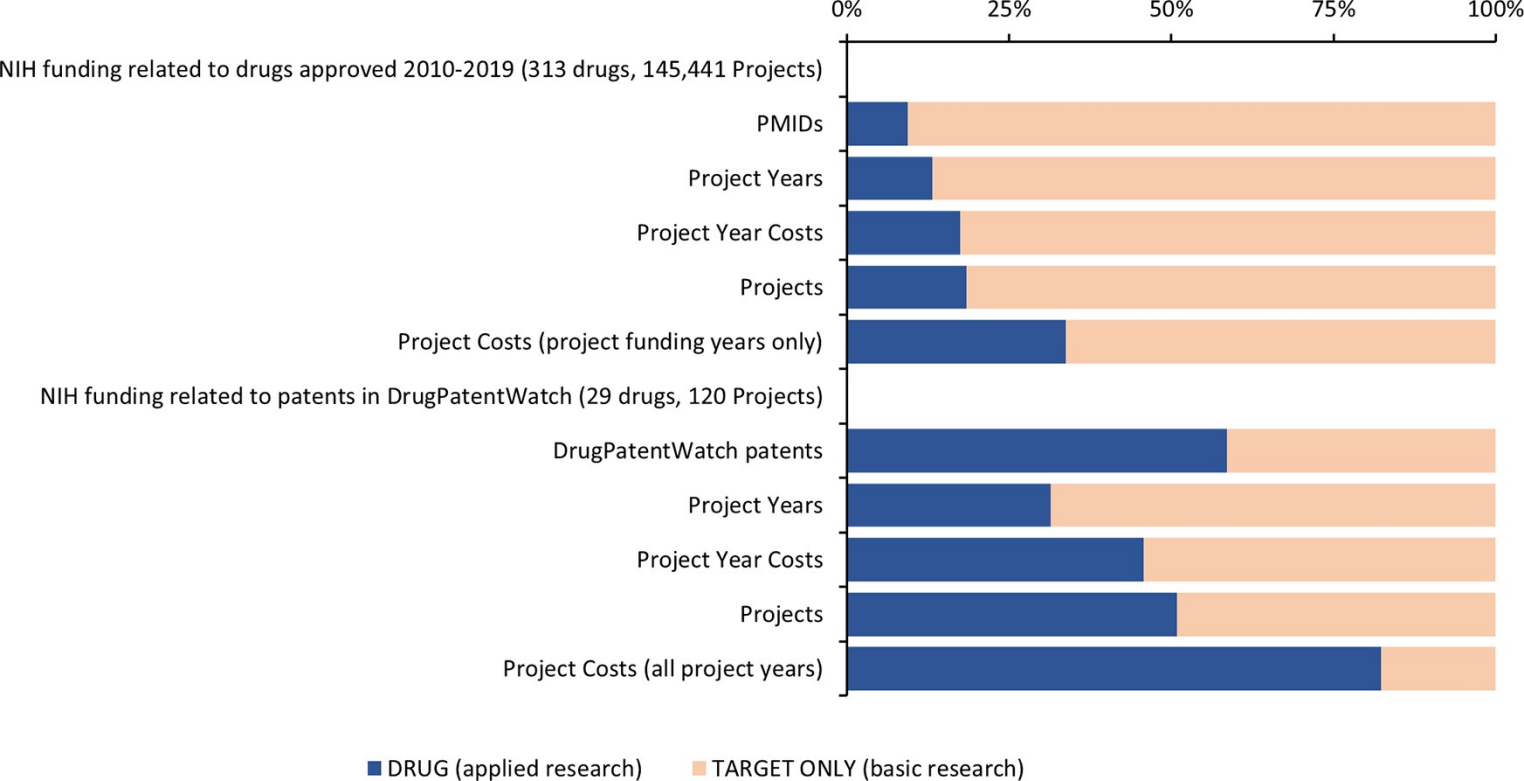
Research focus of PMIDs, project years, projects, and patents associated with drugs approved 2010–2019.

For those projects that also resulted in patents in DrugPatentWatch, 31% of project years and 46% of project year costs were classified as applied research, while 51% of the full projects (all years) and 82% of total project costs were classified as applied research (Table 2, Fig 2). Thus, while the large majority of NIH funding associated with these products focused on basic research related to their target, patents arise disproportionately from projects involving applied research on the drug itself.

Project years associated with patents in DrugPatentWatch comprised 0.54% of project years and 1.5% of project year costs classified as applied research, but only 0.18% of project years and 0.38% of project year costs classified as basic research (Table 3). Considering the full term of these projects (all years), patents were associated with 0.82% of the project costs classified as applied research but only 0.09% of total project costs classified as basic research (Table 3).

## Discussion

This study examines NIH funding for basic or applied research related to the drugs approved 2010–2019 and the patents resulting from this research cited as potential barriers to generic or biosimilar competition. Such patents contribute directly to the sponsor’s monopoly power to set drug prices and profit without market competition. Those patents arising from NIH-funded research are also subject to the provisions of the Bayh-Dole Act designed to promote and protect the public’s interest in practical applications of this research.

This work asked two questions. First, how many of the drugs approved 2010–2019 were associated with patents that arose from NIH-funded research and represent barriers to entry of competing products? These results show that 10.9% of the drugs approved 2010–2019 had at least one NIH-funded patent listed in DrugPatentWatch as a potential barrier to entry for competition. This fraction is similar to the results of previous studies that have used NIH-funded patents in the FDA Orange Book as a proxy for the government’s contribution to pharmaceutical innovation. For example, Sampat and Lichtenberg examined “public sector” or “academic” patents related to drugs approved 1988–2005, finding that 9% of new drugs were associated with a public sector patent [7]. Similarly, Long reported that 8.6% of drugs had at least one patent with a “government-interest” statement [38] and Stevens et al. estimated that from 1990–2007, 9.3% of new drugs were first patented in public sector research institutions [39].

Second, how much of the total NIH funding for basic or applied research performed on these products prior to first drug approval was represented in these patents? This analysis shows that only 0.59% of the >$164 billion in NIH funding for research related to the drugs approved from 2010–2019 is associated with patents in DrugPatentWatch. These results are consistent with the observation of Li et al. that <1% of NIH grants are directly acknowledged by patents listed in the FDA Orange Book [40].

### Disparity between NIH contribution to pharmaceutical innovation and patents

The finding that only a small fraction of the NIH funding for basic or applied research related to the drugs approved 2010–2019 belies extensive evidence that NIH-funded basic or applied research plays a critical role in pharmaceutical innovation. Recent research shows that 19% of the new drugs approved 2008–2018 had their origin in publicly funded, applied research [41]. Other studies have demonstrated that as many as 55% of the 1,453 drugs approved by the FDA through 2014 were first synthesized or purified in academic institutions [42], that 76% of the drugs with the most impact on practice from 1965–1992 had input from the public sector [43], that 47.8% of drugs approved 1988–2005 were associated with patents that cited prior art from the public sector [7], and that 54% of the basic science milestones for the “most transformative” drugs 1992–2016 were achieved in the public sector [8]. Our previous studies, confirmed in the present work, have identified NIH-funded basic or applied research associated with >99% of drug approvals 2010–2019 [1, 2]. These studies also show that an established body of basic research on drug targets, much of which is funded by the NIH, is associated with increased efficiency of drug development. Other studies have demonstrated that sector-specific NIH funding is associated with increases in the number of private sector patents [44] as well as an increased number of clinical trials [45].

The present results suggest several explanations for the disparity between the demonstrated role of NIH funding in pharmaceutical innovation and the limited representation of NIH-funded patents in DrugPatentWatch.

First, these data show that NIH funding produces substantially more published basic research on the targets of the drugs approved 2010–2019 than published applied research on the drugs themselves [1, 2]. Basic research may be defined as “*…experimental or theoretical work undertaken primarily to acquire new knowledge of the underlying foundations of phenomena and observable facts*, *without any particular application or use in view*,” [46] though it is also recognized that basic research may be “*use inspired*” and undertaken with the expectation of bettering human health [47]. In contrast, applied research is *“…directed primarily towards a specific*, *practical aim or objective*” [46] and development is “…*directed to producing new products or processes or to improving existing products or processes*” [46].

This distinction is significant because the provisions of the Bayh-Dole Act that promote practical applications of NIH-funded research are only triggered by disclosure of a “subject invention.” The Bayh-Dole Act defines a subject invention as “*…any invention of a contractor conceived or first actually reduced to practice in the performance of work under a funding agreement*” and further requires that i*t must be “conceived or first actually reduced to practice in performance of the project*” [27]. Moreover, a patent requires the applicant to be able to “*…teach those skilled in the art how to make and use the full scope of the claimed invention without ‘undue experimentation’*” [48]. Since basic research is not inherently concerned with establishing the utility of any specific product [46, 47], funding for basic research may be less likely to produce patents than applied or translational research. This dynamic is evident in the present analysis, which shows that NIH-funded applied research on the approved drugs was more likely to generate patents in DrugPatentWatch than NIH-funded basic research on its target. We would also note that, even if patents are issued on NIH-funded basic research, unless these patents are cited as protecting a product’s market exclusivity, the applicability of Bayh-Dole is moot since a patent license is not necessary to commercialize that product.

Second, the legal standard for an invention posits a model of innovation involving discrete, independent advances that proceed in a linear manner. While inventions may be informed by previous findings (the “prior art”), they must be both novel and non-obvious in the context of that art. Moreover, the 2011 Leahy-Smith America Invents Act introduced a “first-to-file” standard that recognizes the first individual to file a patent as the inventor, not necessarily the researchers who make the seminal discoveries. These standards explicitly preclude patenting of research that reproduces, refutes, or refines previous studies, studies that are central to successful translation of scientific advances [49, 50] and are contrary to the demonstrated high frequency of contemporaneous inventions [51] and the view of scientific research as an open, often cooperative enterprise. This standard may contribute to the observation that very little of the NIH contribution to the body of knowledge that is essential for successful pharmaceutical development [2, 9] is represented by the patents listed in DrugPatentWatch.

Third, while not explicitly examined in this report, Cleary et al. have shown that up to a third of the NIH funding associated with the drugs approved 2010–2019 was in the form of Program Project or Center programs or various training or career development programs [2]. While these programs provide substantial funding and resources for both basic and applied research, this research would not be identified being “conceived or first actually reduced to practice” in performance of these products and typically would not generate patents.

Finally, it should be noted that there is an inherent tension in academia between the open dissemination of new knowledge and the patenting and commercialization of that knowledge [15–20]. Even if patentable, much of the knowledge arising from NIH-funded research may be transferred to industry through scientific publications or presentations, training students for the industrial workforce, or interactions between academic and industry researchers [25, 52].

The primary societal function of patents is to provide the inventor or licensee of the patent with a term-limited monopoly on the use of that invention, while also enabling the patented information to be widely disseminated. The fact that the Bayh-Dole Act applies only to subject inventions or patents arising from government-funded research also makes patents central to the primary policy mechanism intended to ensure public benefit from that research.

### Public protections of the Bayh-Dole Act

The Bayh-Dole Act is intended to promote the public interest by enabling the development and commercialization of the “*practical applications*” enabled by government-funded research along with the jobs, economic growth, and expanded tax base resulting from commercialization of these applications. Additionally, the act provides a mechanism for the public to realize a return on the government’s investment of taxpayer dollars by positioning non-profit organizations as proxies for the public sector and authorizing them to seek commercial license agreements and reinvest the proceeds from such licenses in their public mission of education, research, or service [28]. Bayh-Dole also provides protection for the public interest by granting government nonexclusive rights to practice the invention, protection “…*against nonuse or unreasonable use of inventions*” [27] and “march in rights” that enable government to authorize alternate licenses to ensure the benefits of the invention are “*to the extent permitted by law or government regulations*, *available to the public on reasonable terms*” [53–57].

The present analysis suggests that >99% of the NIH funding for basic or applied research related to the drugs approved from 2010–2019 may not be covered by these protections. Moreover, this work also suggests that the Bayh-Dole Act is structurally limited in its ability to protect the public interest in the fruits of NIH research by its focus on subject inventions and patents, which typically arise from the applied research required to establish utility and enable reduction to practice, rather than from basic research classically conducted without a product in mind. This limitation, coupled with the fact that >85% of the NIH funding associated with new drug approvals represents basic research, severely limits the ability of the Bayh-Dole Act to serve the public’s interest in pharmaceutical innovation.

A recent paper compared the discounted value of the NIH investment in new drug approvals to analogous estimates of the investments made by industry [58]. The work demonstrated that the public sector investment by the NIH was not less than the investments made by the private sector. The present work suggests that policies designed to promote the public interest in practical applications of the research enabled by this investment, protect against nonuse or unreasonable pricing of these applications, and provide the public sector a return on investment cannot be based solely on “inventions” or patents. Rather, such policies need to recognize the broader public sector contributions to biomedical research and development that underlie pharmaceutical innovation.

### Limitations

First, neither the FDA Orange Book nor DrugPatentWatch represent a comprehensive list of patents comprising barriers to entry of competing products. While the Hatch-Waxman Act requires companies to identify patents that may block generic competition for chemical entities, no similar statutory requirement exists for biological products.

Second, this analysis is limited by the sensitivity and specificity of the PubMed search [1, 2]. While various search methods (Boolean searches, MeSH subject headings, and Automated Term Mapping) were used to optimize search results, each of these methods may fail to identify salient published research [1, 59].

Third, the utility of the RePORT database is limited by the fidelity of authors acknowledging NIH support for their research, the absence of funding from contracts and some federal agencies, and the observation that it contains both false positive and false negative associations between PMIDs and NIH-funded projects [37]. The method for associating publications with fiscal years of funding is subject to uncertain assumptions about years of funding associated with each publication and lags between funding and publication. The method used here, which associates each publication with one fiscal year of project funding (project year), is consistent with evidence that five-year NIH grants produce a median of five publications [60]. This method may, however, underestimate the cost of studies spanning multiple years and may overestimate the contribution of Program Project or Center grants that provide core facilities.

Fourth, there is no evidence that the NIH-funded patents identified in this analysis were licensed to industry through Bayh-Dole. While recipients of federal funding are required to report subject inventions, patents, or licenses of federally funded inventions to Interagency Edison (iEdison) maintained by the National Institute of Standards & Technology, public access to these data is explicitly restricted by the Bayh-Dole Act (§ 209(d)(2)) [61, 62].

## Conclusion

This analysis shows that very little of the NIH funding for research that contributes to new drug approvals leads to patents that provide market exclusivity and are subject to the provisions of the Bayh-Dole Act that promote the public interest in practical applications of the research, reasonable use and pricing, and a return on this public sector investment. This suggests that the Bayh-Dole Act is limited in its ability to protect the public interest in the pharmaceutical innovations driven by NIH-funded research.

## Supporting information

S1 Table Drugs approved 2010–2019 with patents in DrugPatentWatch. Patent: * at least one patent in DrugPatentWatch (n = 263); ** at least one patent in DrugPatentWatch associated with NIH-funded project supporting research on drugs or drug targets (n = 5); *** at least one patent in DrugPatentWatch also listed in RePORT (any research topic) (n = 29). Drugs with no patents in DrugPatentWatch: n = 16. Target Search Term: search used to identify research on a drug target. Research on drugs was identified by searching for brand name, generic name, or known synonyms.
(DOCX)

S2 Table NIH-funded patents associated with drugs approved 2010–2019 in DrugPatentWatch. A. Patents in RePORT with associated publication related to drugs or drug targets (n = 104); B. Patents in RePORT but without associated publication related to drugs or drug targets (n = 15).
(DOCX)

S3 Table Patents in DrugPatentWatch associated with drugs approved 2010–2019. This list of patents associated with the drugs studied in this report is copyrighted material from DrugPatentWatch, reproduced here with permission.
(DOCX)

S1 File Detailed description of the method. This method for identifying NIH-funded projects contributing to basic or applied research related to approved drugs was described in eMethods of Galkina Cleary et al. 2023, reproduced here with permission.
(DOCX)
