# Supplementary material for: NIH funding for patents that contribute to market exclusivity of drugs approved 2010–2019 and the public interest protections of Bayh-Dole
Source: PLoS One. 2023 Jul 26;18(7):e0288447. doi: 10.1371/journal.pone.0288447 (PMC10370755; doi:10.1371/journal.pone.0288447)
Supplement: S1 Table — Patent: * at least one patent in DrugPatentWatch (n = 263); ** at least one patent in DrugPatentWatch associated with NIH-funded project supporting research on drugs or drug targets (n = 5); *** at least one patent in DrugPatentWatch also listed in RePORT (any research topic) (n = 29). Drugs with no patents in DrugPatentWatch: n = 16. Target Search Term: search used to identify research on a drug target. Research on drugs was identified by searching for brand name, generic name, or known synonyms. (DOCX) [file pone.0288447.s001.docx]

| **S1 Table. Drugs approved 2010–2019 with patents in DrugPatentWatch.** Patent: * at least one patent in DrugPatentWatch (n=263); ** at least one patent in DrugPatentWatch associated with NIH-funded project supporting research on drugs or drug targets (n=5); *** at least one patent in DrugPatentWatch also listed in RePORTER (any research topic) (n=29). Drugs with no patents in DrugPatentWatch: n=16. Target Search Term: search used to identify research on drug targets. Research on drugs was identified by searching for brand name, generic name, or known synonyms. | | | | | |
| --- | --- | --- | --- | --- | --- |
| **Brand Name** | **Patent** | **Generic Name** | **Approval Year** | **Target** | **Target Search Term** |
| Actemra | ^*,**,***^ | Tocilizumab | 2010 | Interleukin-6 receptor subunit alpha | Interleukin 6 receptor |
| Adcetris | ^*,**,***^ | Brentuximab vedotin | 2011 | Tumor necrosis factor receptor superfamily member 8 | CD30 |
| Addyi | ^*^ | Flibanserin | 2015 | 5-hydroxytryptamine receptor 1A; 5-hydroxytryptamine receptor 2A; D4 dopamine receptor | (dopamine receptor) OR serotonin receptor |
| Adempas | ^*^ | Riociguat | 2013 | Guanylate cyclase soluble subunit alpha-2 | "soluble guanylate cyclase" |
| Adlyxin | ^*^ | Lixisenatide | 2016 | glucagon-like peptide 1 receptor | glucagon-like peptide 1 |
| Ajovy | ^*^ | Fremanezumab | 2018 | Calcitonin gene-related peptide 1 and 2 | calcitonin gene-related peptide |
| Aklief | ^*^ | Trifarotene | 2019 | retinoic acid receptor | "retinoic acid receptor" |
| Akynzeo | ^*^ | Netupitant, palonosetron | 2014 | Neurokinin 1 receptor | (substance p receptor) OR tachykinin receptor 1 |
| Akynzeo IV | ^*^ | Palonosetron, fosnetupitant | 2018 | Neurokinin 1 (NK-1) receptor | (substance p receptor) OR tachykinin receptor 1 |
| Alecensa | ^*^ | Alectinib | 2015 | ALK tyrosine kinase receptor | anaplastic lymphoma kinase |
| Aliqopa | ^*^ | Copanlisib dihydrochloride | 2017 | PI3K | Phosphoinositide 3-kinase |
| Alunbrig | ^*^ | Brigatinib | 2017 | FLT3 = class III receptor tyrosine kinase, ALK=receptor tyrosine kinase, ROS1=belongs to tyrosine kinase insulin receptor gene, insulin-like1 = tyrosine kinase receptor | receptor tyrosine kinase |
| Ampyra | ^*^ | Dalfampridine | 2010 | Potassium voltage-gated channel subfamily B member 2; Potassium voltage-gated channel subfamily A member 1; Potassium voltage-gated channel subfamily A member 2; Potassium voltage-gated channel subfamily A member 3; Potassium voltage-gated channel subfamily A member 4; Potassium voltage-gated channel subfamily A member 5; Potassium voltage-gated channel subfamily A member 6; Potassium voltage-gated channel subfamily A member 7; Potassium voltage-gated channel subfamily A member 10; Potassium voltage-gated channel subfamily B member 1; Potassium voltage-gated channel subfamily C member 1; Potassium voltage-gated channel subfamily C member 2; Potassium voltage-gated channel subfamily C member 3; Potassium voltage-gated channel subfamily D member 1; Potassium voltage-gated channel subfamily D member 2; Potassium voltage-gated channel subfamily D member 3 | potassium channel |
| Annovera | ^*^ | Segesterone acetate and ethinyl estradiol vaginal system | 2018 | Estrogen receptor alpha, Estrogen receptor beta | progesterone receptor [MeSH Terms] |
| Anoro Ellipta | ^*^ | Umeclidinium, vilanterol | 2013 | beta-2-adrenergic receptor | beta-2-adrenergic receptor |
| Anthim | ^*^ | Obiltoxaximab | 2016 | protective antigen, Bacillus anthracis; complement component 3b/4b receptor 1 Knops blood group | protective antigen anthrax |
| Aptiom | ^*^ | Eslicarbazepine acetate | 2013 | P2X purinoceptor 4 | voltage gated sodium channel |
| Arcapta neohaler | ^*^ | Indacaterol | 2011 | Beta-2 adrenergic receptor | beta-2-adrenergic receptor |
| Aristada | ^*^ | Aripiprazole lauroxil | 2015 | dopamine receptor D2; 5-hydroxytryptamine serotonin receptor 1A, G-protein-coupled; 5-hydroxytryptamine serotonin receptor 2A, G-protein-coupled | (dopamine receptor) OR serotonin receptor |
| Asclera |  | Polidocanol | 2010 | n/a | PHENOTYPIC-NO SEARCH TERM |
| Aubagio | ^*^ | Teriflunomide | 2012 | Dihydroorotate dehydrogenase quinone, mitochondrial | dihydroorotate dehydrogenase |
| Austedo | ^*^ | Deutetrabenazine | 2017 | Synaptic vesicle monoamine transporter 2 (VMAT2) | monoamine transporter |
| Balversa | ^*^ | Erdafitinib | 2019 | Fibroblast growth factor receptor (FGFR) 1, 2, 3 and 4 | fibroblast growth factor receptor |
| Bavencio | ^*^ | Avelumab | 2017 | Programmed cell death 1 ligand 1 (PD-L1) | programmed cell death-ligand 1 OR PDL1 |
| Beleodaq | ^*^ | Belinostat | 2014 | Histone deacetylase 8; Histone deacetylase 1; Histone deacetylase 10; Histone deacetylase 11; Histone deacetylase 2; Histone deacetylase 3; Histone deacetylase 4; Histone deacetylase 5; Histone deacetylase 6; Histone deacetylase 7; Histone deacetylase 9 | histone deacetylases [MeSH Terms] |
| Belsomra | ^*^ | Suvorexant | 2014 | (orexin aka hypocretin) Orexin receptor type 1; Orexin receptor type 2 | orexin receptor OR hypocretin receptor |
| Belviq | ^*^ | Lorcaserin | 2012 | 5-hydroxytryptamine receptor 2C | 5-HT2C receptor |
| Benlysta | ^*,**,***^ | Belimumab | 2011 | Tumor necrosis factor ligand superfamily member 13B | TNFSF13B |
| Besponsa | ^*,**,***^ | Inotuzumab ozogamicin | 2017 | B-cell receptor CD22 (Antibody-Drug Conjugate) | CD22 |
| Bevyxxa | ^*^ | Betrixaban | 2017 | Coagulation direct factor Xa (Fxa) | factor Xa |
| Biktarvy | ^*^ | Bictegravir, emtricitabine, tenofovir alafenamide | 2018 | n/a | HIV integrase |
| Blincyto | ^*^ | Blinatumomab | 2014 | B-lymphocyte antigen CD19; T-cell surface glycoprotein CD3 delta chain | CD3 |
| Bosulif | ^*^ | Bosutinib | 2012 | Tyrosine-protein kinase SRC | src kinase |
| Braftovi | ^*^ | Encorafenib | 2018 | Serine/threonine-protein kinase B-raf, G1/S-specific cyclin-D1 | braf |
| Breo Ellipta | ^*^ | Fluticasone, vilanterol | 2013 | beta-2-adrenergic receptor | beta-2-adrenergic receptor |
| Bridion | ^*^ | Sugammadex | 2015 | n/a | Rocuronium OR Vecuronium |
| Brilinta | ^*^ | Ticagrelor | 2011 | P2Y purinoceptor 12 | p2y receptor |
| Brintellix/Trintellix | ^*^ | Vortioxetine | 2013 | 5-hydroxytryptamine serotonin receptor | serotonin receptor |
| Briviact | ^*^ | Brivaracetam | 2016 | solute carrier family 8 sodium/calcium exchanger, member 1; synaptic vesicle glycoprotein 2A | PHENOTYPIC-NO SEARCH TERM |
| Brukinsa | ^*^ | Zanubrutinib | 2019 | Bruton's tyrosine kinase (BTK) | bruton's tyrosine kinase |
| Calquence | ^*^ | Acalabrutinib | 2017 | Bruton Tyrosine kinase (BTK) | bruton's tyrosine kinase |
| Caplyta | ^*^ | Lumateperone | 2019 | unknown - maybe 5-HT2A receptor/central dopamine D2 receptor | (dopamine receptor) OR serotonin receptor |
| Caprelsa | ^*^ | Vandetanib | 2011 | Protein-tyrosine kinase 6; Vascular endothelial growth factor A; Angiopoietin-1 receptor; Epidermal growth factor receptor | vascular endothelial growth factor receptor-2 [MeSH Terms] |
| Carbaglu |  | Carglumic acid | 2010 | Carbamoyl-phosphate synthase [ammonia], mitochondrial | carbamoyl-phosphate synthase |
| Cerdelga | ^*,**,***^ | Eliglustat | 2014 | Ceramide glucosyltransferase | Ceramide glucosyltransferase OR glucosylceramide synthase |
| Cholbam |  | Cholic acid | 2015 | Cytochrome c oxidase subunit 5A, mitochondrial; Alcohol dehydrogenase 1C; Estrogen-related receptor gamma; Gastrotropin; Cytochrome c oxidase subunit 4 isoform 1, mitochondrial; Cytochrome c oxidase subunit 1; Cytochrome c oxidase subunit 2; Cytochrome c oxidase subunit 3; Cytochrome c oxidase subunit 5B, mitochondrial; Cytochrome c oxidase subunit 6C; Cytochrome c oxidase subunit 7B, mitochondrial; Cytochrome c oxidase subunit 7C, mitochondrial; Cytochrome c oxidase subunit 8A, mitochondrial; Cytochrome c oxidase subunit 6A2, mitochondrial; Cytochrome c oxidase subunit 6B1; Cytochrome c oxidase subunit 7A1, mitochondrial; Phospholipase A2; Liver carboxylesterase 1; Ferrochelatase; Choloylglycine hydrolase; Ferrochelatase, mitochondrial | cholic acid [MeSH Terms] |
| Cinqair | ^*^ | Reslizumab | 2016 | interleukin 5 colony-stimulating factor, eosinophil | Interleukin 5 |
| Cometriq | ^*^ | Cabozantinib | 2012 | Hepatocyte growth factor receptor; Proto-oncogene tyrosine-protein kinase receptor Ret; Vascular endothelial growth factor receptor 2 | c-Met or hepatocyte growth factor |
| Copiktra | ^*^ | Duvelisib | 2018 | Phosphatidylinositol 4,5-bisphosphate 3-kinase catalytic subunit gamma isoform, Phosphatidylinositol 4,5-bisphosphate 3-kinase catalytic subunit delta isoform | Phosphoinositide 3-kinase |
| Corlanor | ^*^ | Ivabradine | 2015 | hyperpolarization activated cyclic nucleotide-gated potassium channel | hyperpolarization-activated cyclic nucleotide-gated channels |
| Cosentyx | ^*^ | Secukinumab | 2015 | Interleukin-17A | Interleukin 17 |
| Cotellic | ^*^ | Cobimetinib | 2015 | Dual specificity mitogen-activated protein kinase kinase 1 | mitogen-activated protein kinase kinases [MeSH Terms] |
| Cyramza | ^*,**,***^ | Ramucirumab | 2014 | Vascular endothelial growth factor receptor 2 | vascular endothelial growth factor receptor-2 [MeSH Terms] |
| Daklinza | ^*^ | Daclatasvir | 2015 | Nonstructural Protein 5A NS5A | hcv ns5a |
| Daliresp | ^*^ | Roflumilast | 2011 | phosphodiesterase 4 (PDE4) | phosphodiesterase 4 |
| Dalvance | ^*^ | Dalbavancin | 2014 | n/a | PHENOTYPIC-NO SEARCH TERM |
| Darzalex | ^*^ | Daratumumab | 2015 | ADP-ribosyl cyclase/cyclic ADP-ribose hydrolase 1 | CD38 |
| Daurismo | ^*^ | Glasdegib | 2018 | n/a | hedgehog signaling OR (hedgehog AND Drosophila) |
| Dayvigo | ^*^ | Lemborexant | 2019 | orexin 1 receptor, orexin 2 receptor | orexin receptor OR hypocretin receptor |
| Defitelio |  | Defibrotide sodium | 2016 | n/a | PHENOTYPIC-NO SEARCH TERM |
| Diacomit |  | Stiripentol | 2018 | GABA-A receptor | GABA-A receptor |
| Doptelet | ^*^ | Avatrombopag | 2018 | Thrombopoietin receptor | Thrombopoietin receptor |
| Duavee | ^*^ | Bazedoxifene acetate plus oestrogens | 2013 | n/a | estrogen receptor |
| Dupixent | ^*^ | Dupilumab | 2017 | Interleukin 4 receptor and interleukin 13 receptor | interleukin 4 receptor |
| Edarbi | ^*^ | Azilsartan | 2011 | Type-1 angiotensin II receptor | angiotensin ii type 1 receptor |
| Edurant | ^*^ | Rilpivirine | 2011 | Reverse transcriptase/RNaseH; Nuclear receptor subfamily 1 group I member 2 | HIV reverse transcriptase |
| Egrifta | ^*^ | Tesamorelin | 2010 | Growth hormone-releasing hormone receptor | growth hormone-releasing hormone [MeSH Terms] |
| Elelyso | ^*^ | Taliglucerase alfa | 2012 | Glucocerebroside | beta glucocerebrosidase |
| Eliquis | ^*^ | Apixaban | 2012 | Coagulation factor X | factor Xa |
| Ella | ^*,**,***^ | Ulipristal | 2010 | Androgen receptor; Progesterone receptor; Glucocorticoid receptor | progesterone receptor [MeSH Terms] |
| Emflaza |  | Deflazacort | 2017 | Glucocorticoid receptor | glucocorticoid receptor OR NR3C1 |
| Empliciti | ^*^ | Elotuzumab | 2015 | SLAM family member 7 | slamf7 |
| Entresto | ^*^ | Sacubitril, valsartan | 2015 | membrane metallo-endopeptidase; angiotensin II receptor, type 1 | Neutral Endopeptidase |
| Entyvio | ^*^ | Vedolizumab | 2014 | Integrin beta-7; Integrin alpha-4 | alpha4beta7 integrin |
| Epclusa | ^*^ | Sofosbuvir plus velpatasvir | 2016 | n/a | hcv ns5a |
| Epidiolex | ^*^ | cannabidiol | 2018 | 28 'targets', Cannabinoid receptors not thought to be involved in anticonvulsant effects (FDA approved indication) | PHENOTYPIC-NO SEARCH TERM |
| Erivedge | ^*^ | Vismodegib | 2012 | Smoothened homolog | hedgehog signaling OR (hedgehog AND Drosophila) |
| Erleada | ^*,**,***^ | Apalutamide | 2018 | Androgen receptor | androgen receptor |
| Erwinaze | ^*^ | Asparaginase Erwinia chrysanthemi | 2011 | L-asparaginase | Asparaginase [MeSH Terms] |
| Esbriet | ^*^ | Pirfenidone | 2014 | tumor necrosis factor receptor superfamily, member 1A; mitogen-activated protein kinase 14 | PHENOTYPIC-NO SEARCH TERM |
| Eucrisa | ^*^ | Crisaborole | 2016 | cAMP-specific 3',5'-cyclic phosphodiesterase 4A; cAMP-specific 3',5'-cyclic phosphodiesterase 4B; cAMP-specific 3',5'-cyclic phosphodiesterase 4C; cAMP-specific 3',5'-cyclic phosphodiesterase 4D | phosphodiesterase 4 |
| Exondys 51 | ^*,**,***^ | Eteplirsen | 2016 | n/a | dystrophin |
| Eylea | ^*,**,***^ | Aflibercept | 2011 | Placenta growth factor; Vascular endothelial growth factor A; Vascular endothelial growth factor B | receptors, vascular endothelial growth factor [MeSH Terms] |
| Farxiga | ^*^ | Dapagliflozin | 2014 | Sodium/glucose cotransporter 2 | sodium glucose transporter |
| Farydak | ^*^ | Panobinostat | 2015 | Histone deacetylase 1; Histone deacetylase 2; Histone deacetylase 9; Histone deacetylase 10; Histone deacetylase 11; Histone deacetylase 8; Histone deacetylase 7; Histone deacetylase 3; Histone deacetylase 4; Histone deacetylase 6; Histone deacetylase 5 | histone deacetylases [MeSH Terms] |
| Fasenra | ^*^ | Benralizumab | 2017 | Interleukin 5 receptor | interleukin 5 receptor |
| Ferriprox | ^*^ | Deferiprone | 2011 | iron chelator | PHENOTYPIC-NO SEARCH TERM |
| Fetzima | ^*^ | levomilnacipran | 2013 | solute carrier family 6 neurotransmitter transporter, member 4/2 | (serotonin reuptake transporter) OR bace1 |
| Firazyr | ^*^ | Icatibant | 2011 | Aminopeptidase N; B2 bradykinin receptor | bradykinin receptor B2 |
| Firdapse |  | Amifampridine | 2018 | n/a | potassium channel |
| Fulyzaq | ^*^ | Crofelemer | 2012 | Cystic fibrosis transmembrane conductance regulator; Anoctamin-1 | "chloride channels" [MeSH Terms] |
| Fycompa | ^*^ | Perampanel | 2012 | Glutamate receptor 1 | AMPA 1 |
| Galafold | ^*^ | Migalastat | 2018 | Alpha-galactosidase A | alpha-galactosidase |
| Gattex | ^*^ | Teduglutide | 2012 | Glucagon-like peptide 2 receptor | (glucagon-like peptide 2 or glp2 or glp-2) |
| Gazyva | ^*^ | Obinutuzumab | 2013 | B-lymphocyte antigen CD20 | CD20 |
| Genvoya | ^*,**,***^ | Elvitegravir, cobicistat, emtricitabine, tenofovir | 2015 | gag-pol, HIV-1 | HIV integrase or CYP3A |
| Giapreza | ^*^ | Angiotensin II, LFPC-501 | 2017 | Type-1 angiotensin II receptor | angiotensin ii type 1 receptor |
| Gilenya | ^*^ | Fingolimod | 2010 | Sphingosine 1-phosphate receptor 5 | receptors, lysosphingolipid [MeSH Terms] |
| Gilotrif | ^*^ | Afatinib | 2013 | Epidermal growth factor receptor; Receptor tyrosine-protein kinase erbB-2; Receptor tyrosine-protein kinase erbB-4 | erbb1 OR erbb2 |
| Givlaari | ^*^ | Givosiran sodium | 2019 | aminolevulinate synthase 1 (ALAS1) mRNA | aminolevulinate synthase |
| Halaven | ^*^ | Eribulin | 2010 | Tubulin | microtubule assembly |
| Harvoni | ^*^ | Ledipasvir plus sofosbuvir | 2014 | Nonstructural protein 5A | hcv ns5a |
| Hetlioz | ^*^ | Tasimelteon | 2014 | Melatonin receptor type 1B; Melatonin receptor type 1A | melatonin receptor |
| Horizant | ^*^ | Gabapentin enacarbil | 2011 | Voltage-gated calcium channel | voltage gated calcium channel |
| Ibrance | ^*^ | Palbociclib | 2015 | Cyclin-dependent kinase 6; Cyclin-dependent kinase 4 | CDK4 or CDK6 |
| Ibsrela | ^*^ | Tenapanor | 2019 | sodium/hydrogen exchanger 3 (NHE3) | sodium hydrogen exchanger |
| Iclusig | ^*^ | Ponatinib | 2012 | Tyrosine-protein kinase Lck; Tyrosine-protein kinase ABL1; Breakpoint cluster region protein; Mast/stem cell growth factor receptor Kit; Proto-oncogene tyrosine-protein kinase receptor Ret; Angiopoietin-1 receptor; Receptor-type tyrosine-protein kinase FLT3; Fibroblast growth factor receptor 1; Fibroblast growth factor receptor 2; Fibroblast growth factor receptor 3; Fibroblast growth factor receptor 4; Proto-oncogene tyrosine-protein kinase Src; Tyrosine-protein kinase Lyn; Vascular endothelial growth factor receptor 2; Platelet-derived growth factor receptor alpha | bcr-abl |
| Idelvion | ^*^ | Albutrepenonacog alfa | 2016 | Coagulation Factor IX | coagulation factor IX |
| Idhifa | ^*^ | Enasidenib mesylate | 2017 | isocitrate dehydrogenase-2 (IDH2) | isocitrate dehydrogenase |
| Imbruvica | ^*^ | Ibrutinib | 2013 | Tyrosine-protein kinase BTK | bruton's tyrosine kinase |
| Imfinzi | ^*^ | Durvalumab | 2017 | Programmed cell death ligand 1 | programmed cell death-ligand 1 OR PDL1 |
| Imlygic | ^*^ | Talimogene laherparepvec | 2015 | Granulocyte-macrophage colony-stimulating factor | Granulocyte-macrophage colony-stimulating factor |
| Impavido |  | Miltefosine | 2014 | n/a | PHENOTYPIC-NO SEARCH TERM |
| Incivek | ^*^ | Telaprevir | 2011 | Genome polyprotein | HCV NS3 |
| Ingrezza | ^*^ | Valbenazine tosylate | 2017 | Synaptic vesicle monoamine transporter 2 (VMAT2) | monoamine transporter |
| Inlyta | ^*^ | Axitinib | 2012 | Vascular endothelial growth factor receptor 3; Vascular endothelial growth factor receptor 2; Vascular endothelial growth factor receptor 1 | receptors, vascular endothelial growth factor [MeSH Terms] |
| Inrebic | ^*^ | Fedratinib hydrochloride | 2019 | Janus Associated Kinase 2 (JAK2) and FMS-like tyrosine kinase 3 (FLT3) | receptor tyrosine kinase |
| Invokana | ^*^ | Canagliflozin | 2013 | Sodium/glucose cotransporter 2 | sodium glucose transporter |
| Ixinity | ^*^ | Recombinant Coagulation factor IX | 2015 | coagulation factor IX | coagulation factor IX |
| Jakafi | ^*^ | Ruxolitinib | 2011 | Tyrosine-protein kinase JAK2; Tyrosine-protein kinase JAK1 | janus kinases [MeSH Terms] |
| Jardiance | ^*^ | Empagliflozin | 2014 | Sodium/glucose cotransporter 2 | sodium glucose transporter |
| Jetrea | ^*,**,***^ | Ocriplasmin | 2012 | Fibronectin; Alpha-2-macroglobulin; Alpha-2-antiplasmin | plasmin |
| Jevtana | ^*^ | Cabazitaxel | 2010 | Tubulin beta-1 chain | microtubule assembly |
| Juxtapid | ^*^ | Lomitapide | 2012 | Microsomal triglyceride transfer protein large subunit | Microsomal triglyceride transfer protein |
| Kadcyla | ^*^ | Ado-trastuzumab emtansine | 2013 | Receptor tyrosine-protein kinase erbB-2 | HER2 OR eErb2 OR p185 |
| Kalydeco | ^*^ | Ivacaftor | 2012 | cystic fibrosis transmembrane conductance regulator (CFTR) protein, homozygous F508del-CFTR gene | CFTR |
| Kanuma | ^*^ | Sebelipase alfa | 2015 | lipase A, lysosomal acid, cholesterol esterase | lysosomal acid lipase |
| Kengreal | ^*^ | Cangrelor | 2015 | P2Y purinoceptor 12 | p2y receptor |
| Kevzara | ^*^ | Sarilumab | 2017 | Interleukin 6 receptor | interleukin 6 receptor |
| Keytruda | ^*,**,***^ | Pembrolizumab | 2014 | Programmed cell death protein 1 | "programmed cell death 1" |
| Kisqali | ^*^ | Ribociclib succinate | 2017 | cyclin dependent protein kinase (CDK) 4 & 6 | "cyclin-dependent kinase" |
| Kovaltry | ^*^ | Recombinant Antihemophilic Factor VIII (Human) | 2016 | congenital Factor VIII | coagulation "factor VIII" |
| Krintafel |  | Tafenoquine | 2018 | n/a | PHENOTYPIC-NO SEARCH TERM |
| Krystexxa | ^*^ | Pegloticase | 2010 | Uric acid | urate oxidase [MeSH Terms] |
| Kybella | ^*^ | Deoxycholic acid | 2015 | n/a | PHENOTYPIC-NO SEARCH TERM |
| Kynamro | ^*^ | Mipomersen | 2013 | Apo-B 100 mRNA | APOB OR apolipoprotein B |
| Kyprolis | ^*^ | Carfilzomib | 2012 | Proteasome subunit beta type-9; Proteasome subunit beta type-8; Proteasome subunit beta type-5; Proteasome subunit beta type-10; Proteasome subunit beta type-2; Proteasome subunit beta type-1 | proteasome endopeptidase complex [MeSH Terms] |
| Lartruvo | ^*^ | Olaratumab | 2016 | Platelet-derived growth factor receptor alpha | receptors, platelet-derived growth factor [MeSH Terms] |
| Lastacaft | ^*^ | Alcaftadine | 2010 | Histamine H1 receptor | H1 histamine receptor [MeSH Terms] |
| Latuda | ^*^ | Lurasidone | 2010 | dopamine receptor D2; 5-hydroxytryptamine serotonin receptor 7, adenylate cyclase-coupled; 5-hydroxytryptamine serotonin receptor 1A, G-protein-coupled; 5-hydroxytryptamine serotonin receptor 1A, G-protein-coupled | (dopamine receptor) OR serotonin receptor |
| Lenvima | ^*^ | Lenvatinib | 2015 | Mast/stem cell growth factor receptor Kit; Fibroblast growth factor receptor 4; Fibroblast growth factor receptor 3; Fibroblast growth factor receptor 2; Fibroblast growth factor receptor 1; Vascular endothelial growth factor receptor 3; Vascular endothelial growth factor receptor 2; Vascular endothelial growth factor receptor 1 | receptor tyrosine kinase |
| Linzess | ^*^ | Linaclotide | 2012 | Heat-stable enterotoxin receptor | guanylyl cyclase c |
| Lonsurf | ^*^ | Tipiracil plus trifluridine | 2015 | thymidine phosphorylase | thymidine phosphorylase |
| Lorbrena | ^*^ | Lorlatinib | 2018 | n/a | ALK tyrosine kinase receptor |
| Lucemyra |  | Lofexidine | 2018 | alpha-2 adrenergic receptor | alpha-2-adrenergic receptor |
| Lumizyme | ^*^ | Alglucosidase alfa | 2010 | Cation-dependent mannose-6-phosphate receptor | alpha glucosidase FAILED TIME |
| Lynparza | ^*^ | Olaparib | 2014 | Poly [ADP-ribose] polymerase 3; Poly [ADP-ribose] polymerase 2; Poly [ADP-ribose] polymerase 1 | PARP |
| Mavyret | ^*^ | Glecaprevir, pibrentasvir | 2017 | glecaprevir: HCV NS3/4A protease AND pibrentasvir: HCV Nonstructural Protein 5A (NS5A) | NS3 protease OR Nonstructural protein 5A |
| Mayzent | ^*^ | Siponimod | 2019 | sphingosine-1-phosphate (S1P) receptor | sphingosine 1-phosphate |
| Mekinist | ^*^ | Trametinib | 2013 | Dual specificity mitogen-activated protein kinase kinase 1; Dual specificity mitogen-activated protein kinase kinase 2 | mitogen-activated protein kinase kinases [MeSH Terms] |
| Mektovi | ^*^ | Binimetinib | 2018 | Mitogen-activated protein kinase kinase 1 | mitogen-activated protein kinase kinases [MeSH Terms] |
| Motegrity |  | Prucalopride | 2018 | 5-hydroxytryptamine receptor 4 | 5-HT4 |
| Movantik | ^*^ | Naloxegol | 2014 | Mu-type opioid receptor | receptors, opioid, mu [MeSH Terms] |
| Moxidectin |  | Moxidectin | 2018 | chloride channel | "chloride channels" [MeSH Terms] |
| Mulpleta | ^*^ | Lusutrombopag | 2018 | Thrombopoietin receptor | Thrombopoietin receptor |
| Myalept | ^*^ | Metreleptin | 2014 | Leptin receptor | Leptin |
| Myrbetriq | ^*^ | Mirabegron | 2012 | Beta-3 adrenergic receptor | "beta 3" AND adrenergic receptor |
| Natazia | ^*^ | Oestradiol valerate, dienogest | 2010 | Estrogen receptor | estrogen receptor |
| Natpara | ^*,**,***^ | Parathyroid hormone | 2015 | parathyroid hormone 1 receptor | parathyroid hormone receptor |
| Natroba | ^*^ | Spinosad | 2011 | Nicotinic acetylcholine receptor | receptors, cholinergic [MeSH Terms] |
| Nerlynx | ^*^ | Neratinib maleate | 2017 | human epidermal growth factor (HER) 2 and 4 | Epidermal growth factor receptor |
| Nesina | ^*,**^ | Alogliptin | 2013 | Dipeptidyl peptidase 4 | dipeptidyl-peptidase 4 |
| Ninlaro | ^*^ | Ixazomib | 2015 | nuclear factor of kappa light polypeptide gene enhancer in B-cells 1 | nf-kappa b |
| Northera |  | Droxidopa | 2014 | Beta-3 adrenergic receptor; Alpha-2A adrenergic receptor; Alpha-2C adrenergic receptor; Beta-2 adrenergic receptor; Alpha-1B adrenergic receptor; Alpha-1A adrenergic receptor; Phenylalanine-4-hydroxylase; Beta-1 adrenergic receptor; Alpha-2B adrenergic receptor; Alpha-1D adrenergic receptor | adrenergic receptor |
| Nourianz | ^*^ | Istradefylline | 2019 | Adenosine A2A receptor | "adenosine receptor" |
| Novoeight | ^*^ | Turoctocog alfa | 2013 | Coagulation factor VIII replacement | coagulation "factor VIII" |
| Nubeqa | ^*^ | Darolutamide | 2019 | Androgen receptor | androgen receptor |
| Nucala | ^*,**,***^ | Mepolizumab | 2015 | Interleukin-5 | Interleukin 5 |
| Nulojix | ^*^ | Belatacept | 2011 | CD80, CD86 | CD80 OR CD86 |
| Nuplazid | ^*^ | Pimavanserin | 2016 | 5-hydroxytryptamine serotonin receptor 2A, G-protein-coupled | serotonin receptor 2a |
| Nuwiq | ^*^ | Simoctocog alfa | 2015 | coagulation factor VIII | coagulation "factor VIII" |
| Ocaliva | ^*^ | Obeticholic acid | 2016 | nuclear receptor subfamily 1, group H, member 4 | farnesoid x receptor |
| Ocrevus | ^*,**,***^ | Ocrelizumab | 2017 | CD20 | CD20 |
| Odomzo | ^*^ | Sonidegib | 2015 | Smoothened homolog | smoothened |
| Ofev | ^*^ | Nintedanib | 2014 | Fibroblast growth factor receptor 2; Vascular endothelial growth factor receptor 3; Vascular endothelial growth factor receptor 2; Vascular endothelial growth factor receptor 1; Tyrosine-protein kinase Lck; Receptor-type tyrosine-protein kinase FLT3; Fibroblast growth factor receptor 3; Proto-oncogene tyrosine-protein kinase Src; Tyrosine-protein kinase Lyn; Fibroblast growth factor receptor 1 | receptor tyrosine kinase |
| Olumiant | ^*^ | Baricitinib | 2018 | Tyrosine-protein kinase JAK1, Tyrosine-protein kinase JAK2, Tyrosine-protein kinase JAK3, Protein-tyrosine kinase 2-beta | janus kinases [MeSH Terms] |
| Olysio | ^*^ | Simeprevir | 2013 | NS3 protease | HCV NS3 |
| Omontys | ^*^ | Peginesatide | 2012 | Erythropoietin receptor | erythropoietin |
| Onfi |  | Clobazam | 2011 | alpha(1)-GABA(A) | GABA-A receptor |
| Onpattro | ^*^ | Patisiran | 2018 | Transthyretin mRNA | Transthyretin |
| Opdivo | ^*,**,***^ | Nivolumab | 2014 | Programmed cell death protein 1 | "programmed cell death 1" |
| Opsumit | ^*^ | Macitentan | 2013 | Endothelin B receptor; Endothelin-1 receptor | endothelin receptor |
| Orilissa | ^*,**,***^ | Elagolix sodium | 2018 | n/a | Gonadotropin-releasing hormone receptor |
| Orkambi | ^*^ | Ivacaftor plus lumacaftor | 2015 | cystic fibrosis transmembrane conductance regulator ATP-binding cassette subfamily C, member 7 | CFTR |
| Osphena | ^*^ | Ospemifene | 2013 | Estrogen receptor | estrogen receptor |
| Otezla | ^*^ | Apremilast | 2014 | cAMP-specific 3',5'-cyclic phosphodiesterase 4D; cAMP-specific 3',5'-cyclic phosphodiesterase 4B; Nitric oxide synthase, endothelial; Interferon gamma; Interleukin-2; Tumor necrosis factor; cAMP-specific 3',5'-cyclic phosphodiesterase 4A | phosphodiesterase 4 |
| Oxbryta | ^*^ | Voxelotor | 2019 | hemoglobin S (HbS) | hemoglobin S |
| Ozempic | ^*^ | Semaglutide | 2017 | Glucagon-like peptide 1 receptor | glucagon-like peptide 1 |
| Parsabiv | ^*^ | Etelcalcetide | 2017 | calcium-sensing receptor | "calcium sensing receptor" |
| Perjeta | ^*,**,***^ | Pertuzumab | 2012 | Receptor tyrosine-protein kinase erbB-2 | HER2 OR eErb2 OR p185 |
| Picato | ^*^ | Ingenol mebutate | 2012 | Protein kinase C alpha type, Protein kinase C delta type | protein kinase c-delta [MeSH Terms] |
| Pifeltro | ^*^ | Doravirine | 2018 | Reverse transcriptase/RNaseH p66 Subunit | HIV reverse transcriptase |
| Piqray | ^*^ | Alpelisib | 2019 | phosphatidylinositol-3-kinase (predominant inhibition against PI3Kα) | Phosphoinositide 3-kinase |
| Plegridy | ^*^ | Peginterferon beta-1A | 2014 | Interferon alpha/beta receptor | interferon beta 1 |
| Polivy | ^*^ | Polatuzumab vedotin-PIIQ | 2019 | CD79b (antibody-drug (MMAE) conjugate) | CD79b |
| Pomalyst | ^*^ | Pomalidomide | 2013 | Tumor necrosis factor; Protein cereblon; Prostaglandin G/H synthase 2 | PHENOTYPIC-NO SEARCH TERM |
| Portrazza | ^*^ | Necitumumab | 2015 | Epidermal growth factor receptor | EGFR |
| Potiga |  | Ezogabine | 2011 | Potassium voltage-gated channel subfamily KQT member 5; Potassium voltage-gated channel subfamily KQT member 4; Potassium voltage-gated channel subfamily KQT member 3; Potassium voltage-gated channel subfamily KQT member 2 | potassium voltage-gated channel |
| Pradaxa | ^*^ | Dabigatran | 2010 | Prothrombin | Thrombin [MeSH Terms] |
| Praluent | ^*^ | Alirocumab | 2015 | Proprotein convertase subtilisin/kexin type 9 | proprotein convertase subtilisin kexin type 9 |
| Praxbind | ^*^ | Idarucizumab | 2015 | n/a | Dabigatran |
| Prepopik | ^*^ | Picosulfate | 2012 | n/a | PHENOTYPIC-NO SEARCH TERM |
| Pretomanid |  | Pretomanid | 2019 | mycolic acid biosynthesis (detail not known) | PHENOTYPIC-NO SEARCH TERM |
| Prevymis | ^*^ | Letermovir | 2017 | Human cytomegalovirus terminase subunit UL56 | PHENOTYPIC-NO SEARCH TERM |
| Prolia | ^*,**,***^ | Denosumab | 2010 | Tumor necrosis factor ligand superfamily member 11 | tnfsf11 |
| Provenge | ^*,**,***^ | Sipuleucel-T | 2010 | Prostatic acid phosphatase | Prostatic acid phosphatase |
| Radicava | ^*^ | Edaravone | 2017 | unknown (is free radical scavenger oxstress hypothesized in ALS neurodegenration) | PHENOTYPIC-NO SEARCH TERM |
| Rapivab | ^*^ | Peramivir | 2014 | Neuraminidase | viral neuraminidase |
| Repatha | ^*^ | Evolocumab | 2015 | proprotein convertase subtilisin/kexin type 9 | proprotein convertase subtilisin kexin type 9 |
| Rexulti | ^*^ | Brexpiprazole | 2015 | dopamine receptor D2; dopamine receptor D3; 5-hydroxytryptamine serotonin receptor 1A, G-protein-coupled;5-hydroxytryptamine serotonin receptor 2A, G-protein-coupled | (dopamine receptor) OR serotonin receptor |
| Reyvow | ^*^ | Lasmiditan succinate | 2019 | serotonin (5-HT) 1F receptor | 5-HT1 receptor |
| Rhopressa | ^*^ | Netarsudil | 2017 | Rho-associated protein kinase | Rho-associated protein kinase |
| Rinvoq | ^*^ | Upadacitinib | 2019 | Janus kinase (JAK) 1 | janus kinases [MeSH Terms] |
| Rixubis | ^*^ | Coagulation Factor IX (Recombinant) | 2013 | coagulation factor IX replacement | coagulation factor IX |
| Rozlytrek | ^*^ | Entrectinib | 2019 | tropomyosin receptor tyrosine kinase (TRK)-A, B, C, Proto-oncogene tyrosine-protein kinase ROS1 (ROS1), Anaplastic lymphoma kinase (ALK) | receptor tyrosine kinase |
| Rubraca | ^*^ | Rucaparib | 2016 | ADP-ribosyltransferase NAD+, poly ADP-ribose polymerase; poly ADP-ribose polymerase 2 | poly adp ribose polymerase |
| Ruconest | ^*^ | Conestat alfa | 2014 | C1 esterase | C1 esterase |
| Rydapt | ^*^ | Midostaurin | 2017 | multiple receptor tyrosine kinases | receptor tyrosine kinase |
| Savaysa | ^*^ | Edoxaban | 2015 | Coagulation factor X | coagulation factor X |
| Scenesse | ^*^ | Afamelanotide | 2019 | melanocortin receptor 1 (MC1-R) | melanocortin receptor |
| Seysara | ^*^ | Sarecycline | 2018 | n/a | PHENOTYPIC-NO SEARCH TERM |
| Signifor | ^*^ | Pasireotide | 2012 | Somatostatin receptor 1, Somatostatin receptor 2, Somatostatin receptor 3, Somatostatin receptor 5 | somatostatin |
| Siliq | ^*^ | Brodalumab | 2017 | Interleukin 17 receptor | interleukin 17 receptor |
| Sirturo | ^*^ | Bedaquiline | 2012 | ATP synthase C chain AtpE lipid-binding protein dicyclohexylcarbodiimide-binding protein | Escherichia coli ATP synthase |
| Sovaldi | ^*^ | Sofosbuvir | 2013 | Hepatitis C virus NS5B RNA-dependent RNA polymerase | HCV NS5B |
| Spinraza | ^*,**,***^ | Nusinersen | 2016 | survival of motor neuron 2, centromeric | ([(survival motor neuron protein) OR smn] OR smn1 smn2) OR survival of motor neuron 2 protein [MeSH Terms] |
| Steglatro | ^*^ | Ertugliflozin | 2017 | Sodium/glucose cotransporter 2 (SGLT2) | sodium glucose transporter |
| Stendra | ^*^ | Avanafil | 2012 | cGMP-specific 3',5'-cyclic phosphodiesterase | phosphodiesterase 5 |
| Stivarga | ^*^ | Regorafenib | 2012 | Mast/stem cell growth factor receptor Kit; Platelet-derived growth factor receptor beta; Proto-oncogene tyrosine-protein kinase receptor Ret; Tyrosine-protein kinase ABL1; Tyrosine-protein kinase FRK; Mitogen-activated protein kinase 11; Vascular endothelial growth factor receptor 1; Vascular endothelial growth factor receptor 2; Vascular endothelial growth factor receptor 3; Serine/threonine-protein kinase B-raf; RAF proto-oncogene serine/threonine-protein kinase; Ephrin type-A receptor 2; High affinity nerve growth factor receptor; Discoidin domain-containing receptor 2; Angiopoietin-1 receptor; Fibroblast growth factor receptor 2; Fibroblast growth factor receptor 1; Platelet-derived growth factor receptor alpha | urea AND kinase inhibitor |
| Strensiq | ^*^ | Asfotase alfa | 2015 | Sphingosine 1-phosphate receptor 1 | tissue nonspecific alkaline phosphatase |
| Striverdi Respimat | ^*^ | Olodaterol | 2014 | Beta-2 adrenergic receptor | beta-2-adrenergic receptor |
| Sunosi | ^*^ | Solriamfetol | 2019 | Dopamine transporter OR Norepinephrine transporter | (noradrenaline reuptake) OR (dopamine reuptake) |
| Surfaxin | ^*,**,***^ | Lucinactant | 2012 | n/a | surfactant protein B |
| Sylvant | ^*^ | Siltuximab | 2014 | Interleukin-6 | interleukin 6 receptor |
| Symdeko | ^*^ | Tezacaftor and ivacaftor | 2018 | cystic fibrosis transmembrane conductance regulator (CFTR) protein | CFTR |
| Symproic | ^*^ | Naldemedine | 2017 | mu-, delta-, and kappa-opioid receptors | receptors, opioid [MeSH Terms] |
| Tafinlar | ^*^ | Dabrafenib | 2013 | RAF proto-oncogene serine/threonine-protein kinase; Serine/threonine-protein kinase B-raf; LIM domain kinase 1; Serine/threonine-protein kinase Nek11; Serine/threonine-protein kinase SIK1 | braf |
| Tagrisso | ^*^ | Osimertinib | 2015 | Epidermal growth factor receptor | EGFR |
| Taltz | ^*^ | Ixekizumab | 2016 | Interleukin-17A | Interleukin 17 |
| Talzenna | ^*^ | Talazoparib | 2018 | Poly [ADP-ribose] polymerase 1, Poly [ADP-ribose] polymerase 2 | PARP |
| Tanzeum | ^*,**^ | Albiglutide | 2014 | Glucagon-like peptide 1 receptor | glucagon-like peptide 1 |
| Tavalisse | ^*^ | Fostamatinib | 2018 | spleen Tyrosine-protein kinase | spleen tyrosine kinase |
| Tecentriq | ^*,**^ | Atezolizumab | 2016 | Programmed cell death 1 ligand 1 | programmed cell death-ligand 1 OR PDL1 |
| Tecfidera | ^*^ | Dimethyl fumarate | 2013 | Kelch-like ECH-associated protein 1 | nrf2 OR NFE2l2 |
| Tegsedi | ^*^ | Inotersen | 2018 | Transthyretin mRNA | Transthyretin |
| Tibsovo | ^*^ | Ivosidenib | 2018 | n/a | isocitrate dehydrogenase |
| Tivicay | ^*^ | Dolutegravir | 2013 | Integrase | HIV integrase |
| TPOXX | ^*,**,***^ | Tecovirimat | 2018 | orthopoxvirus VP37 protein | "orthopoxvirus envelope" |
| Tradjenta | ^*,**^ | Linagliptin | 2011 | Dipeptidyl peptidase 4 | dipeptidyl-peptidase 4 |
| Tremfya | ^*^ | Guselkumab | 2017 | n/a | interleukin 23 |
| Tresiba | ^*^ | Insulin degludec | 2015 | insulin receptor | insulin receptor |
| Trikafta | ^*^ | Elexacaftor, ivacaftor, tezacaftor | 2019 | cystic fibrosis transmembrane conductance regulator (CFTR) protein, homo- and heterozygous F508del-CFTR gene | CFTR |
| Trulance | ^*^ | Plecanatide | 2017 | Guanylyl cyclase C; guanylate | guanylyl cyclase c |
| Trulicity | ^*^ | Dulaglutide | 2014 | Glucagon-like peptide 1 receptor | glucagon-like peptide 1 |
| Tudorza Pressair | ^*^ | Aclidinium bromide | 2012 | Muscarinic acetylcholine receptor M1, Muscarinic acetylcholine receptor M2, Muscarinic acetylcholine receptor M3, Muscarinic acetylcholine receptor M4, Muscarinic acetylcholine receptor M5 | M3 muscarinic receptor |
| Turalio | ^*^ | Pexidartinib hydrochloride | 2019 | cd117=cKIT, cd115=CSF1R | cd117 OR cd115 |
| Tymlos | ^*^ | Abaloparatide | 2017 | Parathyroid hormone 1 receptor (PTH1R) | parathyroid hormone receptor |
| Ubrelvy | ^*^ | Ubrogepant | 2019 | calcitonin gene-related peptide receptor | calcitonin gene-related peptide receptor |
| Ultomiris | ^*^ | Ravulizumab | 2018 | n/a | "complement C5 protein" |
| Unituxin | ^*^ | Dinutuximab | 2015 | n/a | (glycolipid gd2) OR disialoganglioside gd2 |
| Uptravi | ^*^ | Selexipag | 2015 | Prostacyclin receptor | receptors, epoprostenol [MeSH Terms] |
| Varubi | ^*^ | Rolapitant | 2015 | tachykinin receptor 1 | (substance p receptor) OR tachykinin receptor 1 |
| Vascepa | ^*^ | Ethyl eicosapentaenoic acid | 2012 | n/a | PHENOTYPIC-NO SEARCH TERM |
| Veltassa | ^*^ | Patiromer | 2015 | n/a | PHENOTYPIC-NO SEARCH TERM |
| Venclexta | ^*^ | Venetoclax | 2016 | Apoptosis regulator Bcl-2 | Bcl-2 |
| Verzenio | ^*^ | Abemaciclib | 2017 | Cyclin-dependent kinase 4 and 6 | "cyclin-dependent kinase" |
| Viberzi | ^*^ | Eluxadoline | 2015 | Mu-type opioid receptor; Kappa-type opioid receptor; Delta-type opioid receptor | receptors, opioid [MeSH Terms] |
| Victoza | ^*^ | Liraglutide | 2010 | Glucagon-like peptide 1 receptor | glucagon-like peptide 1 |
| Victrelis | ^*^ | Boceprevir | 2011 | Genome polyprotein | HCV NS3 |
| Viekira Pak | ^*^ | Ombitasvir, paritaprevir, dasabuvir | 2014 | Nonstructural protein 5A, Genome polyprotein, Nonstructural protein 5B NS5B | hcv ns5a OR hcv NS3 OR HCV NS5B |
| Viibryd | ^*^ | Vilazodone | 2011 | 5-hydroxytryptamine receptor 1A | 5-ht1a receptors OR serotonin reuptake |
| Vimizim | ^*^ | Elosulfase alfa | 2014 | N-acetylgalactosamine-6-sulfatase | N Acetylgalactosamine 6 sulfatase |
| Vitrakvi | ^*^ | Larotrectinib | 2018 | n/a | tropomyosin receptor kinases |
| Vizimpro | ^*^ | Dacomitinib | 2018 | Epidermal growth factor receptor | Epidermal growth factor receptor |
| Vonvendi | ^*^ | von Willebrand factor (Recombinant) | 2015 | von Willebrand factor | von willebrand factor |
| Vosevi | ^*^ | Sofosbuvir, velpatasvir, voxilaprevir | 2017 | Hepatitis C virus Serine protease NS3/4A (HCV NS3/4A) | Hepatitis C virus protease |
| Vpriv | ^*^ | Velaglucerase alfa | 2010 | Glucosylceramidase | "beta-glucosidase" [MeSH Terms] OR "beta-glucosidase" [All Fields] |
| Vraylar | ^*^ | Cariprazine | 2015 | dopamine receptor D2; dopamine receptor D3 | dopamine receptor |
| Vyleesi | ^*^ | Bremelanotide | 2019 | melanocortin receptor (MCR), nonselective, MC1R, MC4R, MC3R, MC5R, MC2R | melanocortin receptor |
| Vyndaqel | ^*,**,***^ | Tafamidis meglumine | 2019 | Transthyretin transport protein thyroxin binding site | Transthyretin |
| Vyondys 53 | ^*,**,***^ | golodirsen | 2019 | dystrophin pre-mRNA exon 53 | dystrophin |
| Vyzulta | ^*^ | Latanoprostene bunod | 2017 | Prostaglandin F2-alpha receptor | Prostaglandin F receptor |
| Wakix | ^*^ | Pitolisant hydrochloride | 2019 | histamine H3 receptor | histamine H3 receptor |
| Xadago | ^*^ | Safinamide | 2017 | monoamine oxidase [flavin-containing] B | monoamine oxidase B |
| Xalkori | ^*^ | Crizotinib | 2011 | Hepatocyte growth factor receptor; ALK tyrosine kinase receptor | anaplastic lymphoma kinase |
| Xarelto | ^*^ | Rivaroxaban | 2011 | Coagulation factor X | coagulation factor X |
| Xcopri | ^*^ | Cenobamate | 2019 | EXACT MOA UNKNOWN, reduce voltage-gated Na+ currents, γ-aminobutyric acid (GABAa) ion channel +ve allosteric modulation | voltage gated sodium channel |
| Xeljanz | ^*^ | Tofacitinib | 2012 | Tyrosine-protein kinase JAK2; Tyrosine-protein kinase JAK1; Tyrosine-protein kinase JAK3 | janus kinases [MeSH Terms] |
| Xeomin | ^*^ | IncobotulinumtoxinA | 2010 | Synaptosomal-associated protein 25 | botulinum toxin A |
| Xermelo | ^*^ | Telotristat etiprate | 2017 | Tryptophan hydroxylase | Tryptophan hydroxylase |
| Xiaflex | ^*^ | Collagenase clostridium histolyticum | 2010 | Collagen alpha-1III chain; Collagen alpha-2I chain; Collagen alpha-1II chain; Collagen alpha-1I chain; | "microbial collagenase" [MeSH Terms] |
| Xiidra | ^*^ | Lifitegrast | 2016 | integrin, alpha L antigen CD11A p180, lymphocyte function-associated antigen 1, alpha polypeptide; intercellular adhesion molecule 1 | lymphocyte function-associated antigen-1, LFA-1 |
| Xofluza | ^*^ | Baloxavir marboxil | 2018 | Influenza polymerase acidic proteins | Polymerase Acidic Endonuclease |
| Xospata | ^*^ | Gilteritinib | 2018 | Receptor-type tyrosine-protein kinase FLT3, Tyrosine-protein kinase receptor UFO, ALK tyrosine kinase receptor | receptor tyrosine kinase |
| Xpovio | ^*^ | Selinexor | 2019 | exportin-1 (XPO1) | exportin |
| Xtandi | ^*,**,***^ | Enzalutamide | 2012 | Androgen receptor | androgen receptor |
| Xuriden | ^*^ | Uridine triacetate | 2015 | U6 snRNA-associated Sm-like protein LSm6; Nucleoside-specific channel-forming protein tsx | ("fluorouracil" [MeSH Terms] OR ("capecitabine" [MeSH Terms] |
| Yervoy | ^*,**,***^ | Ipilimumab | 2011 | Cytotoxic T-lymphocyte protein 4 | cytotoxic T-lymphocyte-associated protein 4 OR CTLA-4 |
| Yondelis | ^*^ | Trabectedin | 2015 | n/a | PHENOTYPIC-NO SEARCH TERM |
| Yupelri | ^*^ | Revefenacin | 2018 | muscarinic receptors M1 to M5 | muscarinic receptor |
| Zaltrap | ^*,**^ | Ziv-aflibercept | 2012 | Placenta growth factor, Vascular endothelial growth factor A | receptors, vascular endothelial growth factor [MeSH Terms] |
| Zejula | ^*^ | Niraparib | 2017 | Poly [ADP-ribose] polymerase (PARP) | PARP |
| Zelboraf | ^*^ | Vemurafenib | 2011 | Serine/threonine-protein kinase B-raf | braf |
| Zepatier | ^*^ | Elbasvir, grazoprevir | 2016 | polyprotein, hepatitis-C virus; HCV nonstructural protein | hcv ns3 OR hcv ns4a |
| Zinbryta | ^*,**,***^ | Daclizumab | 2016 | interleukin-2 receptor | interleukin 2 receptor |
| Zinplava | ^*^ | Bezlotoxumab | 2016 | clostridium difficile toxin B neutralization | clostridium difficile toxin b |
| Zioptan | ^*^ | Tafluprost | 2012 | Prostaglandin F2-alpha receptor | Prostaglandin F receptor |
| Zontivity | ^*^ | Vorapaxar | 2014 | Proteinase-activated receptor 1 | "protease activated receptor" |
| Zulresso | ^*^ | Brexanolone | 2019 | GABA-A receptor (anion channel) | GABA-A receptor |
| Zurampic | ^*^ | Lesinurad | 2015 | Solute carrier family 22 member 12; Solute carrier family 22member 11 | urate transporter OR SLC22A12 OR URAT1 |
| Zydelig | ^*^ | Idelalisib | 2014 | PI3-kinase p110-delta subunit | Phosphoinositide 3-kinase |
| Zykadia | ^*^ | Ceritinib | 2014 | ALK tyrosine kinase receptor | anaplastic lymphoma kinase |
| Zytiga | ^*^ | Abiraterone | 2011 | Steroid 17-alpha-hydroxylase/17,20 lyase | CYP17A1 |
