# Supplementary material for: NIH funding for patents that contribute to market exclusivity of drugs approved 2010–2019 and the public interest protections of Bayh-Dole
Source: PLoS One. 2023 Jul 26;18(7):e0288447. doi: 10.1371/journal.pone.0288447 (PMC10370755; doi:10.1371/journal.pone.0288447)
Supplement: S2 Table — A. Patents in RePORT with associated publication related to drugs or drug targets (n = 104); B. Patents in RePORT but without associated publication related to drugs or drug targets (n = 15). (DOCX) [file pone.0288447.s002.docx]

| **S2 Table. NIH-funded patents associated with drugs approved 2010–2019 in DrugPatentWatch.** A. Patents in RePORT associated with NIH-funded project related to research on drugs or drug targets (n=104); B. Patents in RePORT associated with NIH-funded project not related to a drug or drug target (n=15). | | | | | |
| --- | --- | --- | --- | --- | --- |
|  | |  | |  | |
| 1. **Patents in RePORT with associated publication related to drugs or drug targets** | | | | | |
| **Patent ID** | **Patent Title** | | **Patent Organization** | |  |
| 5407914 | Pulmonary surfactant protein and related polypeptides | | SCRIPPS RESEARCH INSTITUTE | |  |
| 9175326 | Transglycosylation activity of glycosynthase mutants of an endo-beta-N-acetylglucosaminidase (endo-D) from streptococcus pneumoniae | | UNIVERSITY OF MARYLAND BALTIMORE | |  |
| 9434786 | Chemoenzymatic glycoengineering of antibodies and Fc fragments thereof | | UNIVERSITY OF MARYLAND BALTIMORE | |  |
| 9850473 | Transglycosylation activity of glycosynthase mutants of an endo-beta-N-acetylglucosaminidase (endo-D) from Streptococcus pneumoniae | | UNIVERSITY OF MARYLAND BALTIMORE | |  |
| 10314855 | Methods relating to lung cancer | | BOSTON UNIVERSITY MEDICAL CAMPUS | |  |
| 7838657 | Spinal muscular atrophy (SMA) treatment via targeting of SMN2 splice site inhibitory sequences | | UNIVERSITY OF MASSACHUSETTS MEDICAL SCH | |  |
| 8110560 | Spinal muscular atrophy (SMA) treatment via targeting of SMN2 splice site inhibitory sequences | | UNIVERSITY OF MASSACHUSETTS MEDICAL SCH | |  |
| 8361977 | Compositions and methods for modulation of SMN2 splicing | | COLD SPRING HARBOR LABORATORY | |  |
| 9730888 | Method of enhancing delivery of therapeutic compounds to the eye | | WAYNE STATE UNIVERSITY | |  |
| 10501518 | Alpha-/beta-polypeptide analogs of parathyroid hormone (PTH) and method of using same | | UNIVERSITY OF WISCONSIN-MADISON | |  |
| 4423037 | Inhibitors of peptide hormone action | | MASSACHUSETTS GENERAL HOSPITAL | |  |
| 4746508 | Drug administration | | BETH ISRAEL DEACONESS MEDICAL CENTER | |  |
| 5643746 | Human epidermal gene promoter | | UNIVERSITY OF ROCHESTER | |  |
| 5660826 | Therapeutic sepsis treatment using antagonists to PTHrP | | UNIVERSITY OF CALIFORNIA, SAN FRANCISCO | |  |
| 5763416 | Gene transfer into bone cells and tissues | | UNIVERSITY OF MICHIGAN | |  |
| 5849695 | Parathyroid hormone analogues useful for treatment of osteoporosis and disorders of calcium metabolism in mammals | | UNIVERSITY OF CALIFORNIA SYS OFFICE/PRES | |  |
| 5925549 | Soluble 7-transmembrane domain G-protein-coupled receptor compositions and methods | | STANFORD UNIVERSITY | |  |
| 5942496 | Methods and compositions for multiple gene transfer into bone cells | | UNIVERSITY OF MICHIGAN | |  |
| 5962427 | In vivo gene transfer methods for wound healing | | UNIVERSITY OF MICHIGAN | |  |
| 6440445 | Methods and compounds for treatment of abnormal uterine bleeding | | BRIGHAM AND WOMEN'S HOSPITAL | |  |
| 6503713 | Methods for identifying RNA binding compounds | | UNIV OF MED/DENT OF NJ-NJ MEDICAL SCHOOL | |  |
| 6541022 | Mineral and cellular patterning on biomaterial surfaces | | UNIVERSITY OF MICHIGAN | |  |
| 6541220 | Nucleic acid encoding PTH1R receptor | | MASSACHUSETTS GENERAL HOSPITAL | |  |
| 6767928 | Mineralization and biological modification of biomaterial surfaces | | UNIVERSITY OF MICHIGAN | |  |
| 6811776 | Process for ex vivo formation of mammalian bone and uses thereof | | UNIVERSITY OF MICHIGAN | |  |
| 6864229 | G protein coupled receptor (GPCR) agonists and antagonists and methods of activating and inhibiting GPCR using the same | | TUFTS MEDICAL CENTER | |  |
| 6897057 | Cell-specific and/or tumor-specific promoter retargeting of herpes gamma. 34.5 gene expression | | MASSACHUSETTS GENERAL HOSPITAL | |  |
| 7427602 | Sustained DNA delivery from structural matrices | | UNIVERSITY OF MICHIGAN | |  |
| 7696168 | G protein coupled receptor agonists and antagonists and methods of activating and inhibiting G protein coupled receptors using the same | | TUFTS MEDICAL CENTER | |  |
| 7776826 | Method for fostering bone formation and preservation | | YALE UNIVERSITY | |  |
| 7897588 | Agents and methods for enhancing bone formation | | UNIVERSITY OF CALIFORNIA LOS ANGELES | |  |
| 7994129 | Methods of using black bear parathyroid hormone | | MICHIGAN TECHNOLOGICAL UNIVERSITY | |  |
| 8008074 | Compositions and methods for improving bone mass through modulation of receptors of PTH and fragments thereof | | UNIVERSITY OF ALABAMA AT BIRMINGHAM | |  |
| 8053412 | NELL-1 peptides | | UNIVERSITY OF CALIFORNIA LOS ANGELES | |  |
| 8299046 | Synthetic triterpenoids and tricyclic-bis-enones for use in stimulating bone and cartilage growth | | DARTMOUTH COLLEGE | |  |
| 8324172 | G protein coupled receptor agonists and antagonists and methods of activating and inhibiting G protein coupled receptors using the same | | TUFTS MEDICAL CENTER | |  |
| 8389480 | G protein coupled receptor agonists and antagonists and methods of activating and inhibiting G protein coupled receptors using the same | | TUFTS MEDICAL CENTER | |  |
| 8513193 | Protecting and repairing cartilage and musculoskeletal soft tissues | | UNIVERSITY OF ROCHESTER | |  |
| 8563513 | Parathyroid hormone peptides and parathyroid hormone-related protein peptides and methods of use | | MASSACHUSETTS GENERAL HOSPITAL | |  |
| 8647840 | In vivo unnatural amino acid expression in the methylotrophic yeast Pichia pastoris | | SCRIPPS RESEARCH INSTITUTE | |  |
| 8986380 | Multilayered silk scaffolds for meniscus tissue engineering | | TUFTS UNIVERSITY BOSTON | |  |
| 9072813 | Mineralization and biological modification of biomaterial surfaces | | UNIVERSITY OF MICHIGAN | |  |
| 9109022 | Production of carrier-peptide conjugates using chemically reactive unnatural amino acids | | SCRIPPS RESEARCH INSTITUTE | |  |
| 9150849 | Directed evolution using proteins comprising unnatural amino acids | | SCRIPPS RESEARCH INSTITUTE | |  |
| 9526737 | Oxysterols for activation of hedgehog signaling, osteoinduction, antiadipogenesis, and Wnt signaling | | UNIVERSITY OF CALIFORNIA LOS ANGELES | |  |
| 9532994 | Agents and methods for enhancing bone formation by oxysterols in combination with bone morphogenic proteins | | UNIVERSITY OF CALIFORNIA LOS ANGELES | |  |
| 9670244 | Oxysterol compounds and the hedgehog pathway | | UNIVERSITY OF CALIFORNIA LOS ANGELES | |  |
| 9717742 | Oxysterol analogue OXY133 induces osteogenesis and hedgehog signaling and inhibits adipogenesis | | UNIVERSITY OF CALIFORNIA LOS ANGELES | |  |
| 9770517 | Anti-Trop-2 antibody-drug conjugates and uses thereof | | IMMUNOMEDICS, INC. | |  |
| 9670281 | Binding-triggered transcriptional switches and methods of use thereof | | UNIVERSITY OF CALIFORNIA, SAN FRANCISCO | |  |
| 9023362 | Immunological compositions as cancer therapeutics | | VANDERBILT UNIVERSITY | |  |
| 9844582 | Synergistic tumor treatment with extended-PK IL-2 and therapeutic agents | | MASSACHUSETTS INSTITUTE OF TECHNOLOGY | |  |
| 9931359 | Methods and compositions for infusion of transiently engrafting, selected populations of allogeneic lymphocytes to treat cancer | | JOHNS HOPKINS UNIVERSITY | |  |
| 9283233 | Method for on-demand contraception | | n/a | |  |
| 10369124 | Dendrimer compositions and their use in treatment of diseases of the eye | | JOHNS HOPKINS UNIVERSITY | |  |
| 10500256 | Polymers for delivery of therapeutic proteins | | UNIVERSITY OF TEXAS, AUSTIN | |  |
| 7998481 | Modulation of NKG2D for treating or preventing solid organ allograft rejection | | UNIVERSITY OF CALIFORNIA, SAN FRANCISCO | |  |
| 8298525 | Method of treating multiple sclerosis with interferon-beta and an IL-2R antagonist | | n/a | |  |
| 8394763 | Cyclic undecapeptides and derivatives as multiple sclerosis therapies | | OREGON HEALTH & SCIENCE UNIVERSITY | |  |
| 8636997 | Method of treating multiple sclerosis with interferon-beta and an IL-2R antagonist | | n/a | |  |
| 9752191 | Gene expression profiles associated with chronic allograft nephropathy | | SCRIPPS RESEARCH INSTITUTE | |  |
| 8445507 | Androgen receptor modulator for the treatment of prostate cancer and androgen receptor-associated diseases | | UNIVERSITY OF CALIFORNIA LOS ANGELES | |  |
| 8802689 | Androgen receptor modulator for the treatment of prostate cancer and androgen receptor-associated diseases | | UNIVERSITY OF CALIFORNIA LOS ANGELES | |  |
| 9388159 | Substituted diazaspiroalkanes as androgen receptor modulators | | UNIVERSITY OF CALIFORNIA LOS ANGELES | |  |
| 9987261 | Substituted diazaspiroalkanes as androgen receptor modulators | | UNIVERSITY OF CALIFORNIA LOS ANGELES | |  |
| 6872728 | Gonadotropin-releasing hormone receptor antagonists and methods relating thereto | | NEUROCRINE BIOSCIENCES, INC. | |  |
| 7056927 | Gonadotropin-releasing hormone receptor antagonists and methods relating thereto | | NEUROCRINE BIOSCIENCES, INC. | |  |
| 7176211 | Gonadotropin-releasing hormone receptor antagonists and methods relating thereto | | NEUROCRINE BIOSCIENCES, INC. | |  |
| 7179815 | Gonadotropin-releasing hormone receptor antagonists and methods relating thereto | | NEUROCRINE BIOSCIENCES, INC. | |  |
| 7419983 | Gonadotropin-releasing hormone receptor antagonists and methods related thereto | | NEUROCRINE BIOSCIENCES, INC. | |  |
| 7462625 | Gonadotropin-releasing hormone receptor antagonists and methods relating thereto | | NEUROCRINE BIOSCIENCES, INC. | |  |
| 7737168 | Compounds, compositions, and methods for treatment and prevention of orthopoxvirus infections and associated diseases | | SIGA TECHNOLOGIES, INC. | |  |
| 8039504 | Chemicals, compositions, and methods for treatment and prevention of orthopoxvirus infections and associated diseases | | SIGA TECHNOLOGIES, INC. | |  |
| 8124643 | Compounds, compositions and methods for treatment and prevention of orthopoxvirus infections and associated diseases | | SIGA TECHNOLOGIES, INC. | |  |
| 8530509 | Compounds, compositions and methods for treatment and prevention of orthopoxvirus infections and associated diseases | | SIGA TECHNOLOGIES, INC. | |  |
| 8802714 | Compounds, compositions and methods for treatment and prevention of orthopoxvirus infections and associated diseases | | SIGA TECHNOLOGIES, INC. | |  |
| 8653119 | Methods for treating transthyretin amyloid diseases | | SCRIPPS RESEARCH INSTITUTE | |  |
| 9024007 | Antisense oligonucleotides for inducing exon skipping and methods of use thereof | | UNIVERSITY OF WESTERN AUSTRALIA | |  |
| 9994851 | Antisense oligonucleotides for inducing exon skipping and methods of use thereof | | UNIVERSITY OF WESTERN AUSTRALIA | |  |
| 10071953 | Bifunctional AKR1C3 inhibitors/androgen receptor modulators and methods of use thereof | | UNIVERSITY OF PENNSYLVANIA | |  |
| 9271961 | Bifunctional AKR1C3 inhibitors/androgen receptor modulators and methods of use thereof | | UNIVERSITY OF PENNSYLVANIA | |  |
| 9932641 | Fusion genes associated with progressive prostate cancer | | UNIVERSITY OF PITTSBURGH | |  |
| 9802984 | Biomimetic peptide and biodegradable delivery platform for the treatment of angiogenesis- and lymphangiogenesis-dependent diseases | | JOHNS HOPKINS UNIVERSITY | |  |
| 9066963 | Methods of treating breast cancer with anthracycline therapy | | UNIV OF NORTH CAROLINA CHAPEL HILL | |  |
| 6916802 | Amino ceramide-like compounds and therapeutic methods of use | | UNIVERSITY OF MICHIGAN | |  |
| 7253185 | Amino ceramide-like compounds and therapeutic methods of use | | UNIVERSITY OF MICHIGAN | |  |
| 5914331 | Antiviral activity and resolution of 2-hydroxymethyl-5-(5-fluorocytosin-1-yl)-1,3-oxathiolane | | EMORY UNIVERSITY | |  |
| 7709517 | Diarylhydantoin compounds | | UNIVERSITY OF CALIFORNIA LOS ANGELES | |  |
| 8183274 | Treatment of hyperproliferative disorders with diarylhydantoin compounds | | UNIVERSITY OF CALIFORNIA LOS ANGELES | |  |
| 9126941 | Treatment of hyperproliferative disorders with diarylhydantoin compounds | | UNIVERSITY OF CALIFORNIA LOS ANGELES | |  |
| 8486907 | Antisense oligonucleotides for inducing exon skipping and methods of use thereof | | UNIVERSITY OF WESTERN AUSTRALIA | |  |
| 9018368 | Antisense oligonucleotides for inducing exon skipping and methods of use thereof | | UNIVERSITY OF WESTERN AUSTRALIA | |  |
| 10016421 | Histone deacetylase 6 inhibition for enhancing T-cell function during anti-tumor response and tumor-peptide vaccination | | H. LEE MOFFITT CANCER CTR & RES INST | |  |
| 10221140 | Asymmetric bisaminoquinolines and bisaminoquinolines with varied linkers as autophagy inhibitors for cancer and other therapy | | UNIVERSITY OF PENNSYLVANIA | |  |
| 7611702 | TNF-alpha blocker treatment for enterocolitis associated with immunostimulatory therapeutic antibody therapy | | n/a | |  |
| 8518404 | Activatable binding polypeptides and methods of identification and use thereof | | UNIVERSITY OF CALIFORNIA SANTA BARBARA | |  |
| 9169321 | Activatable binding polypeptides and methods of identification and use thereof | | UNIVERSITY OF CALIFORNIA SANTA BARBARA | |  |
| 9180214 | Gonadotropin-releasing hormone receptor-targeting peptides and their use to treat and diagnose cancer | | UNIVERSITY OF NEW MEXICO | |  |
| 9314460 | Method for cancer cell reprogramming | | UNIVERSITY OF NEW MEXICO | |  |
| 9365510 | Aziridine bisphenol ethers and related compounds and methods for their use | | BRITISH COLUMBIA CANCER AGENCY | |  |
| 9375496 | Halogenated compounds for cancer imaging and treatment and methods for their use | | BRITISH COLUMBIA CANCER AGENCY | |  |
| 9464326 | Total and phosphorylated IL-1 receptor-associated kinase-1 and IL-1 receptor-associated kinase-4 as a biomarker for cancer progression and chemotherapy resistance | | UNIVERSITY OF MARYLAND BALTIMORE | |  |
| 9579283 | Porous nanoparticle-supported lipid bilayers (protocells) for targeted delivery and methods of using same | | UNIVERSITY OF NEW MEXICO | |  |
| 9863935 | Predictive biomarkers for CTLA-4 blockade therapy and for PD-1 blockade therapy | | H. LEE MOFFITT CANCER CTR & RES INST | |  |
|  | | | | |  |
|  |  | |  | |  |
|  |  | |  | |  |
|  |  | |  | |  |
|  |  | |  | |  |
|  |  | |  | |  |
|  |  | |  | |  |
|  |  | |  | |  |
|  |  | |  | |  |

|  |  |  |
| --- | --- | --- |

**B. Patents in RePORT associated with NIH-funded project not related to a drug or drug target**

| **Patent ID** | **Patent Title** |
| --- | --- |
| 10130718 | Antibody-drug conjugates and uses thereof |
| 10137196 | Dosages of immunoconjugates of antibodies and SN-38 for improved efficacy and decreased toxicity |
| 8623377 | Joint-homing peptides and uses thereof |
| 9387250 | Therapeutic compositions for bone repair |
| 9481732 | Dosages of immunoconjugates of antibodies and SN-38 for improved efficacy and decreased toxicity |
| 7459428 | Method of regulating glucose metabolism, and reagents related thereto |
| 7807405 | Glycoprotein synthesis and remodeling by enzymatic transglycosylation |
| 9040497 | Treatment and diagnosis of colon cancer |
| 9399028 | Treatment of drug resistant cancer |
| 9492414 | Treatment and diagnosis of colon cancer |
| 10406202 | Therapeutic vitamin D conjugates |
| 6890898 | Method of regulating glucose metabolism, and reagents related thereto |
| 7078381 | Method of regulating glucose metabolism, and reagents related thereto |
| 9695224 | Stabilized insulinotropic peptides and methods of use |
| 9839646 | Small molecule enhancer for dendritic cell cancer vaccines |
