# Supplementary material for: NIH funding for patents that contribute to market exclusivity of drugs approved 2010–2019 and the public interest protections of Bayh-Dole
Source: PLoS One. 2023 Jul 26;18(7):e0288447. doi: 10.1371/journal.pone.0288447 (PMC10370755; doi:10.1371/journal.pone.0288447)
Supplement: S3 Table — This list of patents associated with the drugs studied in this report is copyrighted material from DrugPatentWatch, reproduced here with permission. (DOCX) [file pone.0288447.s003.docx]

**S3 Table. Patents in DrugPatentWatch associated with drugs approved 2010–2019.** This list of patents associated with the drugs studied in this report is copyrighted material from DrugPatentWatch, reproduced here with permission.

| **Trade Name** | **Ingredient** | **NDA** | **Patent Number** | **Expire Date** |
| --- | --- | --- | --- | --- |
| ACTEMRA | TOCILIZUMAB | 125276 | 10016338 | 3/11/2033 |
| ACTEMRA | TOCILIZUMAB | 125472 | 10016338 | 3/11/2033 |
| ACTEMRA | TOCILIZUMAB | 125276 | 10088479 | 1/13/2034 |
| ACTEMRA | TOCILIZUMAB | 125472 | 10088479 | 1/13/2034 |
| ACTEMRA | TOCILIZUMAB | 125276 | 10105418 | 11/18/2038 |
| ACTEMRA | TOCILIZUMAB | 125472 | 10105418 | 11/18/2038 |
| ACTEMRA | TOCILIZUMAB | 125276 | 10111968 | 2/15/2032 |
| ACTEMRA | TOCILIZUMAB | 125472 | 10111968 | 2/15/2032 |
| ACTEMRA | TOCILIZUMAB | 125276 | 10168326 | 7/4/2033 |
| ACTEMRA | TOCILIZUMAB | 125472 | 10168326 | 7/4/2033 |
| ACTEMRA | TOCILIZUMAB | 125276 | 10231981 | 11/8/2030 |
| ACTEMRA | TOCILIZUMAB | 125472 | 10231981 | 11/8/2030 |
| ACTEMRA | TOCILIZUMAB | 125276 | 10261083 | 1/4/2033 |
| ACTEMRA | TOCILIZUMAB | 125472 | 10261083 | 1/4/2033 |
| ACTEMRA | TOCILIZUMAB | 125276 | 10261094 | 10/31/2033 |
| ACTEMRA | TOCILIZUMAB | 125472 | 10261094 | 10/31/2033 |
| ACTEMRA | TOCILIZUMAB | 125276 | 10273252 | 4/28/2036 |
| ACTEMRA | TOCILIZUMAB | 125472 | 10273252 | 4/28/2036 |
| ACTEMRA | TOCILIZUMAB | 125276 | 10329314 | 4/4/2036 |
| ACTEMRA | TOCILIZUMAB | 125472 | 10329314 | 4/4/2036 |
| ACTEMRA | TOCILIZUMAB | 125276 | 10369114 | 3/14/2033 |
| ACTEMRA | TOCILIZUMAB | 125472 | 10369114 | 3/14/2033 |
| ACTEMRA | TOCILIZUMAB | 125276 | 10383876 | 4/5/2031 |
| ACTEMRA | TOCILIZUMAB | 125472 | 10383876 | 4/5/2031 |
| ACTEMRA | TOCILIZUMAB | 125276 | 10407673 | 8/24/2036 |
| ACTEMRA | TOCILIZUMAB | 125472 | 10407673 | 8/24/2036 |
| ACTEMRA | TOCILIZUMAB | 125276 | 10429379 | 5/12/2035 |
| ACTEMRA | TOCILIZUMAB | 125472 | 10429379 | 5/12/2035 |
| ACTEMRA | TOCILIZUMAB | 125276 | 10487098 | 4/4/2036 |
| ACTEMRA | TOCILIZUMAB | 125472 | 10487098 | 4/4/2036 |
| ACTEMRA | TOCILIZUMAB | 125276 | 10493113 | 11/14/2037 |
| ACTEMRA | TOCILIZUMAB | 125472 | 10493113 | 11/14/2037 |
| ACTEMRA | TOCILIZUMAB | 125276 | 10501769 | 10/26/2029 |
| ACTEMRA | TOCILIZUMAB | 125472 | 10501769 | 10/26/2029 |
| ACTEMRA | TOCILIZUMAB | 125276 | 10507262 | 5/11/2032 |
| ACTEMRA | TOCILIZUMAB | 125472 | 10507262 | 5/11/2032 |
| ACTEMRA | TOCILIZUMAB | 125276 | 10519228 | 5/8/2038 |
| ACTEMRA | TOCILIZUMAB | 125472 | 10519228 | 5/8/2038 |
| ACTEMRA | TOCILIZUMAB | 125276 | 10562974 | 3/13/2033 |
| ACTEMRA | TOCILIZUMAB | 125472 | 10562974 | 3/13/2033 |
| ACTEMRA | TOCILIZUMAB | 125276 | 10577154 | 5/13/2029 |
| ACTEMRA | TOCILIZUMAB | 125472 | 10577154 | 5/13/2029 |
| ACTEMRA | TOCILIZUMAB | 125276 | 10584180 | 3/26/2040 |
| ACTEMRA | TOCILIZUMAB | 125472 | 10584180 | 3/26/2040 |
| ACTEMRA | TOCILIZUMAB | 125276 | 10590182 | 2/24/2035 |
| ACTEMRA | TOCILIZUMAB | 125472 | 10590182 | 2/24/2035 |
| ACTEMRA | TOCILIZUMAB | 125276 | 10603378 | 7/13/2032 |
| ACTEMRA | TOCILIZUMAB | 125472 | 10603378 | 7/13/2032 |
| ACTEMRA | TOCILIZUMAB | 125276 | 10668053 | 5/3/2033 |
| ACTEMRA | TOCILIZUMAB | 125472 | 10668053 | 5/3/2033 |
| ACTEMRA | TOCILIZUMAB | 125276 | 10669337 | 7/25/2034 |
| ACTEMRA | TOCILIZUMAB | 125472 | 10669337 | 7/25/2034 |
| ACTEMRA | TOCILIZUMAB | 125276 | 10697883 | 5/19/2035 |
| ADCETRIS | BRENTUXIMAB VEDOTIN | 125388 | 10022451 | 10/10/2038 |
| ADCETRIS | BRENTUXIMAB VEDOTIN | 125399 | 10022451 | 10/10/2038 |
| ADCETRIS | BRENTUXIMAB VEDOTIN | 125388 | 10065934 | 7/17/2034 |
| ADCETRIS | BRENTUXIMAB VEDOTIN | 125399 | 10065934 | 7/17/2034 |
| ADCETRIS | BRENTUXIMAB VEDOTIN | 125388 | 10111968 | 2/15/2032 |
| ADCETRIS | BRENTUXIMAB VEDOTIN | 125399 | 10111968 | 2/15/2032 |
| ADCETRIS | BRENTUXIMAB VEDOTIN | 125388 | 10159675 | 12/2/2035 |
| ADCETRIS | BRENTUXIMAB VEDOTIN | 125399 | 10159675 | 12/2/2035 |
| ADCETRIS | BRENTUXIMAB VEDOTIN | 125388 | 10201615 | 10/22/2030 |
| ADCETRIS | BRENTUXIMAB VEDOTIN | 125399 | 10201615 | 10/22/2030 |
| ADCETRIS | BRENTUXIMAB VEDOTIN | 125388 | 10259859 | 8/7/2035 |
| ADCETRIS | BRENTUXIMAB VEDOTIN | 125399 | 10259859 | 8/7/2035 |
| ADCETRIS | BRENTUXIMAB VEDOTIN | 125388 | 10261083 | 1/4/2033 |
| ADCETRIS | BRENTUXIMAB VEDOTIN | 125399 | 10261083 | 1/4/2033 |
| ADCETRIS | BRENTUXIMAB VEDOTIN | 125388 | 10265291 | 7/15/2035 |
| ADCETRIS | BRENTUXIMAB VEDOTIN | 125399 | 10265291 | 7/15/2035 |
| ADCETRIS | BRENTUXIMAB VEDOTIN | 125388 | 10273252 | 4/28/2036 |
| ADCETRIS | BRENTUXIMAB VEDOTIN | 125399 | 10273252 | 4/28/2036 |
| ADCETRIS | BRENTUXIMAB VEDOTIN | 125388 | 10279014 | 5/17/2039 |
| ADCETRIS | BRENTUXIMAB VEDOTIN | 125399 | 10279014 | 5/17/2039 |
| ADCETRIS | BRENTUXIMAB VEDOTIN | 125388 | 10328157 | 7/15/2035 |
| ADCETRIS | BRENTUXIMAB VEDOTIN | 125399 | 10328157 | 7/15/2035 |
| ADCETRIS | BRENTUXIMAB VEDOTIN | 125388 | 10329314 | 4/4/2036 |
| ADCETRIS | BRENTUXIMAB VEDOTIN | 125399 | 10329314 | 4/4/2036 |
| ADCETRIS | BRENTUXIMAB VEDOTIN | 125388 | 10335494 | 12/6/2033 |
| ADCETRIS | BRENTUXIMAB VEDOTIN | 125399 | 10335494 | 12/6/2033 |
| ADCETRIS | BRENTUXIMAB VEDOTIN | 125388 | 10342870 | 8/6/2034 |
| ADCETRIS | BRENTUXIMAB VEDOTIN | 125399 | 10342870 | 8/6/2034 |
| ADCETRIS | BRENTUXIMAB VEDOTIN | 125388 | 10369077 | 8/20/2039 |
| ADCETRIS | BRENTUXIMAB VEDOTIN | 125399 | 10369077 | 8/20/2039 |
| ADCETRIS | BRENTUXIMAB VEDOTIN | 125388 | 10383792 | 8/20/2039 |
| ADCETRIS | BRENTUXIMAB VEDOTIN | 125399 | 10383792 | 8/20/2039 |
| ADCETRIS | BRENTUXIMAB VEDOTIN | 125388 | 10487098 | 4/4/2036 |
| ADCETRIS | BRENTUXIMAB VEDOTIN | 125399 | 10487098 | 4/4/2036 |
| ADCETRIS | BRENTUXIMAB VEDOTIN | 125388 | 10501436 | 1/1/2034 |
| ADCETRIS | BRENTUXIMAB VEDOTIN | 125399 | 10501436 | 1/1/2034 |
| ADCETRIS | BRENTUXIMAB VEDOTIN | 125388 | 10507165 | 5/31/2037 |
| ADCETRIS | BRENTUXIMAB VEDOTIN | 125399 | 10507165 | 5/31/2037 |
| ADCETRIS | BRENTUXIMAB VEDOTIN | 125388 | 10543283 | 3/13/2033 |
| ADCETRIS | BRENTUXIMAB VEDOTIN | 125399 | 10543283 | 3/13/2033 |
| ADCETRIS | BRENTUXIMAB VEDOTIN | 125388 | 10590182 | 2/24/2035 |
| ADCETRIS | BRENTUXIMAB VEDOTIN | 125399 | 10590182 | 2/24/2035 |
| ADCETRIS | BRENTUXIMAB VEDOTIN | 125388 | 10596141 | 5/15/2035 |
| ADCETRIS | BRENTUXIMAB VEDOTIN | 125399 | 10596141 | 5/15/2035 |
| ADCETRIS | BRENTUXIMAB VEDOTIN | 125388 | 10618935 | 11/3/2035 |
| ADCETRIS | BRENTUXIMAB VEDOTIN | 125399 | 10618935 | 11/3/2035 |
| ADCETRIS | BRENTUXIMAB VEDOTIN | 125388 | 10626100 | 12/23/2033 |
| ADCETRIS | BRENTUXIMAB VEDOTIN | 125399 | 10626100 | 12/23/2033 |
| ADCETRIS | BRENTUXIMAB VEDOTIN | 125388 | 10669337 | 7/25/2034 |
| ADCETRIS | BRENTUXIMAB VEDOTIN | 125399 | 10669337 | 7/25/2034 |
| ADLYXIN | LIXISENATIDE | 208471 | 10004747 | 5/5/2030 |
| ADLYXIN | LIXISENATIDE | 208471 | 10028910 | 11/11/2030 |
| ADLYXIN | LIXISENATIDE | 208471 | 10071992 | 2/26/2039 |
| ADLYXIN | LIXISENATIDE | 208471 | 10159713 | 3/18/2035 |
| ADLYXIN | LIXISENATIDE | 208471 | 10183116 | 1/26/2031 |
| ADLYXIN | LIXISENATIDE | 208471 | 10201663 | 3/10/2034 |
| ADLYXIN | LIXISENATIDE | 208471 | 10226583 | 3/14/2033 |
| ADLYXIN | LIXISENATIDE | 208471 | 10253102 | 10/12/2035 |
| ADLYXIN | LIXISENATIDE | 208471 | 10258639 | 5/6/2034 |
| ADLYXIN | LIXISENATIDE | 208471 | 10335489 | 1/9/2032 |
| ADLYXIN | LIXISENATIDE | 208471 | 10369114 | 3/14/2033 |
| ADLYXIN | LIXISENATIDE | 208471 | 10383918 | 8/29/2039 |
| ADLYXIN | LIXISENATIDE | 208471 | 10392357 | 6/30/2035 |
| ADLYXIN | LIXISENATIDE | 208471 | 10434147 | 3/13/2035 |
| ADLYXIN | LIXISENATIDE | 208471 | 10450343 | 3/21/2033 |
| ADLYXIN | LIXISENATIDE | 208471 | 10485851 | 6/7/2036 |
| ADLYXIN | LIXISENATIDE | 208471 | 10548952 | 6/7/2036 |
| ADLYXIN | LIXISENATIDE | 208471 | 10610572 | 7/27/2037 |
| ADLYXIN | LIXISENATIDE | 208471 | 10639308 | 3/15/2033 |
| ADLYXIN | LIXISENATIDE | 208471 | 10660939 | 4/14/2035 |
| AKYNZEO | FOSNETUPITANT CHLORIDE HYDROCHLORIDE; PALONOSETRON HYDROCHLORIDE | 210493 | 10208073 | 5/23/2032 |
| AKYNZEO | NETUPITANT; PALONOSETRON HYDROCHLORIDE | 205718 | 10233154 | 9/25/2035 |
| AKYNZEO | FOSNETUPITANT CHLORIDE HYDROCHLORIDE; PALONOSETRON HYDROCHLORIDE | 210493 | 10624911 | 6/2/2037 |
| AKYNZEO | NETUPITANT; PALONOSETRON HYDROCHLORIDE | 205718 | 5202333 | 4/13/2015 |
| ALECENSA | ALECTINIB HYDROCHLORIDE | 208434 | 10350214 | 4/24/2035 |
| ALIQOPA | COPANLISIB DIHYDROCHLORIDE | 209936 | 10383876 | 3/29/2032 |
| ALUNBRIG | BRIGATINIB | 208772 | 10385078 | 11/10/2035 |
| AMPYRA | DALFAMPRIDINE | 22250 | 5370879 | 12/6/2011 |
| ANNOVERA | ETHINYL ESTRADIOL; SEGESTERONE ACETATE | 209627 | 10632066 | 2/1/2039 |
| ANTHIM | OBILTOXAXIMAB | 125509 | 10590182 | 2/24/2035 |
| ARISTADA | ARIPIPRAZOLE LAUROXIL | 207533 | 10112903 | 6/24/2030 |
| ARISTADA | ARIPIPRAZOLE LAUROXIL | 207533 | 10226458 | 11/7/2032 |
| ARISTADA | ARIPIPRAZOLE LAUROXIL | 207533 | 10238651 | 3/19/2035 |
| AUBAGIO | TERIFLUNOMIDE | 202992 | 5459163 | 10/17/2012 |
| BAVENCIO | AVELUMAB | 761049 | 10001483 | 6/26/2035 |
| BAVENCIO | AVELUMAB | 761078 | 10001483 | 6/26/2035 |
| BAVENCIO | AVELUMAB | 761049 | 10143723 | 12/23/2035 |
| BAVENCIO | AVELUMAB | 761078 | 10143723 | 12/23/2035 |
| BAVENCIO | AVELUMAB | 761049 | 10172808 | 5/18/2036 |
| BAVENCIO | AVELUMAB | 761078 | 10172808 | 5/18/2036 |
| BAVENCIO | AVELUMAB | 761049 | 10195175 | 6/25/2035 |
| BAVENCIO | AVELUMAB | 761078 | 10195175 | 6/25/2035 |
| BAVENCIO | AVELUMAB | 761049 | 10195188 | 6/13/2036 |
| BAVENCIO | AVELUMAB | 761078 | 10195188 | 6/13/2036 |
| BAVENCIO | AVELUMAB | 761049 | 10206910 | 11/7/2034 |
| BAVENCIO | AVELUMAB | 761078 | 10206910 | 11/7/2034 |
| BAVENCIO | AVELUMAB | 761049 | 10214585 | 3/12/2034 |
| BAVENCIO | AVELUMAB | 761078 | 10214585 | 3/12/2034 |
| BAVENCIO | AVELUMAB | 761049 | 10273252 | 4/28/2036 |
| BAVENCIO | AVELUMAB | 761078 | 10273252 | 4/28/2036 |
| BAVENCIO | AVELUMAB | 761049 | 10292951 | 9/1/2035 |
| BAVENCIO | AVELUMAB | 761078 | 10292951 | 9/1/2035 |
| BAVENCIO | AVELUMAB | 761049 | 10335486 | 5/18/2036 |
| BAVENCIO | AVELUMAB | 761078 | 10335486 | 5/18/2036 |
| BAVENCIO | AVELUMAB | 761049 | 10350213 | 7/30/2039 |
| BAVENCIO | AVELUMAB | 761078 | 10350213 | 7/30/2039 |
| BAVENCIO | AVELUMAB | 761049 | 10370346 | 8/6/2033 |
| BAVENCIO | AVELUMAB | 761078 | 10370346 | 8/6/2033 |
| BAVENCIO | AVELUMAB | 761049 | 10385131 | 5/11/2036 |
| BAVENCIO | AVELUMAB | 761078 | 10385131 | 5/11/2036 |
| BAVENCIO | AVELUMAB | 761049 | 10398685 | 6/13/2036 |
| BAVENCIO | AVELUMAB | 761078 | 10398685 | 6/13/2036 |
| BAVENCIO | AVELUMAB | 761049 | 10406278 | 2/11/2036 |
| BAVENCIO | AVELUMAB | 761078 | 10406278 | 2/11/2036 |
| BAVENCIO | AVELUMAB | 761049 | 10456420 | 3/27/2035 |
| BAVENCIO | AVELUMAB | 761078 | 10456420 | 3/27/2035 |
| BAVENCIO | AVELUMAB | 761049 | 10485796 | 6/29/2035 |
| BAVENCIO | AVELUMAB | 761078 | 10485796 | 6/29/2035 |
| BAVENCIO | AVELUMAB | 761049 | 10507244 | 10/1/2035 |
| BAVENCIO | AVELUMAB | 761078 | 10507244 | 10/1/2035 |
| BAVENCIO | AVELUMAB | 761049 | 10532056 | 6/29/2035 |
| BAVENCIO | AVELUMAB | 761078 | 10532056 | 6/29/2035 |
| BAVENCIO | AVELUMAB | 761049 | 10544223 | 4/20/2037 |
| BAVENCIO | AVELUMAB | 761078 | 10544223 | 4/20/2037 |
| BAVENCIO | AVELUMAB | 761049 | 10568870 | 4/7/2036 |
| BAVENCIO | AVELUMAB | 761078 | 10568870 | 4/7/2036 |
| BAVENCIO | AVELUMAB | 761049 | 10576111 | 7/5/2037 |
| BAVENCIO | AVELUMAB | 761078 | 10576111 | 7/5/2037 |
| BAVENCIO | AVELUMAB | 761049 | 10583131 | 6/13/2036 |
| BAVENCIO | AVELUMAB | 761078 | 10583131 | 6/13/2036 |
| BAVENCIO | AVELUMAB | 761049 | 10613092 | 4/1/2036 |
| BAVENCIO | AVELUMAB | 761078 | 10613092 | 4/1/2036 |
| BAVENCIO | AVELUMAB | 761049 | 10660891 | 6/8/2038 |
| BAVENCIO | AVELUMAB | 761078 | 10660891 | 6/8/2038 |
| BAVENCIO | AVELUMAB | 761049 | 10660909 | 6/4/2040 |
| BAVENCIO | AVELUMAB | 761078 | 10660909 | 6/4/2040 |
| BAVENCIO | AVELUMAB | 761049 | 10662252 | 8/14/2032 |
| BAVENCIO | AVELUMAB | 761078 | 10662252 | 8/14/2032 |
| BAVENCIO | AVELUMAB | 761049 | 10675358 | 7/7/2036 |
| BAVENCIO | AVELUMAB | 761078 | 10675358 | 7/7/2036 |
| BELSOMRA | SUVOREXANT | 204569 | 10098892 | 5/29/2033 |
| BENLYSTA | BELIMUMAB | 125370 | 10017762 | 10/10/2038 |
| BENLYSTA | BELIMUMAB | 761043 | 10017762 | 2/26/2039 |
| BENLYSTA | BELIMUMAB | 125370 | 10111968 | 2/15/2032 |
| BENLYSTA | BELIMUMAB | 761043 | 10111968 | 2/15/2032 |
| BENLYSTA | BELIMUMAB | 125370 | 10132813 | 2/11/2031 |
| BENLYSTA | BELIMUMAB | 761043 | 10132813 | 2/11/2031 |
| BENLYSTA | BELIMUMAB | 125370 | 10259859 | 8/7/2035 |
| BENLYSTA | BELIMUMAB | 761043 | 10259859 | 8/7/2035 |
| BENLYSTA | BELIMUMAB | 125370 | 10273252 | 4/28/2036 |
| BENLYSTA | BELIMUMAB | 761043 | 10273252 | 4/28/2036 |
| BENLYSTA | BELIMUMAB | 125370 | 10316282 | 3/19/2033 |
| BENLYSTA | BELIMUMAB | 761043 | 10316282 | 3/19/2033 |
| BENLYSTA | BELIMUMAB | 125370 | 10328158 | 1/10/2034 |
| BENLYSTA | BELIMUMAB | 761043 | 10328158 | 1/10/2034 |
| BENLYSTA | BELIMUMAB | 125370 | 10329314 | 4/4/2036 |
| BENLYSTA | BELIMUMAB | 761043 | 10329314 | 4/4/2036 |
| BENLYSTA | BELIMUMAB | 125370 | 10369114 | 3/14/2033 |
| BENLYSTA | BELIMUMAB | 761043 | 10369114 | 3/14/2033 |
| BENLYSTA | BELIMUMAB | 125370 | 10407673 | 8/24/2036 |
| BENLYSTA | BELIMUMAB | 761043 | 10407673 | 8/24/2036 |
| BENLYSTA | BELIMUMAB | 125370 | 10487098 | 4/4/2036 |
| BENLYSTA | BELIMUMAB | 761043 | 10487098 | 4/4/2036 |
| BENLYSTA | BELIMUMAB | 125370 | 10507262 | 5/11/2032 |
| BENLYSTA | BELIMUMAB | 761043 | 10507262 | 5/11/2032 |
| BENLYSTA | BELIMUMAB | 125370 | 10562974 | 3/13/2033 |
| BENLYSTA | BELIMUMAB | 761043 | 10562974 | 3/13/2033 |
| BENLYSTA | BELIMUMAB | 125370 | 10584180 | 3/26/2040 |
| BENLYSTA | BELIMUMAB | 761043 | 10584180 | 3/26/2040 |
| BENLYSTA | BELIMUMAB | 125370 | 10590182 | 2/24/2035 |
| BENLYSTA | BELIMUMAB | 761043 | 10590182 | 2/24/2035 |
| BENLYSTA | BELIMUMAB | 125370 | 10668053 | 5/3/2033 |
| BENLYSTA | BELIMUMAB | 761043 | 10668053 | 5/3/2033 |
| BESPONSA | INOTUZUMAB OZOGAMICIN | 761040 | 10111968 | 2/15/2032 |
| BESPONSA | INOTUZUMAB OZOGAMICIN | 761040 | 10258619 | 10/5/2035 |
| BESPONSA | INOTUZUMAB OZOGAMICIN | 761040 | 10590182 | 2/24/2035 |
| BIKTARVY | BICTEGRAVIR SODIUM; EMTRICITABINE; TENOFOVIR ALAFENAMIDE FUMARATE | 210251 | 10385067 | 6/19/2035 |
| BIKTARVY | BICTEGRAVIR SODIUM; EMTRICITABINE; TENOFOVIR ALAFENAMIDE FUMARATE | 210251 | 10548846 | 11/8/2036 |
| BLINCYTO | BLINATUMOMAB | 125557 | 10047163 | 2/8/2033 |
| BLINCYTO | BLINATUMOMAB | 125557 | 10100034 | 5/4/2035 |
| BLINCYTO | BLINATUMOMAB | 125557 | 10111968 | 2/15/2032 |
| BLINCYTO | BLINATUMOMAB | 125557 | 10220072 | 6/30/2036 |
| BLINCYTO | BLINATUMOMAB | 125557 | 10258619 | 10/5/2035 |
| BLINCYTO | BLINATUMOMAB | 125557 | 10265291 | 7/15/2035 |
| BLINCYTO | BLINATUMOMAB | 125557 | 10328157 | 7/15/2035 |
| BLINCYTO | BLINATUMOMAB | 125557 | 10328158 | 1/10/2034 |
| BLINCYTO | BLINATUMOMAB | 125557 | 10544125 | 5/4/2035 |
| BLINCYTO | BLINATUMOMAB | 125557 | 10590182 | 2/24/2035 |
| BLINCYTO | BLINATUMOMAB | 125557 | 10596112 | 10/6/2034 |
| BLINCYTO | BLINATUMOMAB | 125557 | 10624846 | 10/6/2034 |
| BLINCYTO | BLINATUMOMAB | 125557 | 10633440 | 5/29/2034 |
| BRAFTOVI | ENCORAFENIB | 210496 | 10005761 | 8/27/2030 |
| BRAFTOVI | ENCORAFENIB | 210496 | 10258622 | 11/21/2032 |
| BRILINTA | TICAGRELOR | 22433 | 10300065 | 1/27/2036 |
| CALQUENCE | ACALABRUTINIB | 210259 | 10167291 | 7/1/2036 |
| CALQUENCE | ACALABRUTINIB | 210259 | 10239883 | 7/11/2032 |
| CALQUENCE | ACALABRUTINIB | 210259 | 10272083 | 1/21/2035 |
| CAPLYTA | LUMATEPERONE TOSYLATE | 209500 | 10464938 | 3/12/2028 |
| CINQAIR | RESLIZUMAB | 761033 | 10028940 | 8/13/2033 |
| CINQAIR | RESLIZUMAB | 761033 | 10111968 | 2/15/2032 |
| CINQAIR | RESLIZUMAB | 761033 | 10195183 | 8/13/2033 |
| CINQAIR | RESLIZUMAB | 761033 | 10195211 | 6/15/2035 |
| CINQAIR | RESLIZUMAB | 761033 | 10369114 | 3/14/2033 |
| CINQAIR | RESLIZUMAB | 761033 | 10435452 | 5/29/2035 |
| CINQAIR | RESLIZUMAB | 761033 | 10456381 | 8/13/2033 |
| CINQAIR | RESLIZUMAB | 761033 | 10577414 | 9/8/2034 |
| CINQAIR | RESLIZUMAB | 761033 | 10584180 | 3/26/2040 |
| CINQAIR | RESLIZUMAB | 761033 | 10590182 | 2/24/2035 |
| COSENTYX | SECUKINUMAB | 125504 | 10039810 | 5/15/2034 |
| COSENTYX | SECUKINUMAB | 125504 | 10111968 | 2/15/2032 |
| COSENTYX | SECUKINUMAB | 125504 | 10227403 | 6/25/2032 |
| COSENTYX | SECUKINUMAB | 125504 | 10273252 | 4/28/2036 |
| COSENTYX | SECUKINUMAB | 125504 | 10363307 | 11/5/2030 |
| COSENTYX | SECUKINUMAB | 125504 | 10369114 | 3/14/2033 |
| COSENTYX | SECUKINUMAB | 125504 | 10434172 | 8/15/2033 |
| COSENTYX | SECUKINUMAB | 125504 | 10519228 | 5/8/2038 |
| COSENTYX | SECUKINUMAB | 125504 | 10583190 | 10/8/2030 |
| COSENTYX | SECUKINUMAB | 125504 | 10590182 | 2/24/2035 |
| COTELLIC | COBIMETINIB FUMARATE | 206192 | 10478400 | 6/29/2036 |
| COTELLIC | COBIMETINIB FUMARATE | 206192 | 10590102 | 6/30/2036 |
| CYRAMZA | RAMUCIRUMAB | 125477 | 10111968 | 2/15/2032 |
| CYRAMZA | RAMUCIRUMAB | 125477 | 10189822 | 3/19/2035 |
| CYRAMZA | RAMUCIRUMAB | 125477 | 10220072 | 6/30/2036 |
| CYRAMZA | RAMUCIRUMAB | 125477 | 10227328 | 2/4/2035 |
| CYRAMZA | RAMUCIRUMAB | 125477 | 10238656 | 4/26/2039 |
| CYRAMZA | RAMUCIRUMAB | 125477 | 10259859 | 8/7/2035 |
| CYRAMZA | RAMUCIRUMAB | 125477 | 10260097 | 6/2/2031 |
| CYRAMZA | RAMUCIRUMAB | 125477 | 10265291 | 7/15/2035 |
| CYRAMZA | RAMUCIRUMAB | 125477 | 10273252 | 4/28/2036 |
| CYRAMZA | RAMUCIRUMAB | 125477 | 10314855 | 1/23/2037 |
| CYRAMZA | RAMUCIRUMAB | 125477 | 10328157 | 7/15/2035 |
| CYRAMZA | RAMUCIRUMAB | 125477 | 10383954 | 2/4/2033 |
| CYRAMZA | RAMUCIRUMAB | 125477 | 10441587 | 4/6/2035 |
| CYRAMZA | RAMUCIRUMAB | 125477 | 10588972 | 6/2/2034 |
| CYRAMZA | RAMUCIRUMAB | 125477 | 10590182 | 2/24/2035 |
| CYRAMZA | RAMUCIRUMAB | 125477 | 10596112 | 10/6/2034 |
| CYRAMZA | RAMUCIRUMAB | 125477 | 10611754 | 2/4/2035 |
| CYRAMZA | RAMUCIRUMAB | 125477 | 10639307 | 5/15/2035 |
| DARZALEX | DARATUMUMAB | 761036 | 10047163 | 2/8/2033 |
| DARZALEX | DARATUMUMAB | 761036 | 10100034 | 5/4/2035 |
| DARZALEX | DARATUMUMAB | 761036 | 10106620 | 6/16/2034 |
| DARZALEX | DARATUMUMAB | 761036 | 10213513 | 6/16/2034 |
| DARZALEX | DARATUMUMAB | 761036 | 10220072 | 6/30/2036 |
| DARZALEX | DARATUMUMAB | 761036 | 10259859 | 8/7/2035 |
| DARZALEX | DARATUMUMAB | 761036 | 10442858 | 9/21/2035 |
| DARZALEX | DARATUMUMAB | 761036 | 10456420 | 3/27/2035 |
| DARZALEX | DARATUMUMAB | 761036 | 10456479 | 10/29/2039 |
| DARZALEX | DARATUMUMAB | 761036 | 10478497 | 12/5/2039 |
| DARZALEX | DARATUMUMAB | 761036 | 10544125 | 5/4/2035 |
| DARZALEX | DARATUMUMAB | 761036 | 10590182 | 2/24/2035 |
| DAURISMO | GLASDEGIB MALEATE | 210656 | 10414748 | 4/13/2036 |
| DAYVIGO | LEMBOREXANT | 212028 | 10188652 | 10/21/2035 |
| DUPIXENT | DUPILUMAB | 761055 | 10028940 | 8/13/2033 |
| DUPIXENT | DUPILUMAB | 761055 | 10059771 | 5/7/2034 |
| DUPIXENT | DUPILUMAB | 761055 | 10066017 | 11/14/2034 |
| DUPIXENT | DUPILUMAB | 761055 | 10137193 | 9/15/2034 |
| DUPIXENT | DUPILUMAB | 761055 | 10195183 | 8/13/2033 |
| DUPIXENT | DUPILUMAB | 761055 | 10314904 | 2/19/2036 |
| DUPIXENT | DUPILUMAB | 761055 | 10369114 | 3/14/2033 |
| DUPIXENT | DUPILUMAB | 761055 | 10370449 | 9/24/2034 |
| DUPIXENT | DUPILUMAB | 761055 | 10392439 | 9/4/2039 |
| DUPIXENT | DUPILUMAB | 761055 | 10456381 | 8/13/2033 |
| DUPIXENT | DUPILUMAB | 761055 | 10485844 | 8/18/2037 |
| DUPIXENT | DUPILUMAB | 761055 | 10590182 | 2/24/2035 |
| DUPIXENT | DUPILUMAB | 761055 | 10669341 | 6/4/2033 |
| DUPIXENT | DUPILUMAB | 761055 | 10676530 | 6/4/2033 |
| ELELYSO | TALIGLUCERASE ALFA | 22458 | 10406278 | 2/11/2036 |
| ELELYSO | TALIGLUCERASE ALFA | 22458 | 10668053 | 5/3/2033 |
| ELLA | ULIPRISTAL ACETATE | 22474 | 10159681 | 4/13/2030 |
| EMPLICITI | ELOTUZUMAB | 761035 | 10017492 | 10/30/2034 |
| EMPLICITI | ELOTUZUMAB | 761035 | 10034872 | 11/23/2038 |
| EMPLICITI | ELOTUZUMAB | 761035 | 10047163 | 2/8/2033 |
| EMPLICITI | ELOTUZUMAB | 761035 | 10100034 | 5/4/2035 |
| EMPLICITI | ELOTUZUMAB | 761035 | 10111968 | 2/15/2032 |
| EMPLICITI | ELOTUZUMAB | 761035 | 10118901 | 11/12/2038 |
| EMPLICITI | ELOTUZUMAB | 761035 | 10220072 | 6/30/2036 |
| EMPLICITI | ELOTUZUMAB | 761035 | 10259859 | 8/7/2035 |
| EMPLICITI | ELOTUZUMAB | 761035 | 10369114 | 3/14/2033 |
| EMPLICITI | ELOTUZUMAB | 761035 | 10456420 | 3/27/2035 |
| EMPLICITI | ELOTUZUMAB | 761035 | 10544125 | 5/4/2035 |
| EMPLICITI | ELOTUZUMAB | 761035 | 10562974 | 3/13/2033 |
| EMPLICITI | ELOTUZUMAB | 761035 | 10590182 | 2/24/2035 |
| ENTYVIO | VEDOLIZUMAB | 125476 | 10004808 | 5/2/2031 |
| ENTYVIO | VEDOLIZUMAB | 125476 | 10040855 | 5/2/2031 |
| ENTYVIO | VEDOLIZUMAB | 125476 | 10111968 | 2/15/2032 |
| ENTYVIO | VEDOLIZUMAB | 125476 | 10143752 | 5/2/2031 |
| ENTYVIO | VEDOLIZUMAB | 125476 | 10172921 | 6/24/2034 |
| ENTYVIO | VEDOLIZUMAB | 125476 | 10273252 | 4/28/2036 |
| ENTYVIO | VEDOLIZUMAB | 125476 | 10279014 | 5/17/2039 |
| ENTYVIO | VEDOLIZUMAB | 125476 | 10324088 | 12/2/2034 |
| ENTYVIO | VEDOLIZUMAB | 125476 | 10369114 | 3/14/2033 |
| ENTYVIO | VEDOLIZUMAB | 125476 | 10422807 | 12/5/2034 |
| ENTYVIO | VEDOLIZUMAB | 125476 | 10519228 | 5/8/2038 |
| ENTYVIO | VEDOLIZUMAB | 125476 | 10532051 | 10/6/2034 |
| ENTYVIO | VEDOLIZUMAB | 125476 | 10584180 | 3/26/2040 |
| ENTYVIO | VEDOLIZUMAB | 125476 | 10590182 | 2/24/2035 |
| ENTYVIO | VEDOLIZUMAB | 125476 | 10663473 | 2/2/2036 |
| EPCLUSA | SOFOSBUVIR; VELPATASVIR | 208341 | 10086011 | 1/30/2034 |
| EPIDIOLEX | CANNABIDIOL | 210365 | 10092525 | 6/17/2035 |
| EPIDIOLEX | CANNABIDIOL | 210365 | 10111840 | 6/17/2035 |
| EPIDIOLEX | CANNABIDIOL | 210365 | 10137095 | 6/17/2035 |
| EPIDIOLEX | CANNABIDIOL | 210365 | 10195159 | 5/7/2022 |
| EPIDIOLEX | CANNABIDIOL | 210365 | 10603288 | 6/17/2035 |
| ERLEADA | APALUTAMIDE | 210951 | 10052314 | 9/23/2033 |
| ERWINAZE | asparaginase Erwinia chrysanthemi | 125359 | 10287294 | 7/8/2035 |
| ESBRIET | PIRFENIDONE | 208780 | 10188637 | 3/28/2037 |
| EXONDYS 51 | ETEPLIRSEN | 206488 | 10337003 | 3/14/2034 |
| EXONDYS 51 | ETEPLIRSEN | 206488 | 10364431 | 3/14/2034 |
| EXONDYS 51 | ETEPLIRSEN | 206488 | 10533174 | 5/4/2021 |
| EYLEA | AFLIBERCEPT | 125387 | 10004788 | 5/15/2032 |
| EYLEA | AFLIBERCEPT | 125387 | 10047096 | 10/10/2038 |
| EYLEA | AFLIBERCEPT | 125387 | 10077280 | 4/22/2036 |
| EYLEA | AFLIBERCEPT | 125387 | 10086010 | 3/18/2034 |
| EYLEA | AFLIBERCEPT | 125387 | 10093689 | 4/22/2036 |
| EYLEA | AFLIBERCEPT | 125387 | 10106605 | 12/5/2032 |
| EYLEA | AFLIBERCEPT | 125387 | 10111968 | 2/15/2032 |
| EYLEA | AFLIBERCEPT | 125387 | 10144749 | 4/22/2036 |
| EYLEA | AFLIBERCEPT | 125387 | 10220048 | 3/15/2033 |
| EYLEA | AFLIBERCEPT | 125387 | 10227328 | 2/4/2035 |
| EYLEA | AFLIBERCEPT | 125387 | 10261087 | 2/4/2034 |
| EYLEA | AFLIBERCEPT | 125387 | 10265291 | 7/15/2035 |
| EYLEA | AFLIBERCEPT | 125387 | 10280187 | 4/22/2036 |
| EYLEA | AFLIBERCEPT | 125387 | 10287294 | 7/8/2035 |
| EYLEA | AFLIBERCEPT | 125387 | 10301335 | 4/22/2036 |
| EYLEA | AFLIBERCEPT | 125387 | 10328157 | 7/15/2035 |
| EYLEA | AFLIBERCEPT | 125387 | 10342790 | 2/20/2037 |
| EYLEA | AFLIBERCEPT | 125387 | 10350306 | 2/15/2033 |
| EYLEA | AFLIBERCEPT | 125387 | 10369124 | 4/30/2034 |
| EYLEA | AFLIBERCEPT | 125387 | 10383954 | 2/4/2033 |
| EYLEA | AFLIBERCEPT | 125387 | 10426779 | 10/23/2039 |
| EYLEA | AFLIBERCEPT | 125387 | 10426817 | 10/23/2039 |
| EYLEA | AFLIBERCEPT | 125387 | 10501523 | 7/18/2034 |
| EYLEA | AFLIBERCEPT | 125387 | 10519226 | 10/11/2032 |
| EYLEA | AFLIBERCEPT | 125387 | 10525104 | 1/30/2040 |
| EYLEA | AFLIBERCEPT | 125387 | 10537563 | 10/14/2036 |
| EYLEA | AFLIBERCEPT | 125387 | 10550170 | 11/23/2035 |
| EYLEA | AFLIBERCEPT | 125387 | 10568934 | 5/7/2032 |
| EYLEA | AFLIBERCEPT | 125387 | 10576128 | 1/26/2036 |
| EYLEA | AFLIBERCEPT | 125387 | 10588855 | 5/12/2028 |
| EYLEA | AFLIBERCEPT | 125387 | 10588972 | 6/2/2034 |
| EYLEA | AFLIBERCEPT | 125387 | 10596141 | 5/15/2035 |
| EYLEA | AFLIBERCEPT | 125387 | 10611754 | 2/4/2035 |
| EYLEA | AFLIBERCEPT | 125387 | 10639307 | 5/15/2035 |
| EYLEA | AFLIBERCEPT | 125387 | 10646546 | 4/26/2037 |
| GALAFOLD | MIGALASTAT HYDROCHLORIDE | 208623 | 10076514 | 3/15/2037 |
| GALAFOLD | MIGALASTAT HYDROCHLORIDE | 208623 | 10251873 | 5/30/2038 |
| GALAFOLD | MIGALASTAT HYDROCHLORIDE | 208623 | 10383864 | 5/16/2027 |
| GALAFOLD | MIGALASTAT HYDROCHLORIDE | 208623 | 10406143 | 5/16/2027 |
| GALAFOLD | MIGALASTAT HYDROCHLORIDE | 208623 | 10471053 | 5/30/2038 |
| GALAFOLD | MIGALASTAT HYDROCHLORIDE | 208623 | 10525045 | 4/28/2028 |
| GAZYVA | OBINUTUZUMAB | 125486 | 10035848 | 1/8/2034 |
| GAZYVA | OBINUTUZUMAB | 125486 | 10092569 | 2/21/2034 |
| GAZYVA | OBINUTUZUMAB | 125486 | 10159675 | 12/2/2035 |
| GAZYVA | OBINUTUZUMAB | 125486 | 10220072 | 6/30/2036 |
| GAZYVA | OBINUTUZUMAB | 125486 | 10227328 | 2/4/2035 |
| GAZYVA | OBINUTUZUMAB | 125486 | 10251906 | 3/12/2033 |
| GAZYVA | OBINUTUZUMAB | 125486 | 10258619 | 10/5/2035 |
| GAZYVA | OBINUTUZUMAB | 125486 | 10259859 | 8/7/2035 |
| GAZYVA | OBINUTUZUMAB | 125486 | 10261083 | 1/4/2033 |
| GAZYVA | OBINUTUZUMAB | 125486 | 10265291 | 7/15/2035 |
| GAZYVA | OBINUTUZUMAB | 125486 | 10272083 | 5/17/2039 |
| GAZYVA | OBINUTUZUMAB | 125486 | 10328157 | 7/15/2035 |
| GAZYVA | OBINUTUZUMAB | 125486 | 10329314 | 4/4/2036 |
| GAZYVA | OBINUTUZUMAB | 125486 | 10485797 | 12/18/2034 |
| GAZYVA | OBINUTUZUMAB | 125486 | 10487098 | 4/4/2036 |
| GAZYVA | OBINUTUZUMAB | 125486 | 10590182 | 2/24/2035 |
| GAZYVA | OBINUTUZUMAB | 125486 | 10596112 | 10/6/2034 |
| GAZYVA | OBINUTUZUMAB | 125486 | 10610484 | 10/6/2034 |
| GAZYVA | OBINUTUZUMAB | 125486 | 10611754 | 2/4/2035 |
| GAZYVA | OBINUTUZUMAB | 125486 | 10618969 | 4/27/2040 |
| GAZYVA | OBINUTUZUMAB | 125486 | 10624846 | 10/6/2034 |
| GAZYVA | OBINUTUZUMAB | 125486 | 10669337 | 7/25/2034 |
| GENVOYA | COBICISTAT; ELVITEGRAVIR; EMTRICITABINE; TENOFOVIR ALAFENAMIDE FUMARATE | 207561 | 10039718 | 10/4/2032 |
| GIAPREZA | ANGIOTENSIN II ACETATE | 209360 | 10028995 | 12/18/2034 |
| GIAPREZA | ANGIOTENSIN II ACETATE | 209360 | 10335451 | 12/16/2029 |
| GIAPREZA | ANGIOTENSIN II ACETATE | 209360 | 10493124 | 12/18/2034 |
| GIAPREZA | ANGIOTENSIN II ACETATE | 209360 | 10500247 | 12/16/2029 |
| GIAPREZA | ANGIOTENSIN II ACETATE | 209360 | 10548943 | 12/16/2029 |
| GILENYA | FINGOLIMOD HYDROCHLORIDE | 22527 | 10543179 | 12/25/2027 |
| GILOTRIF | AFATINIB DIMALEATE | 201292 | 10004743 | 7/5/2030 |
| GIVLAARI | GIVOSIRAN SODIUM | 212194 | 10119143 | 10/3/2034 |
| GIVLAARI | GIVOSIRAN SODIUM | 212194 | 10125364 | 3/15/2033 |
| GIVLAARI | GIVOSIRAN SODIUM | 212194 | 10131907 | 8/24/2028 |
| GIVLAARI | GIVOSIRAN SODIUM | 212194 | 10273477 | 3/8/2024 |
| HARVONI | LEDIPASVIR; SOFOSBUVIR | 205834 | 10039779 | 1/30/2034 |
| HARVONI | LEDIPASVIR; SOFOSBUVIR | 205834 | 10039779*PED | 7/30/2034 |
| HARVONI | LEDIPASVIR; SOFOSBUVIR | 205834 | 10456414 | 9/14/2032 |
| HARVONI | LEDIPASVIR; SOFOSBUVIR | 212477 | 10456414 | 9/14/2032 |
| HETLIOZ | TASIMELTEON | 205677 | 10071977 | 2/12/2035 |
| HETLIOZ | TASIMELTEON | 205677 | 10149829 | 1/25/2033 |
| HETLIOZ | TASIMELTEON | 205677 | 10376487 | 7/27/2035 |
| HETLIOZ | TASIMELTEON | 205677 | 10449176 | 1/25/2033 |
| HETLIOZ | TASIMELTEON | 205677 | 10610510 | 1/25/2033 |
| HETLIOZ | TASIMELTEON | 205677 | 10610511 | 10/10/2034 |
| IDHIFA | ENASIDENIB MESYLATE | 209606 | 10093654 | 8/1/2034 |
| IDHIFA | ENASIDENIB MESYLATE | 209606 | 10294215 | 1/7/2033 |
| IDHIFA | ENASIDENIB MESYLATE | 209606 | 10610125 | 6/21/2030 |
| IMBRUVICA | IBRUTINIB | 205552 | 10004746 | 6/3/2031 |
| IMBRUVICA | IBRUTINIB | 210563 | 10004746 | 6/3/2031 |
| IMBRUVICA | IBRUTINIB | 210563 | 10010507 | 3/3/2036 |
| IMBRUVICA | IBRUTINIB | 205552 | 10016435 | 6/3/2031 |
| IMBRUVICA | IBRUTINIB | 210563 | 10016435 | 6/3/2031 |
| IMBRUVICA | IBRUTINIB | 205552 | 10106548 | 6/3/2033 |
| IMBRUVICA | IBRUTINIB | 210563 | 10106548 | 6/3/2033 |
| IMBRUVICA | IBRUTINIB | 205552 | 10125140 | 6/3/2033 |
| IMBRUVICA | IBRUTINIB | 210563 | 10125140 | 6/3/2033 |
| IMBRUVICA | IBRUTINIB | 210563 | 10213386 | 3/3/2036 |
| IMBRUVICA | IBRUTINIB | 205552 | 10294231 | 6/3/2033 |
| IMBRUVICA | IBRUTINIB | 205552 | 10294232 | 6/3/2033 |
| IMBRUVICA | IBRUTINIB | 205552 | 10463668 | 10/24/2034 |
| IMBRUVICA | IBRUTINIB | 210563 | 10463668 | 10/24/2034 |
| IMBRUVICA | IBRUTINIB | 205552 | 10478439 | 6/3/2031 |
| IMBRUVICA | IBRUTINIB | 210563 | 10478439 | 6/3/2031 |
| IMFINZI | DURVALUMAB | 761069 | 10001483 | 6/26/2035 |
| IMFINZI | DURVALUMAB | 761069 | 10106546 | 11/5/2034 |
| IMFINZI | DURVALUMAB | 761069 | 10143723 | 12/23/2035 |
| IMFINZI | DURVALUMAB | 761069 | 10172808 | 5/18/2036 |
| IMFINZI | DURVALUMAB | 761069 | 10189833 | 5/6/2035 |
| IMFINZI | DURVALUMAB | 761069 | 10195175 | 6/25/2035 |
| IMFINZI | DURVALUMAB | 761069 | 10195188 | 6/13/2036 |
| IMFINZI | DURVALUMAB | 761069 | 10195273 | 6/5/2036 |
| IMFINZI | DURVALUMAB | 761069 | 10214585 | 3/12/2034 |
| IMFINZI | DURVALUMAB | 761069 | 10258619 | 10/5/2035 |
| IMFINZI | DURVALUMAB | 761069 | 10266605 | 4/27/2036 |
| IMFINZI | DURVALUMAB | 761069 | 10273252 | 4/28/2036 |
| IMFINZI | DURVALUMAB | 761069 | 10300138 | 6/5/2036 |
| IMFINZI | DURVALUMAB | 761069 | 10300139 | 6/5/2036 |
| IMFINZI | DURVALUMAB | 761069 | 10335486 | 5/18/2036 |
| IMFINZI | DURVALUMAB | 761069 | 10350213 | 7/30/2039 |
| IMFINZI | DURVALUMAB | 761069 | 10363308 | 6/5/2036 |
| IMFINZI | DURVALUMAB | 761069 | 10370346 | 8/6/2033 |
| IMFINZI | DURVALUMAB | 761069 | 10385131 | 5/11/2036 |
| IMFINZI | DURVALUMAB | 761069 | 10398685 | 6/13/2036 |
| IMFINZI | DURVALUMAB | 761069 | 10406278 | 2/11/2036 |
| IMFINZI | DURVALUMAB | 761069 | 10421811 | 4/25/2036 |
| IMFINZI | DURVALUMAB | 761069 | 10507244 | 10/1/2035 |
| IMFINZI | DURVALUMAB | 761069 | 10533014 | 11/5/2034 |
| IMFINZI | DURVALUMAB | 761069 | 10544223 | 4/20/2037 |
| IMFINZI | DURVALUMAB | 761069 | 10568870 | 4/7/2036 |
| IMFINZI | DURVALUMAB | 761069 | 10576111 | 7/5/2037 |
| IMFINZI | DURVALUMAB | 761069 | 10583131 | 6/13/2036 |
| IMFINZI | DURVALUMAB | 761069 | 10590182 | 2/24/2035 |
| IMFINZI | DURVALUMAB | 761069 | 10603379 | 6/5/2036 |
| IMFINZI | DURVALUMAB | 761069 | 10653793 | 12/13/2032 |
| IMFINZI | DURVALUMAB | 761069 | 10660891 | 6/8/2038 |
| IMFINZI | DURVALUMAB | 761069 | 10660909 | 6/4/2040 |
| IMFINZI | DURVALUMAB | 761069 | 10662252 | 8/14/2032 |
| IMFINZI | DURVALUMAB | 761069 | 10669338 | 6/17/2036 |
| IMLYGIC | TALIMOGENE LAHERPAREPVEC | 125518 | 10034938 | 8/30/2032 |
| IMLYGIC | TALIMOGENE LAHERPAREPVEC | 125518 | 10100034 | 5/4/2035 |
| IMLYGIC | TALIMOGENE LAHERPAREPVEC | 125518 | 10544125 | 5/4/2035 |
| IMLYGIC | TALIMOGENE LAHERPAREPVEC | 125518 | 10626463 | 8/3/2032 |
| INGREZZA | VALBENAZINE TOSYLATE | 209241 | 10065952 | 10/28/2036 |
| INREBIC | FEDRATINIB HYDROCHLORIDE | 212327 | 10391094 | 5/29/2032 |
| INVOKANA | CANAGLIFLOZIN | 204042 | 10617668 | 5/11/2031 |
| JAKAFI | RUXOLITINIB PHOSPHATE | 202192 | 10016429 | 6/12/2028 |
| JARDIANCE | EMPAGLIFLOZIN | 204629 | 10258637 | 4/3/2034 |
| JARDIANCE | EMPAGLIFLOZIN | 204629 | 10406172 | 6/15/2030 |
| JUXTAPID | LOMITAPIDE MESYLATE | 203858 | 10016404 | 3/7/2025 |
| JUXTAPID | LOMITAPIDE MESYLATE | 203858 | 10555938 | 3/7/2025 |
| KADCYLA | ADO-TRASTUZUMAB EMTANSINE | 125427 | 10005783 | 10/21/2029 |
| KADCYLA | ADO-TRASTUZUMAB EMTANSINE | 125427 | 10022451 | 10/10/2038 |
| KADCYLA | ADO-TRASTUZUMAB EMTANSINE | 125427 | 10265291 | 7/15/2035 |
| KADCYLA | ADO-TRASTUZUMAB EMTANSINE | 125427 | 10287294 | 7/8/2035 |
| KADCYLA | ADO-TRASTUZUMAB EMTANSINE | 125427 | 10328157 | 7/15/2035 |
| KADCYLA | ADO-TRASTUZUMAB EMTANSINE | 125427 | 10391055 | 10/6/2034 |
| KADCYLA | ADO-TRASTUZUMAB EMTANSINE | 125427 | 10562974 | 3/13/2033 |
| KADCYLA | ADO-TRASTUZUMAB EMTANSINE | 125427 | 10624874 | 4/27/2040 |
| KALYDECO | IVACAFTOR | 207925 | 10272046 | 2/27/2033 |
| KALYDECO | IVACAFTOR | 203188 | 10646481 | 8/13/2029 |
| KANUMA | SEBELIPASE ALFA | 125561 | 4177107 | 12/4/1996 |
| KANUMA | SEBELIPASE ALFA | 125561 | 4268994 | 5/26/1998 |
| KANUMA | SEBELIPASE ALFA | 125561 | 4917820 | 4/17/2007 |
| KENGREAL | CANGRELOR | 204958 | 10039780 | 7/10/2035 |
| KEVZARA | SARILUMAB | 761037 | 10111968 | 2/15/2032 |
| KEVZARA | SARILUMAB | 761037 | 10261094 | 10/31/2033 |
| KEVZARA | SARILUMAB | 761037 | 10369114 | 3/14/2033 |
| KEVZARA | SARILUMAB | 761037 | 10519228 | 5/8/2038 |
| KEVZARA | SARILUMAB | 761037 | 10590182 | 2/24/2035 |
| KEYTRUDA | PEMBROLIZUMAB | 125514 | 10001483 | 6/26/2035 |
| KEYTRUDA | PEMBROLIZUMAB | 125514 | 10004812 | 10/10/2038 |
| KEYTRUDA | PEMBROLIZUMAB | 125514 | 10017492 | 10/30/2034 |
| KEYTRUDA | PEMBROLIZUMAB | 125514 | 10092645 | 6/17/2034 |
| KEYTRUDA | PEMBROLIZUMAB | 125514 | 10100034 | 5/4/2035 |
| KEYTRUDA | PEMBROLIZUMAB | 125514 | 10106546 | 11/5/2034 |
| KEYTRUDA | PEMBROLIZUMAB | 125514 | 10111900 | 11/6/2034 |
| KEYTRUDA | PEMBROLIZUMAB | 125514 | 10111954 | 12/4/2032 |
| KEYTRUDA | PEMBROLIZUMAB | 125514 | 10130718 | 1/7/2033 |
| KEYTRUDA | PEMBROLIZUMAB | 125514 | 10137202 | 2/6/2034 |
| KEYTRUDA | PEMBROLIZUMAB | 125514 | 10143723 | 12/23/2035 |
| KEYTRUDA | PEMBROLIZUMAB | 125514 | 10144779 | 5/29/2035 |
| KEYTRUDA | PEMBROLIZUMAB | 125514 | 10160747 | 3/16/2036 |
| KEYTRUDA | PEMBROLIZUMAB | 125514 | 10166290 | 1/22/2039 |
| KEYTRUDA | PEMBROLIZUMAB | 125514 | 10172808 | 5/18/2036 |
| KEYTRUDA | PEMBROLIZUMAB | 125514 | 10188730 | 8/19/2034 |
| KEYTRUDA | PEMBROLIZUMAB | 125514 | 10189797 | 12/30/2035 |
| KEYTRUDA | PEMBROLIZUMAB | 125514 | 10189822 | 3/19/2035 |
| KEYTRUDA | PEMBROLIZUMAB | 125514 | 10195175 | 6/25/2035 |
| KEYTRUDA | PEMBROLIZUMAB | 125514 | 10195188 | 6/13/2036 |
| KEYTRUDA | PEMBROLIZUMAB | 125514 | 10195273 | 6/5/2036 |
| KEYTRUDA | PEMBROLIZUMAB | 125514 | 10206910 | 11/7/2034 |
| KEYTRUDA | PEMBROLIZUMAB | 125514 | 10220072 | 6/30/2036 |
| KEYTRUDA | PEMBROLIZUMAB | 125514 | 10227328 | 2/4/2035 |
| KEYTRUDA | PEMBROLIZUMAB | 125514 | 10258619 | 10/5/2035 |
| KEYTRUDA | PEMBROLIZUMAB | 125514 | 10259859 | 8/7/2035 |
| KEYTRUDA | PEMBROLIZUMAB | 125514 | 10265291 | 7/15/2035 |
| KEYTRUDA | PEMBROLIZUMAB | 125514 | 10266605 | 4/27/2036 |
| KEYTRUDA | PEMBROLIZUMAB | 125514 | 10272113 | 3/29/2037 |
| KEYTRUDA | PEMBROLIZUMAB | 125514 | 10273252 | 4/28/2036 |
| KEYTRUDA | PEMBROLIZUMAB | 125514 | 10278984 | 5/25/2039 |
| KEYTRUDA | PEMBROLIZUMAB | 125514 | 10292951 | 9/1/2035 |
| KEYTRUDA | PEMBROLIZUMAB | 125514 | 10294299 | 1/22/2036 |
| KEYTRUDA | PEMBROLIZUMAB | 125514 | 10300138 | 6/5/2036 |
| KEYTRUDA | PEMBROLIZUMAB | 125514 | 10300139 | 6/5/2036 |
| KEYTRUDA | PEMBROLIZUMAB | 125514 | 10314910 | 1/10/2036 |
| KEYTRUDA | PEMBROLIZUMAB | 125514 | 10323004 | 5/4/2036 |
| KEYTRUDA | PEMBROLIZUMAB | 125514 | 10328157 | 7/15/2035 |
| KEYTRUDA | PEMBROLIZUMAB | 125514 | 10335388 | 4/17/2035 |
| KEYTRUDA | PEMBROLIZUMAB | 125514 | 10335486 | 5/18/2036 |
| KEYTRUDA | PEMBROLIZUMAB | 125514 | 10342790 | 2/20/2037 |
| KEYTRUDA | PEMBROLIZUMAB | 125514 | 10350213 | 7/30/2039 |
| KEYTRUDA | PEMBROLIZUMAB | 125514 | 10351625 | 2/19/2035 |
| KEYTRUDA | PEMBROLIZUMAB | 125514 | 10351627 | 7/27/2039 |
| KEYTRUDA | PEMBROLIZUMAB | 125514 | 10358496 | 3/1/2036 |
| KEYTRUDA | PEMBROLIZUMAB | 125514 | 10363308 | 6/5/2036 |
| KEYTRUDA | PEMBROLIZUMAB | 125514 | 10369114 | 3/14/2033 |
| KEYTRUDA | PEMBROLIZUMAB | 125514 | 10370346 | 8/6/2033 |
| KEYTRUDA | PEMBROLIZUMAB | 125514 | 10370374 | 9/21/2035 |
| KEYTRUDA | PEMBROLIZUMAB | 125514 | 10385130 | 5/11/2036 |
| KEYTRUDA | PEMBROLIZUMAB | 125514 | 10392442 | 12/17/2035 |
| KEYTRUDA | PEMBROLIZUMAB | 125514 | 10398685 | 6/13/2036 |
| KEYTRUDA | PEMBROLIZUMAB | 125514 | 10406278 | 2/11/2036 |
| KEYTRUDA | PEMBROLIZUMAB | 125514 | 10420838 | 4/8/2034 |
| KEYTRUDA | PEMBROLIZUMAB | 125514 | 10426847 | 3/26/2035 |
| KEYTRUDA | PEMBROLIZUMAB | 125514 | 10426972 | 3/30/2037 |
| KEYTRUDA | PEMBROLIZUMAB | 125514 | 10428067 | 6/7/2037 |
| KEYTRUDA | PEMBROLIZUMAB | 125514 | 10434082 | 7/26/2032 |
| KEYTRUDA | PEMBROLIZUMAB | 125514 | 10463686 | 9/15/2036 |
| KEYTRUDA | PEMBROLIZUMAB | 125514 | 10485796 | 6/29/2035 |
| KEYTRUDA | PEMBROLIZUMAB | 125514 | 10492723 | 2/27/2037 |
| KEYTRUDA | PEMBROLIZUMAB | 125514 | 10507244 | 10/1/2035 |
| KEYTRUDA | PEMBROLIZUMAB | 125514 | 10508085 | 9/22/2036 |
| KEYTRUDA | PEMBROLIZUMAB | 125514 | 10512662 | 2/25/2036 |
| KEYTRUDA | PEMBROLIZUMAB | 125514 | 10532056 | 6/29/2035 |
| KEYTRUDA | PEMBROLIZUMAB | 125514 | 10533014 | 11/5/2034 |
| KEYTRUDA | PEMBROLIZUMAB | 125514 | 10544099 | 5/4/2036 |
| KEYTRUDA | PEMBROLIZUMAB | 125514 | 10544125 | 5/4/2035 |
| KEYTRUDA | PEMBROLIZUMAB | 125514 | 10544223 | 4/20/2037 |
| KEYTRUDA | PEMBROLIZUMAB | 125514 | 10555976 | 2/4/2031 |
| KEYTRUDA | PEMBROLIZUMAB | 125514 | 10555981 | 7/16/2034 |
| KEYTRUDA | PEMBROLIZUMAB | 125514 | 10556957 | 2/29/2040 |
| KEYTRUDA | PEMBROLIZUMAB | 125514 | 10561632 | 7/26/2032 |
| KEYTRUDA | PEMBROLIZUMAB | 125514 | 10561653 | 8/11/2035 |
| KEYTRUDA | PEMBROLIZUMAB | 125514 | 10570202 | 2/4/2034 |
| KEYTRUDA | PEMBROLIZUMAB | 125514 | 10576111 | 7/5/2037 |
| KEYTRUDA | PEMBROLIZUMAB | 125514 | 10576136 | 12/23/2036 |
| KEYTRUDA | PEMBROLIZUMAB | 125514 | 10583131 | 6/13/2036 |
| KEYTRUDA | PEMBROLIZUMAB | 125514 | 10584169 | 1/24/2037 |
| KEYTRUDA | PEMBROLIZUMAB | 125514 | 10590182 | 2/24/2035 |
| KEYTRUDA | PEMBROLIZUMAB | 125514 | 10596112 | 10/6/2034 |
| KEYTRUDA | PEMBROLIZUMAB | 125514 | 10597411 | 4/1/2040 |
| KEYTRUDA | PEMBROLIZUMAB | 125514 | 10603379 | 6/5/2036 |
| KEYTRUDA | PEMBROLIZUMAB | 125514 | 10604542 | 1/11/2036 |
| KEYTRUDA | PEMBROLIZUMAB | 125514 | 10611754 | 2/4/2035 |
| KEYTRUDA | PEMBROLIZUMAB | 125514 | 10613092 | 4/1/2036 |
| KEYTRUDA | PEMBROLIZUMAB | 125514 | 10617758 | 10/9/2035 |
| KEYTRUDA | PEMBROLIZUMAB | 125514 | 10618955 | 7/15/2034 |
| KEYTRUDA | PEMBROLIZUMAB | 125514 | 10618958 | 8/19/2034 |
| KEYTRUDA | PEMBROLIZUMAB | 125514 | 10632193 | 12/24/2034 |
| KEYTRUDA | PEMBROLIZUMAB | 125514 | 10633342 | 5/4/2036 |
| KEYTRUDA | PEMBROLIZUMAB | 125514 | 10633374 | 8/6/2035 |
| KEYTRUDA | PEMBROLIZUMAB | 125514 | 10646517 | 3/29/2037 |
| KEYTRUDA | PEMBROLIZUMAB | 125514 | 10647771 | 9/21/2035 |
| KEYTRUDA | PEMBROLIZUMAB | 125514 | 10653793 | 12/13/2032 |
| KEYTRUDA | PEMBROLIZUMAB | 125514 | 10656144 | 12/2/2036 |
| KEYTRUDA | PEMBROLIZUMAB | 125514 | 10660891 | 6/8/2038 |
| KEYTRUDA | PEMBROLIZUMAB | 125514 | 10660909 | 6/4/2040 |
| KEYTRUDA | PEMBROLIZUMAB | 125514 | 10662252 | 8/14/2032 |
| KEYTRUDA | PEMBROLIZUMAB | 125514 | 10668152 | 12/17/2035 |
| KEYTRUDA | PEMBROLIZUMAB | 125514 | 10669337 | 7/25/2034 |
| KEYTRUDA | PEMBROLIZUMAB | 125514 | 10669338 | 6/17/2036 |
| KRYSTEXXA | PEGLOTICASE | 125293 | 10668053 | 5/3/2033 |
| KYBELLA | DEOXYCHOLIC ACID | 206333 | 10500214 | 3/2/2030 |
| LARTRUVO | OLARATUMAB | 761038 | 10047163 | 2/8/2033 |
| LARTRUVO | OLARATUMAB | 761038 | 10111968 | 2/15/2032 |
| LARTRUVO | OLARATUMAB | 761038 | 10590182 | 2/24/2035 |
| LASTACAFT | ALCAFTADINE | 22134 | 10617695 | 3/19/2027 |
| LENVIMA | LENVATINIB MESYLATE | 206947 | 10259791 | 8/26/2035 |
| LENVIMA | LENVATINIB MESYLATE | 206947 | 10407393 | 8/26/2035 |
| LONSURF | TIPIRACIL HYDROCHLORIDE; TRIFLURIDINE | 207981 | 10456399 | 2/3/2037 |
| LONSURF | TIPIRACIL HYDROCHLORIDE; TRIFLURIDINE | 207981 | 10457666 | 6/17/2034 |
| LORBRENA | LORLATINIB | 210868 | 10420749 | 7/27/2036 |
| LUMIZYME | ALGLUCOSIDASE ALFA | 125291 | 10016338 | 3/11/2033 |
| LUMIZYME | ALGLUCOSIDASE ALFA | 125291 | 10577154 | 5/13/2029 |
| LUMIZYME | ALGLUCOSIDASE ALFA | 125291 | 10668053 | 5/3/2033 |
| MAVYRET | GLECAPREVIR; PIBRENTASVIR | 209394 | 10028937 | 6/10/2030 |
| MAVYRET | GLECAPREVIR; PIBRENTASVIR | 209394 | 10039754 | 6/10/2030 |
| MAVYRET | GLECAPREVIR; PIBRENTASVIR | 209394 | 10286029 | 3/14/2034 |
| MEKTOVI | BINIMETINIB | 210498 | 10005761 | 8/27/2030 |
| MYALEPT | METRELEPTIN | 125390 | 10519211 | 12/2/2036 |
| NATPARA | PARATHYROID HORMONE | 125511 | 10011643 | 6/7/2031 |
| NATPARA | PARATHYROID HORMONE | 125511 | 10016338 | 3/11/2033 |
| NATPARA | PARATHYROID HORMONE | 125511 | 10028955 | 3/29/2033 |
| NATPARA | PARATHYROID HORMONE | 125511 | 10029015 | 7/5/2024 |
| NATPARA | PARATHYROID HORMONE | 125511 | 10040848 | 2/15/2033 |
| NATPARA | PARATHYROID HORMONE | 125511 | 10046000 | 6/29/2032 |
| NATPARA | PARATHYROID HORMONE | 125511 | 10046058 | 12/2/2034 |
| NATPARA | PARATHYROID HORMONE | 125511 | 10047343 | 7/20/2032 |
| NATPARA | PARATHYROID HORMONE | 125511 | 10071166 | 11/13/2023 |
| NATPARA | PARATHYROID HORMONE | 125511 | 10071171 | 7/23/2028 |
| NATPARA | PARATHYROID HORMONE | 125511 | 10081803 | 5/16/2031 |
| NATPARA | PARATHYROID HORMONE | 125511 | 10087253 | 4/14/2023 |
| NATPARA | PARATHYROID HORMONE | 125511 | 10099019 | 10/17/2038 |
| NATPARA | PARATHYROID HORMONE | 125511 | 10118965 | 9/25/2035 |
| NATPARA | PARATHYROID HORMONE | 125511 | 10130581 | 2/22/2026 |
| NATPARA | PARATHYROID HORMONE | 125511 | 10130686 | 9/15/2025 |
| NATPARA | PARATHYROID HORMONE | 125511 | 10130709 | 6/17/2031 |
| NATPARA | PARATHYROID HORMONE | 125511 | 10131712 | 8/14/2032 |
| NATPARA | PARATHYROID HORMONE | 125511 | 10137196 | 12/13/2032 |
| NATPARA | PARATHYROID HORMONE | 125511 | 10154856 | 2/6/2033 |
| NATPARA | PARATHYROID HORMONE | 125511 | 10154957 | 11/30/2032 |
| NATPARA | PARATHYROID HORMONE | 125511 | 10155069 | 12/20/2038 |
| NATPARA | PARATHYROID HORMONE | 125511 | 10172850 | 12/29/2029 |
| NATPARA | PARATHYROID HORMONE | 125511 | 10174101 | 8/10/2031 |
| NATPARA | PARATHYROID HORMONE | 125511 | 10179228 | 12/24/2029 |
| NATPARA | PARATHYROID HORMONE | 125511 | 10182561 | 12/29/2024 |
| NATPARA | PARATHYROID HORMONE | 125511 | 10183997 | 9/25/2035 |
| NATPARA | PARATHYROID HORMONE | 125511 | 10188621 | 5/6/2024 |
| NATPARA | PARATHYROID HORMONE | 125511 | 10201672 | 3/4/2029 |
| NATPARA | PARATHYROID HORMONE | 125511 | 10202419 | 5/2/2023 |
| NATPARA | PARATHYROID HORMONE | 125511 | 10220047 | 8/7/2034 |
| NATPARA | PARATHYROID HORMONE | 125511 | 10226358 | 1/12/2030 |
| NATPARA | PARATHYROID HORMONE | 125511 | 10233228 | 4/9/2030 |
| NATPARA | PARATHYROID HORMONE | 125511 | 10238848 | 4/16/2027 |
| NATPARA | PARATHYROID HORMONE | 125511 | 10239922 | 3/23/2032 |
| NATPARA | PARATHYROID HORMONE | 125511 | 10241119 | 5/7/2035 |
| NATPARA | PARATHYROID HORMONE | 125511 | 10247737 | 9/9/2033 |
| NATPARA | PARATHYROID HORMONE | 125511 | 10252039 | 12/24/2029 |
| NATPARA | PARATHYROID HORMONE | 125511 | 10266605 | 4/27/2036 |
| NATPARA | PARATHYROID HORMONE | 125511 | 10280230 | 9/10/2026 |
| NATPARA | PARATHYROID HORMONE | 125511 | 10300212 | 5/28/2039 |
| NATPARA | PARATHYROID HORMONE | 125511 | 10302660 | 4/2/2028 |
| NATPARA | PARATHYROID HORMONE | 125511 | 10308715 | 10/11/2027 |
| NATPARA | PARATHYROID HORMONE | 125511 | 10309921 | 3/3/2034 |
| NATPARA | PARATHYROID HORMONE | 125511 | 10317419 | 2/22/2032 |
| NATPARA | PARATHYROID HORMONE | 125511 | 10323083 | 1/15/2034 |
| NATPARA | PARATHYROID HORMONE | 125511 | 10328145 | 1/28/2030 |
| NATPARA | PARATHYROID HORMONE | 125511 | 10342764 | 2/1/2032 |
| NATPARA | PARATHYROID HORMONE | 125511 | 10342938 | 6/13/2028 |
| NATPARA | PARATHYROID HORMONE | 125511 | 10357459 | 9/14/2025 |
| NATPARA | PARATHYROID HORMONE | 125511 | 10364451 | 5/30/2033 |
| NATPARA | PARATHYROID HORMONE | 125511 | 10369161 | 12/30/2034 |
| NATPARA | PARATHYROID HORMONE | 125511 | 10376587 | 6/17/2031 |
| NATPARA | PARATHYROID HORMONE | 125511 | 10378055 | 4/8/2035 |
| NATPARA | PARATHYROID HORMONE | 125511 | 10385139 | 11/1/2033 |
| NATPARA | PARATHYROID HORMONE | 125511 | 10392413 | 12/18/2035 |
| NATPARA | PARATHYROID HORMONE | 125511 | 10399945 | 4/27/2037 |
| NATPARA | PARATHYROID HORMONE | 125511 | 10406202 | 10/22/2034 |
| NATPARA | PARATHYROID HORMONE | 125511 | 10421729 | 3/15/2033 |
| NATPARA | PARATHYROID HORMONE | 125511 | 10421954 | 5/20/2033 |
| NATPARA | PARATHYROID HORMONE | 125511 | 10436788 | 4/22/2035 |
| NATPARA | PARATHYROID HORMONE | 125511 | 10446261 | 1/6/2028 |
| NATPARA | PARATHYROID HORMONE | 125511 | 10456472 | 2/8/2027 |
| NATPARA | PARATHYROID HORMONE | 125511 | 10456478 | 3/21/2032 |
| NATPARA | PARATHYROID HORMONE | 125511 | 10466163 | 4/28/2031 |
| NATPARA | PARATHYROID HORMONE | 125511 | 10478396 | 12/23/2030 |
| NATPARA | PARATHYROID HORMONE | 125511 | 10479832 | 3/31/2034 |
| NATPARA | PARATHYROID HORMONE | 125511 | 10485902 | 8/28/2035 |
| NATPARA | PARATHYROID HORMONE | 125511 | 10487128 | 7/12/2033 |
| NATPARA | PARATHYROID HORMONE | 125511 | 10493084 | 8/7/2034 |
| NATPARA | PARATHYROID HORMONE | 125511 | 10493253 | 12/24/2029 |
| NATPARA | PARATHYROID HORMONE | 125511 | 10500159 | 11/2/2029 |
| NATPARA | PARATHYROID HORMONE | 125511 | 10500233 | 2/12/2034 |
| NATPARA | PARATHYROID HORMONE | 125511 | 10501518 | 6/23/2033 |
| NATPARA | PARATHYROID HORMONE | 125511 | 10507248 | 12/19/2025 |
| NATPARA | PARATHYROID HORMONE | 125511 | 10519250 | 8/1/2036 |
| NATPARA | PARATHYROID HORMONE | 125511 | 10525186 | 6/17/2029 |
| NATPARA | PARATHYROID HORMONE | 125511 | 10526657 | 12/10/2035 |
| NATPARA | PARATHYROID HORMONE | 125511 | 10538584 | 5/3/2025 |
| NATPARA | PARATHYROID HORMONE | 125511 | 10545158 | 12/29/2034 |
| NATPARA | PARATHYROID HORMONE | 125511 | 10549052 | 2/29/2040 |
| NATPARA | PARATHYROID HORMONE | 125511 | 10557862 | 6/5/2035 |
| NATPARA | PARATHYROID HORMONE | 125511 | 10577154 | 5/13/2029 |
| NATPARA | PARATHYROID HORMONE | 125511 | 10583177 | 2/9/2035 |
| NATPARA | PARATHYROID HORMONE | 125511 | 10588945 | 7/7/2035 |
| NATPARA | PARATHYROID HORMONE | 125511 | 10589001 | 3/16/2031 |
| NATPARA | PARATHYROID HORMONE | 125511 | 10591472 | 8/13/2032 |
| NATPARA | PARATHYROID HORMONE | 125511 | 10596359 | 12/24/2029 |
| NATPARA | PARATHYROID HORMONE | 125511 | 10603361 | 1/28/2035 |
| NATPARA | PARATHYROID HORMONE | 125511 | 10603383 | 6/17/2031 |
| NATPARA | PARATHYROID HORMONE | 125511 | 10603475 | 12/24/2029 |
| NATPARA | PARATHYROID HORMONE | 125511 | 10610649 | 3/11/2033 |
| NATPARA | PARATHYROID HORMONE | 125511 | 10611832 | 5/29/2029 |
| NATPARA | PARATHYROID HORMONE | 125511 | 10613101 | 3/8/2033 |
| NATPARA | PARATHYROID HORMONE | 125511 | 10618953 | 8/30/2033 |
| NATPARA | PARATHYROID HORMONE | 125511 | 10625034 | 4/1/2031 |
| NATPARA | PARATHYROID HORMONE | 125511 | 10654888 | 9/11/2022 |
| NATPARA | PARATHYROID HORMONE | 125511 | 10660940 | 3/5/2033 |
| NATPARA | PARATHYROID HORMONE | 125511 | 10662222 | 6/12/2034 |
| NATPARA | PARATHYROID HORMONE | 125511 | 10662231 | 3/23/2032 |
| NATPARA | PARATHYROID HORMONE | 125511 | 10669239 | 4/27/2037 |
| NATPARA | PARATHYROID HORMONE | 125511 | 4179337 | 4/30/2036 |
| NATPARA | PARATHYROID HORMONE | 125511 | 4341755 | 4/30/2036 |
| NATPARA | PARATHYROID HORMONE | 125511 | 4423037 | 4/30/2036 |
| NATPARA | PARATHYROID HORMONE | 125511 | 4427827 | 4/30/2036 |
| NATPARA | PARATHYROID HORMONE | 125511 | 4469787 | 4/30/2036 |
| NATPARA | PARATHYROID HORMONE | 125511 | 4508828 | 4/30/2036 |
| NATPARA | PARATHYROID HORMONE | 125511 | 4588716 | 5/4/2004 |
| NATPARA | PARATHYROID HORMONE | 125511 | 4621053 | 7/30/2000 |
| NATPARA | PARATHYROID HORMONE | 125511 | 4659696 | 4/21/2004 |
| NATPARA | PARATHYROID HORMONE | 125511 | 4670419 | 6/2/2004 |
| NATPARA | PARATHYROID HORMONE | 125511 | 4686104 | 4/30/2036 |
| NATPARA | PARATHYROID HORMONE | 125511 | 4692433 | 9/8/2004 |
| NATPARA | PARATHYROID HORMONE | 125511 | 4698328 | 4/4/2005 |
| NATPARA | PARATHYROID HORMONE | 125511 | 4746508 | 4/30/2036 |
| NATPARA | PARATHYROID HORMONE | 125511 | 4788178 | 11/29/2005 |
| NATPARA | PARATHYROID HORMONE | 125511 | 4812304 | 4/30/2004 |
| NATPARA | PARATHYROID HORMONE | 125511 | 4822609 | 4/18/2006 |
| NATPARA | PARATHYROID HORMONE | 125511 | 4833125 | 4/30/2036 |
| NATPARA | PARATHYROID HORMONE | 125511 | 4851329 | 4/30/2036 |
| NATPARA | PARATHYROID HORMONE | 125511 | 4864020 | 4/30/2036 |
| NATPARA | PARATHYROID HORMONE | 125511 | 4910021 | 3/20/2007 |
| NATPARA | PARATHYROID HORMONE | 125511 | 4935339 | 4/30/2036 |
| NATPARA | PARATHYROID HORMONE | 125511 | 4962091 | 4/30/2036 |
| NATPARA | PARATHYROID HORMONE | 125511 | 5010010 | 4/30/2036 |
| NATPARA | PARATHYROID HORMONE | 125511 | 5011678 | 4/30/2036 |
| NATPARA | PARATHYROID HORMONE | 125511 | 5028439 | 7/2/2008 |
| NATPARA | PARATHYROID HORMONE | 125511 | 5039660 | 4/30/2036 |
| NATPARA | PARATHYROID HORMONE | 125511 | 5053491 | 4/30/2036 |
| NATPARA | PARATHYROID HORMONE | 125511 | 5066436 | 1/4/2009 |
| NATPARA | PARATHYROID HORMONE | 125511 | 5077389 | 12/31/2008 |
| NATPARA | PARATHYROID HORMONE | 125511 | 5100788 | 3/31/2009 |
| NATPARA | PARATHYROID HORMONE | 125511 | 5116952 | 5/26/2009 |
| NATPARA | PARATHYROID HORMONE | 125511 | 5118667 | 4/30/2036 |
| NATPARA | PARATHYROID HORMONE | 125511 | 5137669 | 8/11/2009 |
| NATPARA | PARATHYROID HORMONE | 125511 | 5169933 | 12/8/2009 |
| NATPARA | PARATHYROID HORMONE | 125511 | 5171670 | 4/30/2036 |
| NATPARA | PARATHYROID HORMONE | 125511 | 5208041 | 4/30/2036 |
| NATPARA | PARATHYROID HORMONE | 125511 | 5217896 | 4/30/2036 |
| NATPARA | PARATHYROID HORMONE | 125511 | 5223408 | 4/30/2036 |
| NATPARA | PARATHYROID HORMONE | 125511 | 5288497 | 5/1/2005 |
| NATPARA | PARATHYROID HORMONE | 125511 | 5288498 | 6/8/2007 |
| NATPARA | PARATHYROID HORMONE | 125511 | 5317010 | 4/30/2036 |
| NATPARA | PARATHYROID HORMONE | 125511 | 5317017 | 4/30/2036 |
| NATPARA | PARATHYROID HORMONE | 125511 | 5350741 | 9/27/2011 |
| NATPARA | PARATHYROID HORMONE | 125511 | 5354654 | 4/30/2036 |
| NATPARA | PARATHYROID HORMONE | 125511 | 5355304 | 4/30/2036 |
| NATPARA | PARATHYROID HORMONE | 125511 | 5359030 | 4/30/2036 |
| NATPARA | PARATHYROID HORMONE | 125511 | 5366859 | 11/22/2011 |
| NATPARA | PARATHYROID HORMONE | 125511 | 5382658 | 4/30/2036 |
| NATPARA | PARATHYROID HORMONE | 125511 | 5385825 | 4/30/2036 |
| NATPARA | PARATHYROID HORMONE | 125511 | 5407911 | 4/18/2012 |
| NATPARA | PARATHYROID HORMONE | 125511 | 5420242 | 9/8/2009 |
| NATPARA | PARATHYROID HORMONE | 125511 | 5425764 | 7/30/2012 |
| NATPARA | PARATHYROID HORMONE | 125511 | 5437838 | 4/30/2036 |
| NATPARA | PARATHYROID HORMONE | 125511 | 5457092 | 10/10/2012 |
| NATPARA | PARATHYROID HORMONE | 125511 | 5460978 | 10/24/2012 |
| NERLYNX | NERATINIB MALEATE | 208051 | 10035788 | 10/15/2028 |
| NUBEQA | DAROLUTAMIDE | 212099 | 10010530 | 1/28/2036 |
| NUBEQA | DAROLUTAMIDE | 212099 | 10383853 | 1/28/2036 |
| NUCALA | MEPOLIZUMAB | 125526 | 10028940 | 8/13/2033 |
| NUCALA | MEPOLIZUMAB | 761122 | 10028940 | 8/13/2033 |
| NUCALA | MEPOLIZUMAB | 125526 | 10047033 | 5/13/2035 |
| NUCALA | MEPOLIZUMAB | 761122 | 10047033 | 5/13/2035 |
| NUCALA | MEPOLIZUMAB | 125526 | 10111968 | 2/15/2032 |
| NUCALA | MEPOLIZUMAB | 761122 | 10111968 | 2/15/2032 |
| NUCALA | MEPOLIZUMAB | 125526 | 10195183 | 8/13/2033 |
| NUCALA | MEPOLIZUMAB | 761122 | 10195183 | 8/13/2033 |
| NUCALA | MEPOLIZUMAB | 125526 | 10195211 | 6/15/2035 |
| NUCALA | MEPOLIZUMAB | 761122 | 10195211 | 6/15/2035 |
| NUCALA | MEPOLIZUMAB | 125526 | 10231976 | 2/8/2030 |
| NUCALA | MEPOLIZUMAB | 761122 | 10231976 | 2/8/2030 |
| NUCALA | MEPOLIZUMAB | 125526 | 10273252 | 4/28/2036 |
| NUCALA | MEPOLIZUMAB | 761122 | 10273252 | 4/28/2036 |
| NUCALA | MEPOLIZUMAB | 125526 | 10316282 | 3/19/2033 |
| NUCALA | MEPOLIZUMAB | 761122 | 10316282 | 3/19/2033 |
| NUCALA | MEPOLIZUMAB | 125526 | 10329314 | 4/4/2036 |
| NUCALA | MEPOLIZUMAB | 761122 | 10329314 | 4/4/2036 |
| NUCALA | MEPOLIZUMAB | 125526 | 10363235 | 12/2/2034 |
| NUCALA | MEPOLIZUMAB | 761122 | 10363235 | 12/2/2034 |
| NUCALA | MEPOLIZUMAB | 125526 | 10369114 | 3/14/2033 |
| NUCALA | MEPOLIZUMAB | 761122 | 10369114 | 3/14/2033 |
| NUCALA | MEPOLIZUMAB | 125526 | 10435452 | 5/29/2035 |
| NUCALA | MEPOLIZUMAB | 761122 | 10435452 | 5/29/2035 |
| NUCALA | MEPOLIZUMAB | 125526 | 10456381 | 8/13/2033 |
| NUCALA | MEPOLIZUMAB | 761122 | 10456381 | 8/13/2033 |
| NUCALA | MEPOLIZUMAB | 125526 | 10487098 | 4/4/2036 |
| NUCALA | MEPOLIZUMAB | 761122 | 10487098 | 4/4/2036 |
| NUCALA | MEPOLIZUMAB | 125526 | 10507262 | 5/11/2032 |
| NUCALA | MEPOLIZUMAB | 761122 | 10507262 | 5/11/2032 |
| NUCALA | MEPOLIZUMAB | 125526 | 10562974 | 3/13/2033 |
| NUCALA | MEPOLIZUMAB | 761122 | 10562974 | 3/13/2033 |
| NUCALA | MEPOLIZUMAB | 125526 | 10584180 | 3/26/2040 |
| NUCALA | MEPOLIZUMAB | 761122 | 10584180 | 3/26/2040 |
| NUCALA | MEPOLIZUMAB | 125526 | 10590182 | 2/24/2035 |
| NUCALA | MEPOLIZUMAB | 761122 | 10590182 | 2/24/2035 |
| NUCALA | MEPOLIZUMAB | 125526 | 10640558 | 9/23/2034 |
| NUCALA | MEPOLIZUMAB | 761122 | 10640558 | 9/23/2034 |
| NULOJIX | BELATACEPT | 125288 | 10279014 | 5/17/2039 |
| NULOJIX | BELATACEPT | 125288 | 10308985 | 6/26/2034 |
| NULOJIX | BELATACEPT | 125288 | 10329314 | 4/4/2036 |
| NULOJIX | BELATACEPT | 125288 | 10487098 | 4/4/2036 |
| NULOJIX | BELATACEPT | 125288 | 10488413 | 3/28/2033 |
| NULOJIX | BELATACEPT | 125288 | 10507262 | 5/11/2032 |
| NULOJIX | BELATACEPT | 125288 | 10532098 | 1/24/2040 |
| NUPLAZID | PIMAVANSERIN TARTRATE | 207318 | 10028944 | 1/15/2024 |
| NUPLAZID | PIMAVANSERIN TARTRATE | 210793 | 10028944 | 1/15/2024 |
| NUPLAZID | PIMAVANSERIN TARTRATE | 210793 | 10449185 | 8/27/2038 |
| NUPLAZID | PIMAVANSERIN TARTRATE | 207318 | 10517860 | 3/23/2037 |
| NUPLAZID | PIMAVANSERIN TARTRATE | 210793 | 10646480 | 8/27/2038 |
| OCALIVA | OBETICHOLIC ACID | 207999 | 10047117 | 9/6/2033 |
| OCALIVA | OBETICHOLIC ACID | 207999 | 10052337 | 4/26/2036 |
| OCALIVA | OBETICHOLIC ACID | 207999 | 10174073 | 6/17/2033 |
| OCREVUS | OCRELIZUMAB | 761053 | 10016338 | 3/11/2033 |
| OCREVUS | OCRELIZUMAB | 761053 | 10035848 | 1/8/2034 |
| OCREVUS | OCRELIZUMAB | 761053 | 10093732 | 11/25/2028 |
| OCREVUS | OCRELIZUMAB | 761053 | 10111968 | 2/15/2032 |
| OCREVUS | OCRELIZUMAB | 761053 | 10233503 | 3/23/2039 |
| OCREVUS | OCRELIZUMAB | 761053 | 10251906 | 3/12/2033 |
| OCREVUS | OCRELIZUMAB | 761053 | 10273252 | 4/28/2036 |
| OCREVUS | OCRELIZUMAB | 761053 | 10329314 | 4/4/2036 |
| OCREVUS | OCRELIZUMAB | 761053 | 10369114 | 3/14/2033 |
| OCREVUS | OCRELIZUMAB | 761053 | 10407673 | 8/24/2036 |
| OCREVUS | OCRELIZUMAB | 761053 | 10416159 | 7/17/2034 |
| OCREVUS | OCRELIZUMAB | 761053 | 10450365 | 12/31/2032 |
| OCREVUS | OCRELIZUMAB | 761053 | 10487098 | 4/4/2036 |
| OCREVUS | OCRELIZUMAB | 761053 | 10493113 | 11/14/2037 |
| OCREVUS | OCRELIZUMAB | 761053 | 10577154 | 5/13/2029 |
| OCREVUS | OCRELIZUMAB | 761053 | 10584180 | 3/26/2040 |
| OCREVUS | OCRELIZUMAB | 761053 | 10590182 | 2/24/2035 |
| OCREVUS | OCRELIZUMAB | 761053 | 10668053 | 5/3/2033 |
| OFEV | NINTEDANIB ESYLATE | 205832 | 10105323 | 6/4/2029 |
| OFEV | NINTEDANIB ESYLATE | 205832 | 10154990 | 12/20/2025 |
| OPDIVO | NIVOLUMAB | 125527 | 10001483 | 6/26/2035 |
| OPDIVO | NIVOLUMAB | 125554 | 10001483 | 6/26/2035 |
| OPDIVO | NIVOLUMAB | 125527 | 10004812 | 2/6/2034 |
| OPDIVO | NIVOLUMAB | 125554 | 10004812 | 2/6/2034 |
| OPDIVO | NIVOLUMAB | 125527 | 10007766 | 7/13/2035 |
| OPDIVO | NIVOLUMAB | 125554 | 10007766 | 7/13/2035 |
| OPDIVO | NIVOLUMAB | 125527 | 10017492 | 10/30/2034 |
| OPDIVO | NIVOLUMAB | 125554 | 10017492 | 10/30/2034 |
| OPDIVO | NIVOLUMAB | 125527 | 10034938 | 8/30/2032 |
| OPDIVO | NIVOLUMAB | 125554 | 10034938 | 8/30/2032 |
| OPDIVO | NIVOLUMAB | 125527 | 10072082 | 5/15/2032 |
| OPDIVO | NIVOLUMAB | 125554 | 10072082 | 5/15/2032 |
| OPDIVO | NIVOLUMAB | 125527 | 10092645 | 6/17/2034 |
| OPDIVO | NIVOLUMAB | 125554 | 10092645 | 6/17/2034 |
| OPDIVO | NIVOLUMAB | 125527 | 10100034 | 5/4/2035 |
| OPDIVO | NIVOLUMAB | 125554 | 10100034 | 5/4/2035 |
| OPDIVO | NIVOLUMAB | 125527 | 10106546 | 11/5/2034 |
| OPDIVO | NIVOLUMAB | 125554 | 10106546 | 11/5/2034 |
| OPDIVO | NIVOLUMAB | 125527 | 10111900 | 11/6/2034 |
| OPDIVO | NIVOLUMAB | 125554 | 10111900 | 11/6/2034 |
| OPDIVO | NIVOLUMAB | 125527 | 10111954 | 12/4/2032 |
| OPDIVO | NIVOLUMAB | 125554 | 10111954 | 12/4/2032 |
| OPDIVO | NIVOLUMAB | 125527 | 10112941 | 12/24/2033 |
| OPDIVO | NIVOLUMAB | 125554 | 10112941 | 12/24/2033 |
| OPDIVO | NIVOLUMAB | 125527 | 10130718 | 1/7/2033 |
| OPDIVO | NIVOLUMAB | 125554 | 10130718 | 1/7/2033 |
| OPDIVO | NIVOLUMAB | 125527 | 10137202 | 2/6/2034 |
| OPDIVO | NIVOLUMAB | 125554 | 10137202 | 2/6/2034 |
| OPDIVO | NIVOLUMAB | 125527 | 10143723 | 12/23/2035 |
| OPDIVO | NIVOLUMAB | 125554 | 10143723 | 12/23/2035 |
| OPDIVO | NIVOLUMAB | 125527 | 10144779 | 5/29/2035 |
| OPDIVO | NIVOLUMAB | 125554 | 10144779 | 5/29/2035 |
| OPDIVO | NIVOLUMAB | 125527 | 10160747 | 3/16/2036 |
| OPDIVO | NIVOLUMAB | 125554 | 10160747 | 3/16/2036 |
| OPDIVO | NIVOLUMAB | 125527 | 10166290 | 1/22/2039 |
| OPDIVO | NIVOLUMAB | 125554 | 10166290 | 1/22/2039 |
| OPDIVO | NIVOLUMAB | 125527 | 10167254 | 4/3/2035 |
| OPDIVO | NIVOLUMAB | 125554 | 10167254 | 4/3/2035 |
| OPDIVO | NIVOLUMAB | 125527 | 10172808 | 5/18/2036 |
| OPDIVO | NIVOLUMAB | 125554 | 10172808 | 5/18/2036 |
| OPDIVO | NIVOLUMAB | 125527 | 10174113 | 4/28/2035 |
| OPDIVO | NIVOLUMAB | 125554 | 10174113 | 4/28/2035 |
| OPDIVO | NIVOLUMAB | 125527 | 10189797 | 12/30/2035 |
| OPDIVO | NIVOLUMAB | 125554 | 10189797 | 12/30/2035 |
| OPDIVO | NIVOLUMAB | 125527 | 10189822 | 3/19/2035 |
| OPDIVO | NIVOLUMAB | 125554 | 10189822 | 3/19/2035 |
| OPDIVO | NIVOLUMAB | 125527 | 10195175 | 6/25/2035 |
| OPDIVO | NIVOLUMAB | 125554 | 10195175 | 6/25/2035 |
| OPDIVO | NIVOLUMAB | 125527 | 10195188 | 6/13/2036 |
| OPDIVO | NIVOLUMAB | 125554 | 10195188 | 6/13/2036 |
| OPDIVO | NIVOLUMAB | 125527 | 10195273 | 6/5/2036 |
| OPDIVO | NIVOLUMAB | 125554 | 10195273 | 6/5/2036 |
| OPDIVO | NIVOLUMAB | 125527 | 10206893 | 11/5/2034 |
| OPDIVO | NIVOLUMAB | 125554 | 10206893 | 11/5/2034 |
| OPDIVO | NIVOLUMAB | 125527 | 10220072 | 6/30/2036 |
| OPDIVO | NIVOLUMAB | 125554 | 10220072 | 6/30/2036 |
| OPDIVO | NIVOLUMAB | 125527 | 10221244 | 10/29/2034 |
| OPDIVO | NIVOLUMAB | 125554 | 10221244 | 10/29/2034 |
| OPDIVO | NIVOLUMAB | 125527 | 10227328 | 2/4/2035 |
| OPDIVO | NIVOLUMAB | 125554 | 10227328 | 2/4/2035 |
| OPDIVO | NIVOLUMAB | 125527 | 10239912 | 1/8/2036 |
| OPDIVO | NIVOLUMAB | 125554 | 10239912 | 1/8/2036 |
| OPDIVO | NIVOLUMAB | 125527 | 10241115 | 3/26/2039 |
| OPDIVO | NIVOLUMAB | 125554 | 10241115 | 3/26/2039 |
| OPDIVO | NIVOLUMAB | 125527 | 10258619 | 10/5/2035 |
| OPDIVO | NIVOLUMAB | 125554 | 10258619 | 10/5/2035 |
| OPDIVO | NIVOLUMAB | 125527 | 10259859 | 8/7/2035 |
| OPDIVO | NIVOLUMAB | 125554 | 10259859 | 8/7/2035 |
| OPDIVO | NIVOLUMAB | 125527 | 10265291 | 7/15/2035 |
| OPDIVO | NIVOLUMAB | 125554 | 10265291 | 7/15/2035 |
| OPDIVO | NIVOLUMAB | 125527 | 10266591 | 7/2/2032 |
| OPDIVO | NIVOLUMAB | 125554 | 10266591 | 7/2/2032 |
| OPDIVO | NIVOLUMAB | 125527 | 10266605 | 4/27/2036 |
| OPDIVO | NIVOLUMAB | 125554 | 10266605 | 4/27/2036 |
| OPDIVO | NIVOLUMAB | 125527 | 10272113 | 3/29/2037 |
| OPDIVO | NIVOLUMAB | 125554 | 10272113 | 3/29/2037 |
| OPDIVO | NIVOLUMAB | 125527 | 10273252 | 4/28/2036 |
| OPDIVO | NIVOLUMAB | 125554 | 10273252 | 4/28/2036 |
| OPDIVO | NIVOLUMAB | 125527 | 10278954 | 2/1/2036 |
| OPDIVO | NIVOLUMAB | 125554 | 10278954 | 2/1/2036 |
| OPDIVO | NIVOLUMAB | 125527 | 10278984 | 5/25/2039 |
| OPDIVO | NIVOLUMAB | 125554 | 10278984 | 5/25/2039 |
| OPDIVO | NIVOLUMAB | 125527 | 10287353 | 5/11/2036 |
| OPDIVO | NIVOLUMAB | 125554 | 10287353 | 5/11/2036 |
| OPDIVO | NIVOLUMAB | 125527 | 10292951 | 9/1/2035 |
| OPDIVO | NIVOLUMAB | 125554 | 10292951 | 9/1/2035 |
| OPDIVO | NIVOLUMAB | 125527 | 10300138 | 6/5/2036 |
| OPDIVO | NIVOLUMAB | 125554 | 10300138 | 6/5/2036 |
| OPDIVO | NIVOLUMAB | 125527 | 10300139 | 6/5/2036 |
| OPDIVO | NIVOLUMAB | 125554 | 10300139 | 6/5/2036 |
| OPDIVO | NIVOLUMAB | 125527 | 10314855 | 1/23/2037 |
| OPDIVO | NIVOLUMAB | 125554 | 10314855 | 1/23/2037 |
| OPDIVO | NIVOLUMAB | 125527 | 10314910 | 1/10/2036 |
| OPDIVO | NIVOLUMAB | 125554 | 10314910 | 1/10/2036 |
| OPDIVO | NIVOLUMAB | 125527 | 10323004 | 5/4/2036 |
| OPDIVO | NIVOLUMAB | 125554 | 10323004 | 5/4/2036 |
| OPDIVO | NIVOLUMAB | 125527 | 10328157 | 7/15/2035 |
| OPDIVO | NIVOLUMAB | 125554 | 10328157 | 7/15/2035 |
| OPDIVO | NIVOLUMAB | 125527 | 10328158 | 1/10/2034 |
| OPDIVO | NIVOLUMAB | 125554 | 10328158 | 1/10/2034 |
| OPDIVO | NIVOLUMAB | 125527 | 10335388 | 4/17/2035 |
| OPDIVO | NIVOLUMAB | 125554 | 10335388 | 4/17/2035 |
| OPDIVO | NIVOLUMAB | 125527 | 10335486 | 5/18/2036 |
| OPDIVO | NIVOLUMAB | 125554 | 10335486 | 5/18/2036 |
| OPDIVO | NIVOLUMAB | 125527 | 10342790 | 2/20/2037 |
| OPDIVO | NIVOLUMAB | 125554 | 10342790 | 2/20/2037 |
| OPDIVO | NIVOLUMAB | 125527 | 10342866 | 4/29/2035 |
| OPDIVO | NIVOLUMAB | 125554 | 10342866 | 4/29/2035 |
| OPDIVO | NIVOLUMAB | 125527 | 10350213 | 7/30/2039 |
| OPDIVO | NIVOLUMAB | 125554 | 10350213 | 7/30/2039 |
| OPDIVO | NIVOLUMAB | 125527 | 10351625 | 2/19/2035 |
| OPDIVO | NIVOLUMAB | 125554 | 10351625 | 2/19/2035 |
| OPDIVO | NIVOLUMAB | 125527 | 10351627 | 7/27/2039 |
| OPDIVO | NIVOLUMAB | 125554 | 10351627 | 7/27/2039 |
| OPDIVO | NIVOLUMAB | 125527 | 10358496 | 3/1/2036 |
| OPDIVO | NIVOLUMAB | 125554 | 10358496 | 3/1/2036 |
| OPDIVO | NIVOLUMAB | 125527 | 10363308 | 6/5/2036 |
| OPDIVO | NIVOLUMAB | 125554 | 10363308 | 6/5/2036 |
| OPDIVO | NIVOLUMAB | 125527 | 10369114 | 3/14/2033 |
| OPDIVO | NIVOLUMAB | 125554 | 10369114 | 3/14/2033 |
| OPDIVO | NIVOLUMAB | 125527 | 10370346 | 8/6/2033 |
| OPDIVO | NIVOLUMAB | 125554 | 10370346 | 8/6/2033 |
| OPDIVO | NIVOLUMAB | 125527 | 10370374 | 9/21/2035 |
| OPDIVO | NIVOLUMAB | 125554 | 10370374 | 9/21/2035 |
| OPDIVO | NIVOLUMAB | 125527 | 10377824 | 7/2/2032 |
| OPDIVO | NIVOLUMAB | 125554 | 10377824 | 7/2/2032 |
| OPDIVO | NIVOLUMAB | 125527 | 10385130 | 5/11/2036 |
| OPDIVO | NIVOLUMAB | 125554 | 10385130 | 5/11/2036 |
| OPDIVO | NIVOLUMAB | 125527 | 10392442 | 12/17/2035 |
| OPDIVO | NIVOLUMAB | 125554 | 10392442 | 12/17/2035 |
| OPDIVO | NIVOLUMAB | 125527 | 10398685 | 6/13/2036 |
| OPDIVO | NIVOLUMAB | 125554 | 10398685 | 6/13/2036 |
| OPDIVO | NIVOLUMAB | 125527 | 10406278 | 2/11/2036 |
| OPDIVO | NIVOLUMAB | 125554 | 10406278 | 2/11/2036 |
| OPDIVO | NIVOLUMAB | 125527 | 10426847 | 3/26/2035 |
| OPDIVO | NIVOLUMAB | 125554 | 10426847 | 3/26/2035 |
| OPDIVO | NIVOLUMAB | 125527 | 10426972 | 3/30/2037 |
| OPDIVO | NIVOLUMAB | 125554 | 10426972 | 3/30/2037 |
| OPDIVO | NIVOLUMAB | 125527 | 10428067 | 6/7/2037 |
| OPDIVO | NIVOLUMAB | 125554 | 10428067 | 6/7/2037 |
| OPDIVO | NIVOLUMAB | 125527 | 10428143 | 9/28/2034 |
| OPDIVO | NIVOLUMAB | 125554 | 10428143 | 9/28/2034 |
| OPDIVO | NIVOLUMAB | 125527 | 10434082 | 7/26/2032 |
| OPDIVO | NIVOLUMAB | 125554 | 10434082 | 7/26/2032 |
| OPDIVO | NIVOLUMAB | 125527 | 10463686 | 9/15/2036 |
| OPDIVO | NIVOLUMAB | 125554 | 10463686 | 9/15/2036 |
| OPDIVO | NIVOLUMAB | 125527 | 10485796 | 6/29/2035 |
| OPDIVO | NIVOLUMAB | 125554 | 10485796 | 6/29/2035 |
| OPDIVO | NIVOLUMAB | 125527 | 10492723 | 2/27/2037 |
| OPDIVO | NIVOLUMAB | 125554 | 10492723 | 2/27/2037 |
| OPDIVO | NIVOLUMAB | 125527 | 10507244 | 10/1/2035 |
| OPDIVO | NIVOLUMAB | 125554 | 10507244 | 10/1/2035 |
| OPDIVO | NIVOLUMAB | 125527 | 10508085 | 9/22/2036 |
| OPDIVO | NIVOLUMAB | 125554 | 10508085 | 9/22/2036 |
| OPDIVO | NIVOLUMAB | 125527 | 10512662 | 2/25/2036 |
| OPDIVO | NIVOLUMAB | 125554 | 10512662 | 2/25/2036 |
| OPDIVO | NIVOLUMAB | 125527 | 10512689 | 4/17/2035 |
| OPDIVO | NIVOLUMAB | 125554 | 10512689 | 4/17/2035 |
| OPDIVO | NIVOLUMAB | 125527 | 10533014 | 11/5/2034 |
| OPDIVO | NIVOLUMAB | 125554 | 10533014 | 11/5/2034 |
| OPDIVO | NIVOLUMAB | 125527 | 10544099 | 5/4/2036 |
| OPDIVO | NIVOLUMAB | 125554 | 10544099 | 5/4/2036 |
| OPDIVO | NIVOLUMAB | 125527 | 10544125 | 5/4/2035 |
| OPDIVO | NIVOLUMAB | 125554 | 10544125 | 5/4/2035 |
| OPDIVO | NIVOLUMAB | 125527 | 10544223 | 4/20/2037 |
| OPDIVO | NIVOLUMAB | 125554 | 10544223 | 4/20/2037 |
| OPDIVO | NIVOLUMAB | 125527 | 10544224 | 7/14/2035 |
| OPDIVO | NIVOLUMAB | 125554 | 10544224 | 7/14/2035 |
| OPDIVO | NIVOLUMAB | 125527 | 10548988 | 7/18/2032 |
| OPDIVO | NIVOLUMAB | 125554 | 10548988 | 7/18/2032 |
| OPDIVO | NIVOLUMAB | 125527 | 10555976 | 2/4/2031 |
| OPDIVO | NIVOLUMAB | 125554 | 10555976 | 2/4/2031 |
| OPDIVO | NIVOLUMAB | 125527 | 10555981 | 7/16/2034 |
| OPDIVO | NIVOLUMAB | 125554 | 10555981 | 7/16/2034 |
| OPDIVO | NIVOLUMAB | 125527 | 10556957 | 2/29/2040 |
| OPDIVO | NIVOLUMAB | 125554 | 10556957 | 2/29/2040 |
| OPDIVO | NIVOLUMAB | 125527 | 10561632 | 7/26/2032 |
| OPDIVO | NIVOLUMAB | 125554 | 10561632 | 7/26/2032 |
| OPDIVO | NIVOLUMAB | 125527 | 10561653 | 8/11/2035 |
| OPDIVO | NIVOLUMAB | 125554 | 10561653 | 8/11/2035 |
| OPDIVO | NIVOLUMAB | 125527 | 10576111 | 7/5/2037 |
| OPDIVO | NIVOLUMAB | 125554 | 10576111 | 7/5/2037 |
| OPDIVO | NIVOLUMAB | 125527 | 10576136 | 12/23/2036 |
| OPDIVO | NIVOLUMAB | 125554 | 10576136 | 12/23/2036 |
| OPDIVO | NIVOLUMAB | 125527 | 10583131 | 6/13/2036 |
| OPDIVO | NIVOLUMAB | 125554 | 10583131 | 6/13/2036 |
| OPDIVO | NIVOLUMAB | 125527 | 10584169 | 1/24/2037 |
| OPDIVO | NIVOLUMAB | 125554 | 10584169 | 1/24/2037 |
| OPDIVO | NIVOLUMAB | 125527 | 10590182 | 2/24/2035 |
| OPDIVO | NIVOLUMAB | 125554 | 10590182 | 2/24/2035 |
| OPDIVO | NIVOLUMAB | 125527 | 10596112 | 10/6/2034 |
| OPDIVO | NIVOLUMAB | 125554 | 10596112 | 10/6/2034 |
| OPDIVO | NIVOLUMAB | 125527 | 10597411 | 4/1/2040 |
| OPDIVO | NIVOLUMAB | 125554 | 10597411 | 4/1/2040 |
| OPDIVO | NIVOLUMAB | 125527 | 10603379 | 6/5/2036 |
| OPDIVO | NIVOLUMAB | 125554 | 10603379 | 6/5/2036 |
| OPDIVO | NIVOLUMAB | 125527 | 10604542 | 1/11/2036 |
| OPDIVO | NIVOLUMAB | 125554 | 10604542 | 1/11/2036 |
| OPDIVO | NIVOLUMAB | 125527 | 10611754 | 2/4/2035 |
| OPDIVO | NIVOLUMAB | 125554 | 10611754 | 2/4/2035 |
| OPDIVO | NIVOLUMAB | 125527 | 10613092 | 4/1/2036 |
| OPDIVO | NIVOLUMAB | 125554 | 10613092 | 4/1/2036 |
| OPDIVO | NIVOLUMAB | 125527 | 10617667 | 11/1/2037 |
| OPDIVO | NIVOLUMAB | 125554 | 10617667 | 11/1/2037 |
| OPDIVO | NIVOLUMAB | 125527 | 10617758 | 10/9/2035 |
| OPDIVO | NIVOLUMAB | 125554 | 10617758 | 10/9/2035 |
| OPDIVO | NIVOLUMAB | 125527 | 10618955 | 7/15/2034 |
| OPDIVO | NIVOLUMAB | 125554 | 10618955 | 7/15/2034 |
| OPDIVO | NIVOLUMAB | 125527 | 10618958 | 8/19/2034 |
| OPDIVO | NIVOLUMAB | 125554 | 10618958 | 8/19/2034 |
| OPDIVO | NIVOLUMAB | 125527 | 10618967 | 10/29/2034 |
| OPDIVO | NIVOLUMAB | 125554 | 10618967 | 10/29/2034 |
| OPDIVO | NIVOLUMAB | 125527 | 10632193 | 12/24/2034 |
| OPDIVO | NIVOLUMAB | 125554 | 10632193 | 12/24/2034 |
| OPDIVO | NIVOLUMAB | 125527 | 10633342 | 5/4/2036 |
| OPDIVO | NIVOLUMAB | 125554 | 10633342 | 5/4/2036 |
| OPDIVO | NIVOLUMAB | 125527 | 10646517 | 3/29/2037 |
| OPDIVO | NIVOLUMAB | 125554 | 10646517 | 3/29/2037 |
| OPDIVO | NIVOLUMAB | 125527 | 10653793 | 12/13/2032 |
| OPDIVO | NIVOLUMAB | 125554 | 10653793 | 12/13/2032 |
| OPDIVO | NIVOLUMAB | 125527 | 10660891 | 6/8/2038 |
| OPDIVO | NIVOLUMAB | 125554 | 10660891 | 6/8/2038 |
| OPDIVO | NIVOLUMAB | 125527 | 10660909 | 6/4/2040 |
| OPDIVO | NIVOLUMAB | 125554 | 10660909 | 6/4/2040 |
| OPDIVO | NIVOLUMAB | 125527 | 10660971 | 7/18/2032 |
| OPDIVO | NIVOLUMAB | 125554 | 10660971 | 7/18/2032 |
| OPDIVO | NIVOLUMAB | 125527 | 10662252 | 8/14/2032 |
| OPDIVO | NIVOLUMAB | 125554 | 10662252 | 8/14/2032 |
| OPDIVO | NIVOLUMAB | 125527 | 10668152 | 12/17/2035 |
| OPDIVO | NIVOLUMAB | 125554 | 10668152 | 12/17/2035 |
| OPDIVO | NIVOLUMAB | 125527 | 10669337 | 7/25/2034 |
| OPDIVO | NIVOLUMAB | 125554 | 10669337 | 7/25/2034 |
| OPDIVO | NIVOLUMAB | 125527 | 10669338 | 6/17/2036 |
| OPDIVO | NIVOLUMAB | 125554 | 10669338 | 6/17/2036 |
| ORILISSA | ELAGOLIX SODIUM | 210450 | 10537572 | 9/1/2036 |
| ORKAMBI | IVACAFTOR; LUMACAFTOR | 206038 | 10076513 | 12/4/2028 |
| ORKAMBI | IVACAFTOR; LUMACAFTOR | 206038 | 10597384 | 12/4/2028 |
| ORKAMBI | IVACAFTOR; LUMACAFTOR | 211358 | 10597384 | 12/4/2028 |
| OTEZLA | APREMILAST | 205437 | 10092541 | 5/29/2034 |
| OXBRYTA | VOXELOTOR | 213137 | 10017491 | 12/28/2032 |
| OXBRYTA | VOXELOTOR | 213137 | 10034879 | 12/28/2032 |
| OXBRYTA | VOXELOTOR | 213137 | 10493035 | 10/12/2037 |
| OZEMPIC | SEMAGLUTIDE | 209637 | 10220155 | 7/17/2026 |
| OZEMPIC | SEMAGLUTIDE | 209637 | 10335462 | 6/21/2033 |
| OZEMPIC | SEMAGLUTIDE | 209637 | 10357616 | 1/20/2026 |
| OZEMPIC | SEMAGLUTIDE | 209637 | 10376652 | 1/20/2026 |
| PARSABIV | ETELCALCETIDE | 208325 | 10344765 | 6/27/2034 |
| PERJETA | PERTUZUMAB | 125409 | 10005783 | 10/21/2029 |
| PERJETA | PERTUZUMAB | 125409 | 10016338 | 3/11/2033 |
| PERJETA | PERTUZUMAB | 125409 | 10016434 | 8/12/2033 |
| PERJETA | PERTUZUMAB | 125409 | 10022451 | 10/10/2038 |
| PERJETA | PERTUZUMAB | 125409 | 10040857 | 11/23/2031 |
| PERJETA | PERTUZUMAB | 125409 | 10041934 | 6/12/2032 |
| PERJETA | PERTUZUMAB | 125409 | 10047163 | 2/8/2033 |
| PERJETA | PERTUZUMAB | 125409 | 10058610 | 5/2/2032 |
| PERJETA | PERTUZUMAB | 125409 | 10085983 | 9/30/2034 |
| PERJETA | PERTUZUMAB | 125409 | 10087237 | 12/21/2030 |
| PERJETA | PERTUZUMAB | 125409 | 10092659 | 1/10/2034 |
| PERJETA | PERTUZUMAB | 125409 | 10093732 | 11/25/2028 |
| PERJETA | PERTUZUMAB | 125409 | 10111968 | 2/15/2032 |
| PERJETA | PERTUZUMAB | 125409 | 10143749 | 3/28/2033 |
| PERJETA | PERTUZUMAB | 125409 | 10155050 | 8/7/2035 |
| PERJETA | PERTUZUMAB | 125409 | 10160747 | 3/16/2036 |
| PERJETA | PERTUZUMAB | 125409 | 10160812 | 4/11/2034 |
| PERJETA | PERTUZUMAB | 125409 | 10195188 | 6/13/2036 |
| PERJETA | PERTUZUMAB | 125409 | 10208357 | 2/23/2039 |
| PERJETA | PERTUZUMAB | 125409 | 10220072 | 6/30/2036 |
| PERJETA | PERTUZUMAB | 125409 | 10227328 | 2/4/2035 |
| PERJETA | PERTUZUMAB | 125409 | 10239862 | 3/15/2037 |
| PERJETA | PERTUZUMAB | 125409 | 10246484 | 11/6/2033 |
| PERJETA | PERTUZUMAB | 125409 | 10253108 | 1/30/2035 |
| PERJETA | PERTUZUMAB | 125409 | 10259859 | 8/7/2035 |
| PERJETA | PERTUZUMAB | 125409 | 10261083 | 1/4/2033 |
| PERJETA | PERTUZUMAB | 125409 | 10265291 | 7/15/2035 |
| PERJETA | PERTUZUMAB | 125409 | 10272144 | 7/31/2033 |
| PERJETA | PERTUZUMAB | 125409 | 10273252 | 4/28/2036 |
| PERJETA | PERTUZUMAB | 125409 | 10273303 | 11/13/2033 |
| PERJETA | PERTUZUMAB | 125409 | 10287294 | 7/8/2035 |
| PERJETA | PERTUZUMAB | 125409 | 10301280 | 12/21/2032 |
| PERJETA | PERTUZUMAB | 125409 | 10314846 | 9/17/2034 |
| PERJETA | PERTUZUMAB | 125409 | 10314921 | 4/25/2034 |
| PERJETA | PERTUZUMAB | 125409 | 10316282 | 3/19/2033 |
| PERJETA | PERTUZUMAB | 125409 | 10328157 | 7/15/2035 |
| PERJETA | PERTUZUMAB | 125409 | 10328158 | 1/10/2034 |
| PERJETA | PERTUZUMAB | 125409 | 10342790 | 2/20/2037 |
| PERJETA | PERTUZUMAB | 125409 | 10370374 | 9/21/2035 |
| PERJETA | PERTUZUMAB | 125409 | 10370414 | 11/30/2032 |
| PERJETA | PERTUZUMAB | 125409 | 10391055 | 10/6/2034 |
| PERJETA | PERTUZUMAB | 125409 | 10398660 | 10/16/2034 |
| PERJETA | PERTUZUMAB | 125409 | 10398685 | 6/13/2036 |
| PERJETA | PERTUZUMAB | 125409 | 10407673 | 8/24/2036 |
| PERJETA | PERTUZUMAB | 125409 | 10413607 | 12/7/2035 |
| PERJETA | PERTUZUMAB | 125409 | 10428067 | 6/7/2037 |
| PERJETA | PERTUZUMAB | 125409 | 10434082 | 7/26/2032 |
| PERJETA | PERTUZUMAB | 125409 | 10435414 | 9/8/2035 |
| PERJETA | PERTUZUMAB | 125409 | 10456479 | 10/29/2039 |
| PERJETA | PERTUZUMAB | 125409 | 10487143 | 10/5/2036 |
| PERJETA | PERTUZUMAB | 125409 | 10508085 | 9/22/2036 |
| PERJETA | PERTUZUMAB | 125409 | 10512630 | 9/30/2035 |
| PERJETA | PERTUZUMAB | 125409 | 10519131 | 3/15/2037 |
| PERJETA | PERTUZUMAB | 125409 | 10557175 | 9/19/2034 |
| PERJETA | PERTUZUMAB | 125409 | 10561632 | 7/26/2032 |
| PERJETA | PERTUZUMAB | 125409 | 10562974 | 3/13/2033 |
| PERJETA | PERTUZUMAB | 125409 | 10562977 | 1/29/2034 |
| PERJETA | PERTUZUMAB | 125409 | 10577154 | 5/13/2029 |
| PERJETA | PERTUZUMAB | 125409 | 10583131 | 6/13/2036 |
| PERJETA | PERTUZUMAB | 125409 | 10584178 | 12/20/2033 |
| PERJETA | PERTUZUMAB | 125409 | 10584181 | 12/4/2029 |
| PERJETA | PERTUZUMAB | 125409 | 10590182 | 2/24/2035 |
| PERJETA | PERTUZUMAB | 125409 | 10596112 | 10/6/2034 |
| PERJETA | PERTUZUMAB | 125409 | 10603387 | 1/10/2034 |
| PERJETA | PERTUZUMAB | 125409 | 10611754 | 2/4/2035 |
| PERJETA | PERTUZUMAB | 125409 | 10618935 | 11/3/2035 |
| PERJETA | PERTUZUMAB | 125409 | 10618973 | 5/27/2034 |
| PERJETA | PERTUZUMAB | 125409 | 10624874 | 4/27/2040 |
| PERJETA | PERTUZUMAB | 125409 | 10624878 | 4/27/2040 |
| PERJETA | PERTUZUMAB | 125409 | 10632196 | 5/2/2040 |
| PERJETA | PERTUZUMAB | 125409 | 10639373 | 5/27/2040 |
| PERJETA | PERTUZUMAB | 125409 | 10669337 | 7/25/2034 |
| PLEGRIDY | PEGINTERFERON BETA-1A | 125499 | 10085955 | 1/8/2033 |
| PLEGRIDY | PEGINTERFERON BETA-1A | 125499 | 10213420 | 2/5/2034 |
| PLEGRIDY | PEGINTERFERON BETA-1A | 125499 | 10350178 | 1/8/2033 |
| PLEGRIDY | PEGINTERFERON BETA-1A | 125499 | 10632112 | 2/5/2034 |
| POMALYST | POMALIDOMIDE | 204026 | 10555939 | 5/19/2030 |
| PORTRAZZA | NECITUMUMAB | 125547 | 10047163 | 2/8/2033 |
| PORTRAZZA | NECITUMUMAB | 125547 | 10111968 | 2/15/2032 |
| PORTRAZZA | NECITUMUMAB | 125547 | 10189822 | 3/19/2035 |
| PORTRAZZA | NECITUMUMAB | 125547 | 10220072 | 6/30/2036 |
| PORTRAZZA | NECITUMUMAB | 125547 | 10227328 | 2/4/2035 |
| PORTRAZZA | NECITUMUMAB | 125547 | 10259859 | 8/7/2035 |
| PORTRAZZA | NECITUMUMAB | 125547 | 10369114 | 3/14/2033 |
| PORTRAZZA | NECITUMUMAB | 125547 | 10548985 | 1/10/2034 |
| PORTRAZZA | NECITUMUMAB | 125547 | 10562974 | 3/13/2033 |
| PORTRAZZA | NECITUMUMAB | 125547 | 10583111 | 12/13/2037 |
| PORTRAZZA | NECITUMUMAB | 125547 | 10590182 | 2/24/2035 |
| PORTRAZZA | NECITUMUMAB | 125547 | 10611754 | 2/4/2035 |
| PORTRAZZA | NECITUMUMAB | 125547 | 10611844 | 12/21/2032 |
| PORTRAZZA | NECITUMUMAB | 125547 | 10632123 | 3/10/2036 |
| PRALUENT | ALIROCUMAB | 125559 | 10058630 | 10/22/2032 |
| PRALUENT | ALIROCUMAB | 125559 | 10154813 | 7/3/2037 |
| PRALUENT | ALIROCUMAB | 125559 | 10253102 | 10/12/2035 |
| PRALUENT | ALIROCUMAB | 125559 | 10337070 | 1/12/2037 |
| PRALUENT | ALIROCUMAB | 125559 | 10369114 | 3/14/2033 |
| PRALUENT | ALIROCUMAB | 125559 | 10472424 | 9/23/2034 |
| PRALUENT | ALIROCUMAB | 125559 | 10590182 | 2/24/2035 |
| PRALUENT | ALIROCUMAB | 125559 | 10611849 | 12/17/2033 |
| PRALUENT | ALIROCUMAB | 125559 | 10618971 | 12/17/2033 |
| PRAXBIND | IDARUCIZUMAB | 761025 | 10501773 | 7/31/2034 |
| PRAXBIND | IDARUCIZUMAB | 761025 | 10590182 | 2/24/2035 |
| PREVYMIS | LETERMOVIR | 209940 | 10603384 | 2/28/2033 |
| PROLIA | DENOSUMAB | 125320 | 10006091 | 10/17/2038 |
| PROLIA | DENOSUMAB | 125320 | 10016338 | 3/11/2033 |
| PROLIA | DENOSUMAB | 125320 | 10047398 | 10/6/2030 |
| PROLIA | DENOSUMAB | 125320 | 10058630 | 10/22/2032 |
| PROLIA | DENOSUMAB | 125320 | 10065934 | 7/17/2034 |
| PROLIA | DENOSUMAB | 125320 | 10092542 | 3/20/2022 |
| PROLIA | DENOSUMAB | 125320 | 10111968 | 2/15/2032 |
| PROLIA | DENOSUMAB | 125320 | 10114022 | 11/19/2038 |
| PROLIA | DENOSUMAB | 125320 | 10119171 | 10/9/2033 |
| PROLIA | DENOSUMAB | 125320 | 10125398 | 6/13/2034 |
| PROLIA | DENOSUMAB | 125320 | 10143747 | 9/22/2030 |
| PROLIA | DENOSUMAB | 125320 | 10154856 | 2/6/2033 |
| PROLIA | DENOSUMAB | 125320 | 10155069 | 12/20/2038 |
| PROLIA | DENOSUMAB | 125320 | 10196693 | 12/12/2034 |
| PROLIA | DENOSUMAB | 125320 | 10220072 | 6/30/2036 |
| PROLIA | DENOSUMAB | 125320 | 10246513 | 8/7/2033 |
| PROLIA | DENOSUMAB | 125320 | 10259859 | 8/7/2035 |
| PROLIA | DENOSUMAB | 125320 | 10261083 | 1/4/2033 |
| PROLIA | DENOSUMAB | 125320 | 10265291 | 7/15/2035 |
| PROLIA | DENOSUMAB | 125320 | 10267754 | 4/12/2033 |
| PROLIA | DENOSUMAB | 125320 | 10273252 | 4/28/2036 |
| PROLIA | DENOSUMAB | 125320 | 10287294 | 7/8/2035 |
| PROLIA | DENOSUMAB | 125320 | 10316282 | 3/19/2033 |
| PROLIA | DENOSUMAB | 125320 | 10328157 | 7/15/2035 |
| PROLIA | DENOSUMAB | 125320 | 10342790 | 2/20/2037 |
| PROLIA | DENOSUMAB | 125320 | 10383876 | 4/5/2031 |
| PROLIA | DENOSUMAB | 125320 | 10407673 | 8/24/2036 |
| PROLIA | DENOSUMAB | 125320 | 10434082 | 7/26/2032 |
| PROLIA | DENOSUMAB | 125320 | 10493113 | 11/14/2037 |
| PROLIA | DENOSUMAB | 125320 | 10514347 | 4/12/2033 |
| PROLIA | DENOSUMAB | 125320 | 10561632 | 7/26/2032 |
| PROLIA | DENOSUMAB | 125320 | 10576160 | 7/27/2029 |
| PROLIA | DENOSUMAB | 125320 | 10577154 | 5/13/2029 |
| PROLIA | DENOSUMAB | 125320 | 10584347 | 3/16/2036 |
| PROLIA | DENOSUMAB | 125320 | 10590182 | 2/24/2035 |
| PROLIA | DENOSUMAB | 125320 | 10596112 | 10/6/2034 |
| PROLIA | DENOSUMAB | 125320 | 10610602 | 9/20/2037 |
| PROLIA | DENOSUMAB | 125320 | 10618935 | 11/3/2035 |
| PROLIA | DENOSUMAB | 125320 | 10653667 | 9/25/2033 |
| PROLIA | DENOSUMAB | 125320 | 10669337 | 7/25/2034 |
| PROVENGE | sipuleucel-T | 125197 | 10071953 | 4/13/2031 |
| PROVENGE | sipuleucel-T | 125197 | 10196695 | 12/2/2033 |
| PROVENGE | sipuleucel-T | 125197 | 10196696 | 12/2/2033 |
| PROVENGE | sipuleucel-T | 125197 | 10196697 | 12/12/2033 |
| PROVENGE | sipuleucel-T | 125197 | 10232051 | 7/15/2035 |
| PROVENGE | sipuleucel-T | 125197 | 10261091 | 9/5/2033 |
| PROVENGE | sipuleucel-T | 125197 | 10265291 | 7/15/2035 |
| PROVENGE | sipuleucel-T | 125197 | 10287294 | 7/8/2035 |
| PROVENGE | sipuleucel-T | 125197 | 10287634 | 12/2/2033 |
| PROVENGE | sipuleucel-T | 125197 | 10301683 | 12/2/2033 |
| PROVENGE | sipuleucel-T | 125197 | 10322104 | 7/15/2035 |
| PROVENGE | sipuleucel-T | 125197 | 10328157 | 7/15/2035 |
| PROVENGE | sipuleucel-T | 125197 | 10383876 | 4/5/2031 |
| PROVENGE | sipuleucel-T | 125197 | 10435472 | 9/17/2034 |
| PROVENGE | sipuleucel-T | 125197 | 10501436 | 1/1/2034 |
| PROVENGE | sipuleucel-T | 125197 | 10683314 | 2/28/2034 |
| PROVENGE | sipuleucel-T | 125197 | 10696699 | 2/28/2034 |
| RAPIVAB | PERAMIVIR | 206426 | 10391075 | 2/12/2027 |
| REPATHA | EVOLOCUMAB | 125522 | 10058630 | 10/22/2032 |
| REPATHA | EVOLOCUMAB | 125522 | 10154813 | 7/3/2037 |
| REPATHA | EVOLOCUMAB | 125522 | 10253102 | 10/12/2035 |
| REPATHA | EVOLOCUMAB | 125522 | 10337070 | 1/12/2037 |
| REPATHA | EVOLOCUMAB | 125522 | 10357476 | 10/30/2038 |
| REPATHA | EVOLOCUMAB | 125522 | 10369114 | 3/14/2033 |
| REPATHA | EVOLOCUMAB | 125522 | 10472424 | 9/23/2034 |
| REPATHA | EVOLOCUMAB | 125522 | 10590182 | 2/24/2035 |
| REXULTI | BREXPIPRAZOLE | 205422 | 10307419 | 10/12/2032 |
| RHOPRESSA | NETARSUDIL DIMESYLATE | 208254 | 10174017 | 1/27/2030 |
| RHOPRESSA | NETARSUDIL MESYLATE | 208254 | 10532993 | 7/11/2026 |
| RHOPRESSA | NETARSUDIL MESYLATE | 208254 | 10588901 | 3/14/2034 |
| RINVOQ | UPADACITINIB | 211675 | 10519164 | 10/17/2036 |
| RINVOQ | UPADACITINIB | 211675 | 10597400 | 10/17/2036 |
| ROZLYTREK | ENTRECTINIB | 212725 | 10231965 | 2/17/2035 |
| ROZLYTREK | ENTRECTINIB | 212725 | 10398693 | 7/18/2038 |
| ROZLYTREK | ENTRECTINIB | 212725 | 10561651 | 2/19/2035 |
| RUBRACA | RUCAPARIB CAMSYLATE | 209115 | 10130636 | 8/17/2035 |
| RUBRACA | RUCAPARIB CAMSYLATE | 209115 | 10278974 | 2/10/2031 |
| SCENESSE | AFAMELANOTIDE | 210797 | 10076555 | 2/11/2025 |
| SILIQ | BRODALUMAB | 761032 | 10039810 | 5/15/2034 |
| SILIQ | BRODALUMAB | 761032 | 10111968 | 2/15/2032 |
| SILIQ | BRODALUMAB | 761032 | 10369114 | 3/14/2033 |
| SILIQ | BRODALUMAB | 761032 | 10590182 | 2/24/2035 |
| SPINRAZA | NUSINERSEN SODIUM | 209531 | 10266822 | 12/5/2025 |
| SPINRAZA | NUSINERSEN SODIUM | 209531 | 10436802 | 9/11/2035 |
| STRENSIQ | ASFOTASE ALFA | 125513 | 10603361 | 1/28/2035 |
| SUNOSI | SOLRIAMFETOL | 211230 | 10195151 | 9/5/2037 |
| SUNOSI | SOLRIAMFETOL | 211230 | 10351517 | 6/7/2026 |
| SUNOSI | SOLRIAMFETOL HYDROCHLORIDE | 211230 | 10512609 | 9/5/2037 |
| SURFAXIN | LUCINACTANT | 21746 | 5407914 | 11/17/2014 |
| SYLVANT | SILTUXIMAB | 125496 | 10047163 | 2/8/2033 |
| SYLVANT | SILTUXIMAB | 125496 | 10111968 | 2/15/2032 |
| SYLVANT | SILTUXIMAB | 125496 | 10265291 | 7/15/2035 |
| SYLVANT | SILTUXIMAB | 125496 | 10328157 | 7/15/2035 |
| SYLVANT | SILTUXIMAB | 125496 | 10590182 | 2/24/2035 |
| TAGRISSO | OSIMERTINIB MESYLATE | 208065 | 10183020 | 1/2/2035 |
| TALTZ | IXEKIZUMAB | 125521 | 10039810 | 5/15/2034 |
| TALTZ | IXEKIZUMAB | 125521 | 10111968 | 2/15/2032 |
| TALTZ | IXEKIZUMAB | 125521 | 10227403 | 6/25/2032 |
| TALTZ | IXEKIZUMAB | 125521 | 10273252 | 4/28/2036 |
| TALTZ | IXEKIZUMAB | 125521 | 10369114 | 3/14/2033 |
| TALTZ | IXEKIZUMAB | 125521 | 10519228 | 5/8/2038 |
| TALTZ | IXEKIZUMAB | 125521 | 10590182 | 2/24/2035 |
| TALZENNA | TALAZOPARIB TOSYLATE | 211651 | 10189837 | 10/20/2031 |
| TANZEUM | ALBIGLUTIDE | 125431 | 10004747 | 5/5/2030 |
| TANZEUM | ALBIGLUTIDE | 125431 | 10029080 | 12/23/2030 |
| TANZEUM | ALBIGLUTIDE | 125431 | 10071992 | 10/17/2038 |
| TANZEUM | ALBIGLUTIDE | 125431 | 10179228 | 12/24/2029 |
| TANZEUM | ALBIGLUTIDE | 125431 | 10252039 | 12/24/2029 |
| TANZEUM | ALBIGLUTIDE | 125431 | 10253102 | 10/12/2035 |
| TANZEUM | ALBIGLUTIDE | 125431 | 10258639 | 5/6/2034 |
| TANZEUM | ALBIGLUTIDE | 125431 | 10369114 | 3/14/2033 |
| TANZEUM | ALBIGLUTIDE | 125431 | 10383918 | 8/29/2039 |
| TANZEUM | ALBIGLUTIDE | 125431 | 10392357 | 6/30/2035 |
| TANZEUM | ALBIGLUTIDE | 125431 | 10485851 | 6/7/2036 |
| TANZEUM | ALBIGLUTIDE | 125431 | 10487129 | 1/23/2029 |
| TANZEUM | ALBIGLUTIDE | 125431 | 10493253 | 12/24/2029 |
| TANZEUM | ALBIGLUTIDE | 125431 | 10548850 | 12/23/2030 |
| TANZEUM | ALBIGLUTIDE | 125431 | 10548952 | 6/7/2036 |
| TANZEUM | ALBIGLUTIDE | 125431 | 10562909 | 2/25/2040 |
| TANZEUM | ALBIGLUTIDE | 125431 | 10596359 | 12/24/2029 |
| TANZEUM | ALBIGLUTIDE | 125431 | 10603475 | 12/24/2029 |
| TANZEUM | ALBIGLUTIDE | 125431 | 10639308 | 3/15/2033 |
| TANZEUM | ALBIGLUTIDE | 125431 | 10660939 | 4/14/2035 |
| TECENTRIQ | ATEZOLIZUMAB | 761034 | 10001483 | 6/26/2035 |
| TECENTRIQ | ATEZOLIZUMAB | 761041 | 10001483 | 6/26/2035 |
| TECENTRIQ | ATEZOLIZUMAB | 761034 | 10143723 | 12/23/2035 |
| TECENTRIQ | ATEZOLIZUMAB | 761041 | 10143723 | 12/23/2035 |
| TECENTRIQ | ATEZOLIZUMAB | 761034 | 10172808 | 5/18/2036 |
| TECENTRIQ | ATEZOLIZUMAB | 761041 | 10172808 | 5/18/2036 |
| TECENTRIQ | ATEZOLIZUMAB | 761034 | 10195175 | 6/25/2035 |
| TECENTRIQ | ATEZOLIZUMAB | 761041 | 10195175 | 6/25/2035 |
| TECENTRIQ | ATEZOLIZUMAB | 761034 | 10195188 | 6/13/2036 |
| TECENTRIQ | ATEZOLIZUMAB | 761041 | 10195188 | 6/13/2036 |
| TECENTRIQ | ATEZOLIZUMAB | 761034 | 10195273 | 6/5/2036 |
| TECENTRIQ | ATEZOLIZUMAB | 761041 | 10195273 | 6/5/2036 |
| TECENTRIQ | ATEZOLIZUMAB | 761034 | 10206910 | 11/7/2034 |
| TECENTRIQ | ATEZOLIZUMAB | 761041 | 10206910 | 11/7/2034 |
| TECENTRIQ | ATEZOLIZUMAB | 761034 | 10214585 | 3/12/2034 |
| TECENTRIQ | ATEZOLIZUMAB | 761041 | 10214585 | 3/12/2034 |
| TECENTRIQ | ATEZOLIZUMAB | 761034 | 10220072 | 6/30/2036 |
| TECENTRIQ | ATEZOLIZUMAB | 761041 | 10220072 | 6/30/2036 |
| TECENTRIQ | ATEZOLIZUMAB | 761034 | 10259859 | 8/7/2035 |
| TECENTRIQ | ATEZOLIZUMAB | 761041 | 10259859 | 8/7/2035 |
| TECENTRIQ | ATEZOLIZUMAB | 761034 | 10265291 | 7/15/2035 |
| TECENTRIQ | ATEZOLIZUMAB | 761041 | 10265291 | 7/15/2035 |
| TECENTRIQ | ATEZOLIZUMAB | 761034 | 10273252 | 4/28/2036 |
| TECENTRIQ | ATEZOLIZUMAB | 761041 | 10273252 | 4/28/2036 |
| TECENTRIQ | ATEZOLIZUMAB | 761034 | 10292951 | 9/1/2035 |
| TECENTRIQ | ATEZOLIZUMAB | 761041 | 10292951 | 9/1/2035 |
| TECENTRIQ | ATEZOLIZUMAB | 761034 | 10300138 | 6/5/2036 |
| TECENTRIQ | ATEZOLIZUMAB | 761041 | 10300138 | 6/5/2036 |
| TECENTRIQ | ATEZOLIZUMAB | 761034 | 10300139 | 6/5/2036 |
| TECENTRIQ | ATEZOLIZUMAB | 761041 | 10300139 | 6/5/2036 |
| TECENTRIQ | ATEZOLIZUMAB | 761034 | 10328157 | 7/15/2035 |
| TECENTRIQ | ATEZOLIZUMAB | 761041 | 10328157 | 7/15/2035 |
| TECENTRIQ | ATEZOLIZUMAB | 761034 | 10335486 | 5/18/2036 |
| TECENTRIQ | ATEZOLIZUMAB | 761041 | 10335486 | 5/18/2036 |
| TECENTRIQ | ATEZOLIZUMAB | 761034 | 10350213 | 7/30/2039 |
| TECENTRIQ | ATEZOLIZUMAB | 761041 | 10350213 | 7/30/2039 |
| TECENTRIQ | ATEZOLIZUMAB | 761034 | 10363308 | 6/5/2036 |
| TECENTRIQ | ATEZOLIZUMAB | 761041 | 10363308 | 6/5/2036 |
| TECENTRIQ | ATEZOLIZUMAB | 761034 | 10370346 | 8/6/2033 |
| TECENTRIQ | ATEZOLIZUMAB | 761041 | 10370346 | 8/6/2033 |
| TECENTRIQ | ATEZOLIZUMAB | 761034 | 10385131 | 5/11/2036 |
| TECENTRIQ | ATEZOLIZUMAB | 761041 | 10385131 | 5/11/2036 |
| TECENTRIQ | ATEZOLIZUMAB | 761034 | 10398685 | 6/13/2036 |
| TECENTRIQ | ATEZOLIZUMAB | 761041 | 10398685 | 6/13/2036 |
| TECENTRIQ | ATEZOLIZUMAB | 761034 | 10406278 | 2/11/2036 |
| TECENTRIQ | ATEZOLIZUMAB | 761041 | 10406278 | 2/11/2036 |
| TECENTRIQ | ATEZOLIZUMAB | 761034 | 10492723 | 2/27/2037 |
| TECENTRIQ | ATEZOLIZUMAB | 761041 | 10492723 | 2/27/2037 |
| TECENTRIQ | ATEZOLIZUMAB | 761034 | 10507244 | 10/1/2035 |
| TECENTRIQ | ATEZOLIZUMAB | 761041 | 10507244 | 10/1/2035 |
| TECENTRIQ | ATEZOLIZUMAB | 761034 | 10512630 | 9/30/2035 |
| TECENTRIQ | ATEZOLIZUMAB | 761041 | 10512630 | 9/30/2035 |
| TECENTRIQ | ATEZOLIZUMAB | 761034 | 10544223 | 4/20/2037 |
| TECENTRIQ | ATEZOLIZUMAB | 761041 | 10544223 | 4/20/2037 |
| TECENTRIQ | ATEZOLIZUMAB | 761034 | 10568870 | 4/7/2036 |
| TECENTRIQ | ATEZOLIZUMAB | 761041 | 10568870 | 4/7/2036 |
| TECENTRIQ | ATEZOLIZUMAB | 761034 | 10576111 | 7/5/2037 |
| TECENTRIQ | ATEZOLIZUMAB | 761041 | 10576111 | 7/5/2037 |
| TECENTRIQ | ATEZOLIZUMAB | 761034 | 10583131 | 6/13/2036 |
| TECENTRIQ | ATEZOLIZUMAB | 761041 | 10583131 | 6/13/2036 |
| TECENTRIQ | ATEZOLIZUMAB | 761034 | 10584169 | 1/24/2037 |
| TECENTRIQ | ATEZOLIZUMAB | 761041 | 10584169 | 1/24/2037 |
| TECENTRIQ | ATEZOLIZUMAB | 761034 | 10590182 | 2/24/2035 |
| TECENTRIQ | ATEZOLIZUMAB | 761041 | 10590182 | 2/24/2035 |
| TECENTRIQ | ATEZOLIZUMAB | 761034 | 10596257 | 1/8/2036 |
| TECENTRIQ | ATEZOLIZUMAB | 761041 | 10596257 | 1/8/2036 |
| TECENTRIQ | ATEZOLIZUMAB | 761034 | 10603379 | 6/5/2036 |
| TECENTRIQ | ATEZOLIZUMAB | 761041 | 10603379 | 6/5/2036 |
| TECENTRIQ | ATEZOLIZUMAB | 761034 | 10617758 | 10/9/2035 |
| TECENTRIQ | ATEZOLIZUMAB | 761041 | 10617758 | 10/9/2035 |
| TECENTRIQ | ATEZOLIZUMAB | 761041 | 10660891 | 6/8/2038 |
| TECENTRIQ | ATEZOLIZUMAB | 761041 | 10660909 | 6/4/2040 |
| TECENTRIQ | ATEZOLIZUMAB | 761041 | 10662252 | 8/14/2032 |
| TECENTRIQ | ATEZOLIZUMAB | 761041 | 10669337 | 7/25/2034 |
| TECENTRIQ | ATEZOLIZUMAB | 761041 | 10669338 | 6/17/2036 |
| TIBSOVO | IVOSIDENIB | 211192 | 10449184 | 3/13/2035 |
| TIBSOVO | IVOSIDENIB | 211192 | 10610125 | 6/21/2030 |
| TRADJENTA | LINAGLIPTIN | 201280 | 10034877 | 8/5/2029 |
| TREMFYA | GUSELKUMAB | 761061 | 10273252 | 4/28/2036 |
| TREMFYA | GUSELKUMAB | 761061 | 10590182 | 2/24/2035 |
| TRESIBA | INSULIN DEGLUDEC | 203314 | 10137172 | 4/30/2033 |
| TRESIBA | INSULIN DEGLUDEC | 203314 | 10220155 | 7/17/2026 |
| TRESIBA | INSULIN DEGLUDEC | 203314 | 10335464 | 4/1/2040 |
| TRESIBA | INSULIN DEGLUDEC | 203314 | 10357616 | 1/20/2026 |
| TRESIBA | INSULIN DEGLUDEC | 203314 | 10369114 | 3/14/2033 |
| TRESIBA | INSULIN DEGLUDEC | 203314 | 10376652 | 1/20/2026 |
| TRESIBA | INSULIN DEGLUDEC | 203314 | 10493125 | 12/9/2035 |
| TRESIBA | INSULIN DEGLUDEC | 203314 | 10610595 | 1/9/2034 |
| TRESIBA | INSULIN DEGLUDEC | 203314 | 10639308 | 3/15/2033 |
| TRULANCE | PLECANATIDE | 208745 | 10011637 | 6/5/2034 |
| TRULICITY | DULAGLUTIDE | 125469 | 10004747 | 5/5/2030 |
| TRULICITY | DULAGLUTIDE | 125469 | 10071992 | 10/17/2038 |
| TRULICITY | DULAGLUTIDE | 125469 | 10253102 | 10/12/2035 |
| TRULICITY | DULAGLUTIDE | 125469 | 10369114 | 3/14/2033 |
| TRULICITY | DULAGLUTIDE | 125469 | 10383918 | 8/29/2039 |
| TRULICITY | DULAGLUTIDE | 125469 | 10392357 | 6/30/2035 |
| TRULICITY | DULAGLUTIDE | 125469 | 10485851 | 6/7/2036 |
| TRULICITY | DULAGLUTIDE | 125469 | 10548952 | 6/7/2036 |
| TRULICITY | DULAGLUTIDE | 125469 | 10639308 | 3/15/2033 |
| TRULICITY | DULAGLUTIDE | 125469 | 10660939 | 4/14/2035 |
| TUDORZA PRESSAIR | ACLIDINIUM BROMIDE | 202450 | 10034867 | 7/7/2020 |
| TUDORZA PRESSAIR | ACLIDINIUM BROMIDE | 202450 | 10085974 | 3/13/2029 |
| TUDORZA PRESSAIR | ACLIDINIUM BROMIDE | 202450 | 10588895 | 7/7/2020 |
| TURALIO | PEXIDARTINIB HYDROCHLORIDE | 211810 | 10189833 | 5/5/2036 |
| TURALIO | PEXIDARTINIB HYDROCHLORIDE | 211810 | 10435404 | 7/24/2038 |
| UBRELVY | UBROGEPANT | 211765 | 10117836 | 1/30/2035 |
| UNITUXIN | DINUTUXIMAB | 125516 | 10072065 | 8/24/2035 |
| UNITUXIN | DINUTUXIMAB | 125516 | 10220072 | 6/30/2036 |
| UNITUXIN | DINUTUXIMAB | 125516 | 10265291 | 7/15/2035 |
| UNITUXIN | DINUTUXIMAB | 125516 | 10328157 | 7/15/2035 |
| UNITUXIN | DINUTUXIMAB | 125516 | 10590182 | 2/24/2035 |
| UNITUXIN | DINUTUXIMAB | 125516 | 10596112 | 10/6/2034 |
| VASCEPA | ICOSAPENT ETHYL | 202057 | 10010517 | 4/29/2030 |
| VASCEPA | ICOSAPENT ETHYL | 202057 | 10265287 | 4/29/2030 |
| VASCEPA | ICOSAPENT ETHYL | 202057 | 10278935 | 6/28/2033 |
| VASCEPA | ICOSAPENT ETHYL | 202057 | 10278936 | 6/28/2033 |
| VASCEPA | ICOSAPENT ETHYL | 202057 | 10278937 | 6/28/2033 |
| VASCEPA | ICOSAPENT ETHYL | 202057 | 10383840 | 6/28/2033 |
| VASCEPA | ICOSAPENT ETHYL | 202057 | 10555924 | 6/28/2033 |
| VASCEPA | ICOSAPENT ETHYL | 202057 | 10555925 | 6/28/2033 |
| VASCEPA | ICOSAPENT ETHYL | 202057 | 10568861 | 6/28/2033 |
| VASCEPA | ICOSAPENT ETHYL | 202057 | 10576054 | 6/28/2033 |
| VELTASSA | PATIROMER SORBITEX CALCIUM | 205739 | 10485821 | 3/30/2024 |
| VIBERZI | ELUXADOLINE | 206940 | 10188632 | 3/14/2033 |
| VIBERZI | ELUXADOLINE | 206940 | 10213415 | 3/14/2025 |
| VITRAKVI | LAROTRECTINIB | 210861 | 10005783 | 10/21/2029 |
| VITRAKVI | LAROTRECTINIB | 211710 | 10005783 | 10/21/2029 |
| VITRAKVI | LAROTRECTINIB | 211710 | 10045991 | 4/4/2037 |
| VITRAKVI | LAROTRECTINIB | 210861 | 10047097 | 10/21/2029 |
| VITRAKVI | LAROTRECTINIB | 211710 | 10047097 | 10/21/2029 |
| VITRAKVI | LAROTRECTINIB | 211710 | 10137127 | 4/4/2037 |
| VITRAKVI | LAROTRECTINIB SULFATE | 210861 | 10172861 | 11/16/2035 |
| VITRAKVI | LAROTRECTINIB SULFATE | 211710 | 10172861 | 11/16/2035 |
| VITRAKVI | LAROTRECTINIB SULFATE | 210861 | 10285993 | 11/16/2035 |
| VONVENDI | VON WILLEBRAND FACTOR (RECOMBINANT) | 125577 | 10232015 | 5/8/2032 |
| VPRIV | VELAGLUCERASE ALFA | 22575 | 10406278 | 2/11/2036 |
| VPRIV | VELAGLUCERASE ALFA | 22575 | 10668053 | 5/3/2033 |
| VPRIV | VELAGLUCERASE ALFA | 22575 | 10675328 | 2/14/2037 |
| VYONDYS 53 | GOLODIRSEN | 211970 | 10227590 | 6/28/2025 |
| VYONDYS 53 | GOLODIRSEN | 211970 | 10266827 | 6/28/2025 |
| VYONDYS 53 | GOLODIRSEN | 211970 | 10421966 | 6/28/2025 |
| VYONDYS 53 | GOLODIRSEN | 211970 | 10533174 | 5/4/2021 |
| Xeomin | incobotulinumtoxinA | 125360 | 10016338 | 3/11/2033 |
| Xeomin | incobotulinumtoxinA | 125360 | 10406213 | 3/30/2030 |
| Xeomin | incobotulinumtoxinA | 125360 | 10577154 | 5/13/2029 |
| XIAFLEX | COLLAGENASE CLOSTRIDIUM HISTOLYTICUM | 125338 | 10369110 | 3/15/2033 |
| XIIDRA | LIFITEGRAST | 208073 | 10124000 | 11/5/2024 |
| XOFLUZA | BALOXAVIR MARBOXIL | 210854 | 10392406 | 4/27/2036 |
| XOFLUZA | BALOXAVIR MARBOXIL | 210854 | 10633397 | 4/27/2036 |
| XPOVIO | SELINEXOR | 212306 | 10519139 | 8/14/2035 |
| XPOVIO | SELINEXOR | 212306 | 10544108 | 7/26/2032 |
| YERVOY | IPILIMUMAB | 125377 | 10016365 | 10/10/2038 |
| YERVOY | IPILIMUMAB | 125377 | 10016421 | 4/5/2034 |
| YERVOY | IPILIMUMAB | 125377 | 10022451 | 10/10/2038 |
| YERVOY | IPILIMUMAB | 125377 | 10034938 | 8/30/2032 |
| YERVOY | IPILIMUMAB | 125377 | 10047163 | 2/8/2033 |
| YERVOY | IPILIMUMAB | 125377 | 10065934 | 7/17/2034 |
| YERVOY | IPILIMUMAB | 125377 | 10071962 | 5/30/2034 |
| YERVOY | IPILIMUMAB | 125377 | 10072082 | 5/15/2032 |
| YERVOY | IPILIMUMAB | 125377 | 10081681 | 9/20/2033 |
| YERVOY | IPILIMUMAB | 125377 | 10082510 | 1/30/2035 |
| YERVOY | IPILIMUMAB | 125377 | 10100034 | 5/4/2035 |
| YERVOY | IPILIMUMAB | 125377 | 10105389 | 4/1/2036 |
| YERVOY | IPILIMUMAB | 125377 | 10106546 | 11/5/2034 |
| YERVOY | IPILIMUMAB | 125377 | 10111900 | 11/6/2034 |
| YERVOY | IPILIMUMAB | 125377 | 10111968 | 2/15/2032 |
| YERVOY | IPILIMUMAB | 125377 | 10130582 | 2/9/2035 |
| YERVOY | IPILIMUMAB | 125377 | 10130718 | 1/7/2033 |
| YERVOY | IPILIMUMAB | 125377 | 10143723 | 12/23/2035 |
| YERVOY | IPILIMUMAB | 125377 | 10159745 | 4/10/2035 |
| YERVOY | IPILIMUMAB | 125377 | 10166290 | 1/22/2039 |
| YERVOY | IPILIMUMAB | 125377 | 10167254 | 4/3/2035 |
| YERVOY | IPILIMUMAB | 125377 | 10172808 | 5/18/2036 |
| YERVOY | IPILIMUMAB | 125377 | 10174113 | 4/28/2035 |
| YERVOY | IPILIMUMAB | 125377 | 10179770 | 1/16/2039 |
| YERVOY | IPILIMUMAB | 125377 | 10189797 | 12/30/2035 |
| YERVOY | IPILIMUMAB | 125377 | 10195175 | 6/25/2035 |
| YERVOY | IPILIMUMAB | 125377 | 10206893 | 11/5/2034 |
| YERVOY | IPILIMUMAB | 125377 | 10220072 | 6/30/2036 |
| YERVOY | IPILIMUMAB | 125377 | 10221140 | 8/8/2034 |
| YERVOY | IPILIMUMAB | 125377 | 10227295 | 7/30/2033 |
| YERVOY | IPILIMUMAB | 125377 | 10232051 | 7/15/2035 |
| YERVOY | IPILIMUMAB | 125377 | 10238631 | 4/25/2039 |
| YERVOY | IPILIMUMAB | 125377 | 10258619 | 10/5/2035 |
| YERVOY | IPILIMUMAB | 125377 | 10259859 | 8/7/2035 |
| YERVOY | IPILIMUMAB | 125377 | 10261083 | 1/4/2033 |
| YERVOY | IPILIMUMAB | 125377 | 10265291 | 7/15/2035 |
| YERVOY | IPILIMUMAB | 125377 | 10266591 | 7/2/2032 |
| YERVOY | IPILIMUMAB | 125377 | 10266605 | 4/27/2036 |
| YERVOY | IPILIMUMAB | 125377 | 10273252 | 4/28/2036 |
| YERVOY | IPILIMUMAB | 125377 | 10278954 | 2/1/2036 |
| YERVOY | IPILIMUMAB | 125377 | 10278984 | 5/25/2039 |
| YERVOY | IPILIMUMAB | 125377 | 10287294 | 7/8/2035 |
| YERVOY | IPILIMUMAB | 125377 | 10287362 | 11/10/2034 |
| YERVOY | IPILIMUMAB | 125377 | 10292951 | 9/1/2035 |
| YERVOY | IPILIMUMAB | 125377 | 10301280 | 12/21/2032 |
| YERVOY | IPILIMUMAB | 125377 | 10322104 | 7/15/2035 |
| YERVOY | IPILIMUMAB | 125377 | 10323004 | 5/4/2036 |
| YERVOY | IPILIMUMAB | 125377 | 10328157 | 7/15/2035 |
| YERVOY | IPILIMUMAB | 125377 | 10328158 | 1/10/2034 |
| YERVOY | IPILIMUMAB | 125377 | 10335388 | 4/17/2035 |
| YERVOY | IPILIMUMAB | 125377 | 10335486 | 5/18/2036 |
| YERVOY | IPILIMUMAB | 125377 | 10342790 | 2/20/2037 |
| YERVOY | IPILIMUMAB | 125377 | 10342866 | 4/29/2035 |
| YERVOY | IPILIMUMAB | 125377 | 10351627 | 7/27/2039 |
| YERVOY | IPILIMUMAB | 125377 | 10358496 | 3/1/2036 |
| YERVOY | IPILIMUMAB | 125377 | 10363308 | 6/5/2036 |
| YERVOY | IPILIMUMAB | 125377 | 10369114 | 3/14/2033 |
| YERVOY | IPILIMUMAB | 125377 | 10377824 | 7/2/2032 |
| YERVOY | IPILIMUMAB | 125377 | 10383876 | 4/5/2031 |
| YERVOY | IPILIMUMAB | 125377 | 10406278 | 2/11/2036 |
| YERVOY | IPILIMUMAB | 125377 | 10428067 | 6/7/2037 |
| YERVOY | IPILIMUMAB | 125377 | 10428143 | 9/28/2034 |
| YERVOY | IPILIMUMAB | 125377 | 10434082 | 7/26/2032 |
| YERVOY | IPILIMUMAB | 125377 | 10463686 | 9/15/2036 |
| YERVOY | IPILIMUMAB | 125377 | 10463690 | 4/1/2036 |
| YERVOY | IPILIMUMAB | 125377 | 10471023 | 3/12/2035 |
| YERVOY | IPILIMUMAB | 125377 | 10485796 | 6/29/2035 |
| YERVOY | IPILIMUMAB | 125377 | 10501436 | 1/1/2034 |
| YERVOY | IPILIMUMAB | 125377 | 10512662 | 2/25/2036 |
| YERVOY | IPILIMUMAB | 125377 | 10512689 | 4/17/2035 |
| YERVOY | IPILIMUMAB | 125377 | 10519187 | 2/13/2038 |
| YERVOY | IPILIMUMAB | 125377 | 10532096 | 10/18/2033 |
| YERVOY | IPILIMUMAB | 125377 | 10533014 | 11/5/2034 |
| YERVOY | IPILIMUMAB | 125377 | 10543283 | 3/13/2033 |
| YERVOY | IPILIMUMAB | 125377 | 10544099 | 5/4/2036 |
| YERVOY | IPILIMUMAB | 125377 | 10544125 | 5/4/2035 |
| YERVOY | IPILIMUMAB | 125377 | 10544223 | 4/20/2037 |
| YERVOY | IPILIMUMAB | 125377 | 10561632 | 7/26/2032 |
| YERVOY | IPILIMUMAB | 125377 | 10576111 | 7/5/2037 |
| YERVOY | IPILIMUMAB | 125377 | 10584169 | 1/24/2037 |
| YERVOY | IPILIMUMAB | 125377 | 10590182 | 2/24/2035 |
| YERVOY | IPILIMUMAB | 125377 | 10596112 | 10/6/2034 |
| YERVOY | IPILIMUMAB | 125377 | 10597411 | 4/1/2040 |
| YERVOY | IPILIMUMAB | 125377 | 10603379 | 6/5/2036 |
| YERVOY | IPILIMUMAB | 125377 | 10604542 | 1/11/2036 |
| YERVOY | IPILIMUMAB | 125377 | 10610602 | 9/20/2037 |
| YERVOY | IPILIMUMAB | 125377 | 10610605 | 2/15/2032 |
| YERVOY | IPILIMUMAB | 125377 | 10624965 | 12/19/2034 |
| YERVOY | IPILIMUMAB | 125377 | 10632143 | 5/2/2040 |
| YERVOY | IPILIMUMAB | 125377 | 10632193 | 12/24/2034 |
| YERVOY | IPILIMUMAB | 125377 | 10633342 | 5/4/2036 |
| YERVOY | IPILIMUMAB | 125377 | 10633374 | 8/6/2035 |
| YERVOY | IPILIMUMAB | 125377 | 10653774 | 5/18/2033 |
| YERVOY | IPILIMUMAB | 125377 | 10653793 | 12/13/2032 |
| YERVOY | IPILIMUMAB | 125377 | 10654811 | 1/23/2035 |
| YERVOY | IPILIMUMAB | 125377 | 10660891 | 6/8/2038 |
| YERVOY | IPILIMUMAB | 125377 | 10660909 | 6/4/2040 |
| YERVOY | IPILIMUMAB | 125377 | 10669337 | 7/25/2034 |
| YERVOY | IPILIMUMAB | 125377 | 10669338 | 6/17/2036 |
| YUPELRI | REVEFENACIN | 210598 | 10106503 | 3/10/2025 |
| YUPELRI | REVEFENACIN | 210598 | 10343995 | 3/10/2025 |
| YUPELRI | REVEFENACIN | 210598 | 10550081 | 7/14/2030 |
| ZALTRAP | ZIV-AFLIBERCEPT | 125418 | 10172817 | 10/31/2032 |
| ZALTRAP | ZIV-AFLIBERCEPT | 125418 | 10265291 | 7/15/2035 |
| ZALTRAP | ZIV-AFLIBERCEPT | 125418 | 10287294 | 7/8/2035 |
| ZALTRAP | ZIV-AFLIBERCEPT | 125418 | 10328157 | 7/15/2035 |
| ZALTRAP | ZIV-AFLIBERCEPT | 125418 | 10501523 | 7/18/2034 |
| ZALTRAP | ZIV-AFLIBERCEPT | 125418 | 10525104 | 1/30/2040 |
| ZALTRAP | ZIV-AFLIBERCEPT | 125418 | 10596141 | 5/15/2035 |
| ZINBRYTA | DACLIZUMAB | 761029 | 10016338 | 3/11/2033 |
| ZINBRYTA | DACLIZUMAB | 761029 | 10064938 | 3/11/2036 |
| ZINBRYTA | DACLIZUMAB | 761029 | 10085955 | 1/8/2033 |
| ZINBRYTA | DACLIZUMAB | 761029 | 10086046 | 5/8/2028 |
| ZINBRYTA | DACLIZUMAB | 761029 | 10111968 | 2/15/2032 |
| ZINBRYTA | DACLIZUMAB | 761029 | 10130632 | 11/23/2038 |
| ZINBRYTA | DACLIZUMAB | 761029 | 10261083 | 1/4/2033 |
| ZINBRYTA | DACLIZUMAB | 761029 | 10273252 | 4/28/2036 |
| ZINBRYTA | DACLIZUMAB | 761029 | 10279014 | 5/17/2039 |
| ZINBRYTA | DACLIZUMAB | 761029 | 10280230 | 9/10/2026 |
| ZINBRYTA | DACLIZUMAB | 761029 | 10316282 | 3/19/2033 |
| ZINBRYTA | DACLIZUMAB | 761029 | 10323013 | 4/23/2033 |
| ZINBRYTA | DACLIZUMAB | 761029 | 10329314 | 4/4/2036 |
| ZINBRYTA | DACLIZUMAB | 761029 | 10350178 | 1/8/2033 |
| ZINBRYTA | DACLIZUMAB | 761029 | 10369114 | 3/14/2033 |
| ZINBRYTA | DACLIZUMAB | 761029 | 10369124 | 4/30/2034 |
| ZINBRYTA | DACLIZUMAB | 761029 | 10376578 | 6/4/2032 |
| ZINBRYTA | DACLIZUMAB | 761029 | 10377832 | 4/25/2032 |
| ZINBRYTA | DACLIZUMAB | 761029 | 10407673 | 8/24/2036 |
| ZINBRYTA | DACLIZUMAB | 761029 | 10443100 | 5/22/2034 |
| ZINBRYTA | DACLIZUMAB | 761029 | 10449145 | 5/2/2033 |
| ZINBRYTA | DACLIZUMAB | 761029 | 10487098 | 4/4/2036 |
| ZINBRYTA | DACLIZUMAB | 761029 | 10500256 | 9/30/2034 |
| ZINBRYTA | DACLIZUMAB | 761029 | 10507262 | 5/11/2032 |
| ZINBRYTA | DACLIZUMAB | 761029 | 10562974 | 3/13/2033 |
| ZINBRYTA | DACLIZUMAB | 761029 | 10570103 | 3/15/2033 |
| ZINBRYTA | DACLIZUMAB | 761029 | 10577154 | 5/13/2029 |
| ZINBRYTA | DACLIZUMAB | 761029 | 10590182 | 2/24/2035 |
| ZINBRYTA | DACLIZUMAB | 761029 | 10656152 | 1/29/2029 |
| ZINBRYTA | DACLIZUMAB | 761029 | 10669337 | 7/25/2034 |
| ZINPLAVA | BEZLOTOXUMAB | 761046 | 10590182 | 2/24/2035 |
| ZULRESSO | BREXANOLONE | 211371 | 10117951 | 3/13/2029 |
| ZULRESSO | BREXANOLONE | 211371 | 10251894 | 11/27/2033 |
| ZULRESSO | BREXANOLONE | 211371 | 10322139 | 1/23/2033 |
| ZURAMPIC | LESINURAD | 207988 | 10183012 | 11/26/2028 |
