# Supplementary material for: NIH funding for patents that contribute to market exclusivity of drugs approved 2010–2019 and the public interest protections of Bayh-Dole
Source: PLoS One. 2023 Jul 26;18(7):e0288447. doi: 10.1371/journal.pone.0288447 (PMC10370755; doi:10.1371/journal.pone.0288447)
Supplement: S1 File — This method for identifying NIH-funded projects contributing to basic or applied research related to approved drugs was described in eMethods of Galkina Cleary et al. 2023, reproduced here with permission. (DOCX) [file pone.0288447.s004.docx]

**S1 File. Detailed description of the method.** This method for identifying NIH-funded projects contributing to basic or applied research related to approved drugs was described in eMethods of Galkina Cleary et al. 2023, reproduced here with permission.

*Identifying PMID related to drugs or drug targets*

*
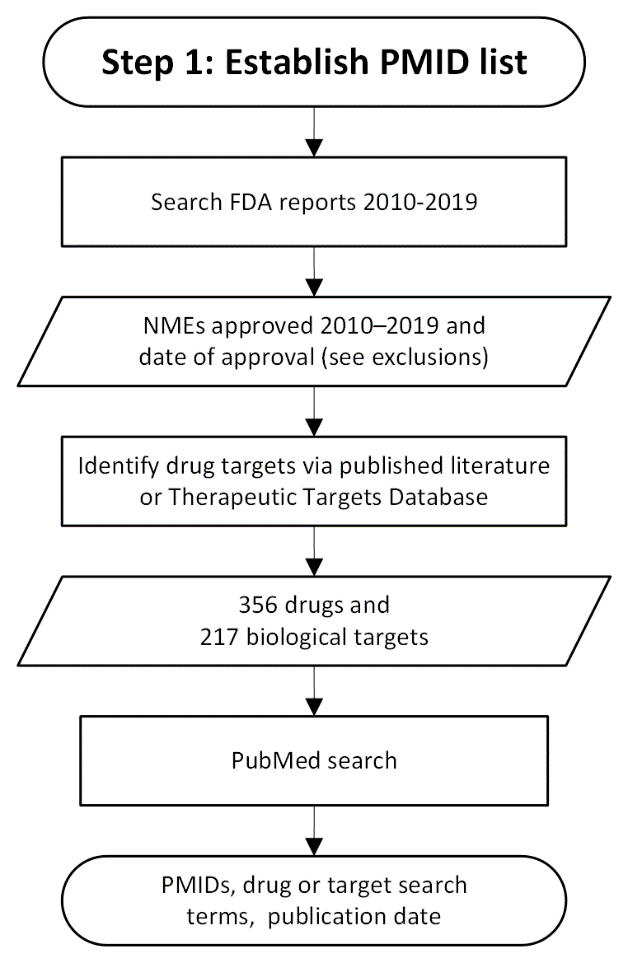
*New Molecular Entities (NMEs), including both NDAs and BLAs (type 1), approved by the FDA 2010–2019 and their date of first approval were identified from FDA reports from the Center for Drug Evaluation and Research (CDER) (<https://www.fda.gov/drugs/development-approval-process-drugs/new-drugs-fda-cders-new-molecular-entities-and-new-therapeutic-biological-products>) and from the Center for Biologics Evaluation and Research (CBER) (<https://www.fda.gov/vaccines-blood-biologics/development-approval-process-cber/2022-biological-approvals>). Products derived from blood or tissue, diagnostic agents, vaccines, and antimicrobials were excluded.

Drug targets were identified for these drugs from published literature (Eder, Sedrani et al. 2014, Santos, Ursu et al. 2017) or Therapeutic Targets Database (<https://db.idrblab.net/ttd/>) accessed January-June 2020 (Zhu, Han et al. 2010), giving a total of 356 drugs and 217 targets.

A drug was identified as first-in-class if it was the first product associated with a novel biological target as described by (Eder, Sedrani et al. 2014) or Lanthier et al., 2011 (Lanthier, Miller et al. 2013).

PubMed searches, including MeSH terms and Boolean modifiers, were performed for each drug and target (eTable 1 of Galkina Cleary et al. 2023) (May-June 2020) using the National Center for Biological Information (NCBI) Automated Term Mapping protocols (released in March 2020) (<https://www.nlm.nih.gov/pubs/techbull/ma20/ma20_pubmed_default.html>). The resulting publications for each drug and target were identified by their PubMed Identifier (PMID) along with the publication date and the search term (drug ID or target ID) used in the initial search identifying that PMID. PMIDs with publication dates after first FDA drug approval were excluded.

PMIDs identified in searching for a drug name are categorized as “DRUG” and are considered applied research related to that product. PMIDs identified in searching for a drug target, but not the associated drug, are categorized as “TARGET ONLY” and are considered basic research related to products associated with that target.

Note: PMIDs may be identified in multiple searches and are retained in the dataset in conjunction with each search. The resulting duplication is addressed in the final step of analyzing any specific subset of drugs or drug targets (see below).

*Identifying NIH funding for PMID*


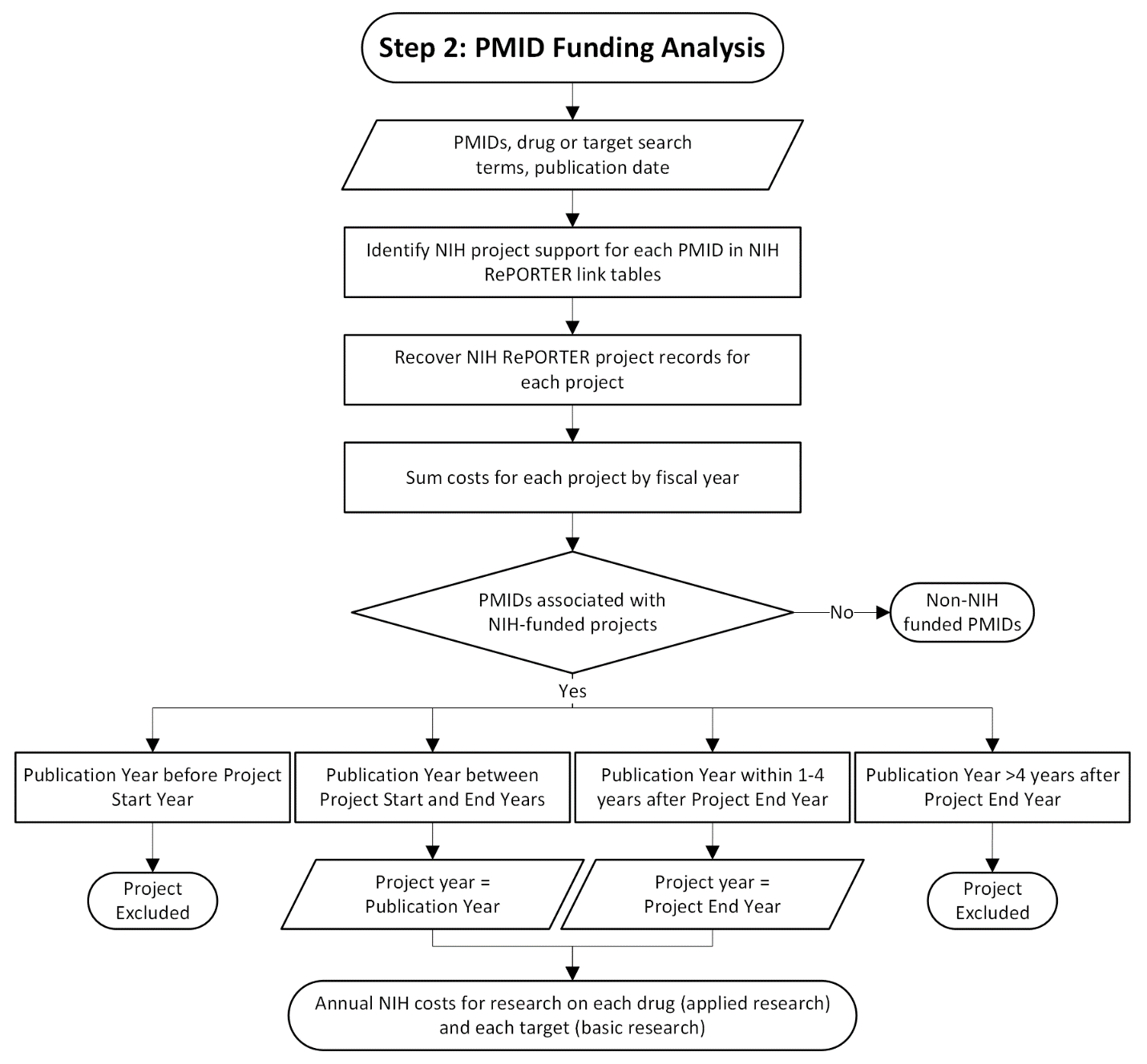
The NIH-funded grants (projects) and costs associated with each PMID were identified in the NIH RePORTER database (<https://reporter.nih.gov/>) accessed May 2020 by API (https://api.reporter.nih.gov/) and archived on a PostgreSQL server. This dataset comprised NIH-funded projects from January 2000-June 2020.

NIH RePORTER describes the funding for NIH projects, including the costs for each fiscal year, subprojects, and supplemental awards. For this analysis, each fiscal year of project funding is termed a “Project Year.” All costs for each project year were totaled and are termed “project year costs” or “costs.” The NIH RePORTER database also describes the project start year, project end year, and PMIDs citing funding from that award.

NIH costs associated with each PMID were identified through the following steps:

1. PMIDs were associated with one (or more) NIH-funded project cited as funding that research using the NIH RePORTER Publication “link tables”. Each project is identified by a Project Number comprising the Activity Code for that award, the institute making that award, and a unique identifier number. Data associated with each project includes the project start and end years and costs for each fiscal year, subproject, and supplemental award. Activity codes are described at (<https://grants.nih.gov/grants/funding/ac_search_results.htm>) and are further characterized by NIH funding program (<https://grants.nih.gov/grants/funding/funding_program.htm>).
2. For PMIDs with a publication date before the project start year of an associated project, no project year or NIH costs were assigned to the PMID.
3. For PMIDs with a publication date during the term of the award, the project year and project costs associated with the year of publication were assigned to the PMID.
4. For PMIDs published 1-4 years after the project end year, the project year and project costs associated with the end year were assigned to the PMID. This accounts for the estimated 3-year lag between NIH funding in RePORTER and publication dates (Boyack and Jordan 2011).
5. For PMIDs published >4 years after the end year of the project, no project years or costs were assigned.
6. Previously reported sensitivity analysis suggested that this method associates 86.3% of PMID with NIH costs, a fraction consistent with prior descriptions of false positive and negative findings in the RePORTER database (Zhou 2022).
7. Note that project years and costs may be assigned to multiple PMIDs and that each PMID may be represented more than once in the dataset (see above). The resulting duplication is addressed in the final step of analyzing any specific subset of drugs or drug targets (see below).
8. The number of PMIDs, project years, or project year costs associated with each drug were calculated from the number of PMIDs identified in the drug search, the project years associated with those PMIDs, and the costs associated with those project years after eliminating duplicates. This is categorized as applied research. Analyses were performed on all drugs and after elimination of costs beyond the 95^th^ percentile to outliers resulting from searches contaminated with ambiguous (generic) drug names. For example, clotting factors, hormones, and proteins such as alpha-1 antitrypsin.
9. The number of PMIDs, project years, or project year costs associated with each target were calculated from the number of PMIDs identified in the target search but not the drug search, the project years associated with those PMIDs, and the costs associated with those project years after eliminating duplicates. This is categorized as basic research. Analyses were performed on all drugs and after elimination of costs beyond the 95^th^ percentile to outliers resulting from searches contaminated with ambiguous target names. For example, CD-4, bcl-2, and EGFR, which are also used as adjectives.
10. Duplicate entries arise from PMID identified in more than one search and Project Years that support multiple PMID. The PMIDs, project years, and costs associated with a subset of drugs or their targets (i.e., individual drugs or targets, first to target drugs, a therapeutic area, or Activity Codes) are determined after eliminating duplicate PMIDs, project years, and costs within that category. Within each subset a PMID is considered applied research if it is associated with a drug search within that subset and is considered basic research if it is associated only with searches for targets within that subset. A project year and its costs are considered applied research if at least one PMID supported by that project year was identified by searching for a drug in that subset and is considered basic research if every PMID supported by that project year was identified by searching for a target in that subset. Note that a PMID, project year or its costs may be represented in multiple subsets.

Analysis was performed in SQL with data in a PostgreSQL database as previously described (Cleary, Beierlein et al. 2018, Cleary, Jackson et al. 2020, Cleary and Ledley 2020). Since completion of this work, this method has been replicated in Python code that is freely available at <https://github.com/BentleySciIndustry/NIH-Contribution-to-phased-clinical-development-of-drugs-approved-Supplemental-Data-Sharing.git>. This method has also been described in detail at [https://doi.org/10.5281/zenodo.7603331](https://zenodo.org/record/7603331#.Y-F3D3bMI2w). This method can also be accessed through a dashboard at <https://www.bentley.edu/centers/center-integration-science-and-industry/nih-funding-drug-innovation-dashboard>

**eReferences**

Boyack, K. W. and P. Jordan (2011). "Metrics associated with NIH funding: a high-level view." Journal of the American Medical Informatics Association 18(4): 423-431.

Cleary, E., M. J. Jackson and F. Ledley (2020). "Government as the First Investor in Biopharmaceutical Innovation: Evidence From New Drug Approvals 2010–2019." Institute for New Economic Thinking Working Paper Series(133).

Cleary, E. G., J. M. Beierlein, N. S. Khanuja, L. M. McNamee and F. D. Ledley (2018). "Contribution of NIH funding to new drug approvals 2010–2016." Proceedings of the National Academy of Sciences 115(10): 2329-2334.

Cleary, E. G. and F. D. Ledley (2020). "NIH funding for research underlying new cancer therapies." The Lancet Oncology 21(6): 755-757.

Eder, J., R. Sedrani and C. Wiesmann (2014). "The discovery of first-in-class drugs: origins and evolution." Nature Reviews Drug Discovery 13(8): 577-587.

Galkina Cleary, E., M. J. Jackson, E. W. Zhou and F. D. Ledley (2023). "Comparison of Research Spending on New Drug Approvals by the National Institutes of Health vs the Pharmaceutical Industry, 2010-2019." JAMA Health Forum 4(4):e230511.

Lanthier, M., K. L. Miller, C. Nardinelli and J. Woodcock (2013). "An improved approach to measuring drug innovation finds steady rates of first-in-class pharmaceuticals, 1987–2011." Health Affairs 32(8): 1433-1439.

Santos, R., O. Ursu, A. Gaulton, A. P. Bento, R. S. Donadi, C. G. Bologa, A. Karlsson, B. Al-Lazikani, A. Hersey and T. I. Oprea (2017). "A comprehensive map of molecular drug targets." Nature reviews Drug discovery 16(1): 19-34.

Zhou, E., Jackson, M.J., Ledley, F.D. (2022). "NIH Contribution to phased clinical development of drugs approved from 2010-2019: Tech Note." Center for Integration of Science and Industry, Bentley University.

Zhu, F., B. Han, P. Kumar, X. Liu, X. Ma, X. Wei, L. Huang, Y. Guo, L. Han and C. Zheng (2010). "Update of TTD: therapeutic target database." Nucleic acids research 38(suppl_1): D787-D791.
